# Catalytic reductions of nitroaromatic compounds over heterogeneous catalysts with rhenium sub-nanostructures

**DOI:** 10.1038/s41598-023-39830-y

**Published:** 2023-08-07

**Authors:** Piotr Cyganowski, Anna Dzimitrowicz, Mateusz M. Marzec, Sebastian Arabasz, Krystian Sokołowski, Anna Lesniewicz, Sylwia Nowak, Pawel Pohl, Andrzej Bernasik, Dorota Jermakowicz-Bartkowiak

**Affiliations:** 1 Department of Process Engineering and Technology of Polymer and Carbon Materials, Faculty of Chemistry, Wroclaw University of Science and Technology, Wybrzeze S. Wyspianskiego 27, 50-370 Wrocław, Poland; 2 Department of Analytical Chemistry and Chemical Metallurgy, Faculty of Chemistry, Wroclaw University of Science and Technology, Wybrzeze S. Wyspianskiego 27, 50-370 Wrocław, Poland; 3 Academic Centre for Materials and Nanotechnology, AGH University of Science and Technology, A. Mickiewicza Av. 30, 30-059 Kraków, Poland; 4 Łukasiewicz Research Network - PORT Polish Center for Technology Development, Stablowicka 147, 54-066 Wrocław, Poland; 5 Laboratory of Microscopic Techniques, Faculty of Biological Sciences, University of Wroclaw, H. Sienkiewicza 21, 50-335 Wrocław, Poland; 6 Faculty of Physics and Applied Computer Science, AGH University of Science and Technology, A. Mickiewicza Av. 30, 30-059 Kraków, Poland

**Keywords:** Catalysis, Nanoscale materials, Chemical engineering

## Abstract

Nitroaromatic compounds (NACs) are key contaminants of anthropogenic origin and pose a severe threat to human and animal lives. Although the catalytic activities of Re nanostructures (NSs) are significantly higher than those of other heterogeneous catalysts containing NSs, few studies have been reported on the application of Re-based nanocatalysts for NAC hydrogenation. Accordingly, herein, catalytic reductions of nitrobenzene (NB), 4-nitrophenol (4-NP), 2-nitroaniline (2-NA), 4-nitroaniline (4-NA), and 2,4,6-trinitrophenol (2,4,6-TNP) over new Re-based heterogeneous catalysts were proposed. The catalytic materials were designed to enable effective syntheses and stabilisation of particularly small Re structures over them. Accordingly, catalytic hydrogenations of NACs under mild conditions were significantly enhanced by Re sub-nanostructures (Re-sub-NSs). The highest pseudo-first-order rate constants for NB, 4-NP, 2-NA, 4-NA, and 2,4,6-TNP reductions over the catalyst acquired by stabilising Re using bis(3-aminopropyl)amine (BAPA), which led to Re-sub-NSs with Re concentrations of 16.7 wt%, were 0.210, 0.130, 0.100, 0.180, and 0.090 min^−1^, respectively.

## Introduction

As nitroaromatic compounds (NACs) are key contaminants of anthropogenic origin, decomposition of these compounds has attracted significant attention^1,2^. NACs are carcinogenic, tumourigenic, toxic, genotoxic, or reproductive-toxic^1,3–6^; thus, their common occurrence in all domains of the ecosystem and various waste sources and persistent nature pose a severe threat to human and animal lives^1,2,7^. Therefore, considerable efforts have been made to effectively remediate NACs^1,2,8,9^. Catalytic reduction under mild conditions is particularly important in this regard^1,2,9–11^ as the catalytic reductions of NACs result in aromatic amines (AAMs), which are fine chemical products and serve as key building blocks for the large-scale synthesis of pharmaceuticals^8,12^. Thus, the development of new catalysts that increase the efficiency of NAC reduction has attracted substantial attention^10,11,13,14^.

In this context, catalysts based on metal nanoparticles (NPs) are important because they significantly enhance the conversions of NACs owing to their larger surface areas as compared to those of their macroequivalents^10,11,13–15^. Recently, emerging nanomaterials (NMs) based on Re nanostructures (NSs) have been recognized as attractive alternatives to previously reported NPs based on Au and Pt group metals. Re offers unique chemistry to catalytic processes mainly due to its numerous oxidation states, namely, 0 (metallic), + 3, + 4, + 6, and + 7. Compared to other metals, Re forms stable species in these oxidation states that exhibit catalytic activities in hydrogenation reactions^16,17^. Nevertheless, only a few studies have been reported in this regard, and the results of these studies imply that ReNSs, Re^0^, O-doped ReNSs, and the blends of these materials are particularly useful for homogeneous and heterogeneous catalytic reductions of NACs^18–22^. These few examples indicate that ReNSs exhibit superior catalytic activities in the reductions of NACs such as nitrobenzene (NB)^21^, 4-NP^19–24^, 2-nitroaniline (2-NA)^21^, 4-NA^19,21,22^, 2,4,-dinitrophenol, and 2,4,6-trinitrophenol (2,4,6-TNP)^21^.

The tendency of Re to form ultra-small NSs (< 2 nm) is one of the promising aspects of Re-based nanocatalysis^19^. Development of these NSs may significantly boost the catalytic activities of Re-based nanocatalysts (NCats) because of the versatile catalytic chemistries of these NSs, and a wide spectrum of catalytically active Re species could be generated, rendering Re-based NMs perfect candidates for the efficient hydrogenations of NACs. However, it is difficult to apply the smallest ReNSs as NCats and evaluate the catalytic systems in which they are used. The obstacles encountered during the development of these systems include the limited stabilities of NSs and their susceptibilities to react with atomic oxygen and reactive oxygen species^21,23,24^. This renders the application, access to, and design of the morphologies of ReNSs challenging^16,17,19–24^. These issues can be overcome using matrices in which ReNSs can be synthesised, capped, and occluded^17^. This strategy facilitates the stabilisation of ReNSs and hinders direct contact between Re-active sites and atmospheric O_2_. Nevertheless, this strategy is not entirely satisfactory as O_2_ dissolved in the reaction environment is sufficient to oxidise Re^0^NPs^22^, thereby decreasing the catalytic activity of the as-prepared NCat. Therefore, we proposed an alternative approach that involved preparing ReNSs as a blend of catalytically active Re species at various oxidation states^24^. Then, we suggested that by synthesising and capping O-doped ReNSs that could reach subnanometric sizes, the possible decreases in the catalytic activities (arising from the intended O-doping effect) of NCats could be prevented. This approach will enable the convenient use of sub-nanostructures (sub-NSs) that are usually difficult to apply and allow us to avoid the O-doping effect of Re, which may occur during the development and use of ReNS-based NCats.

Accordingly, in this study, we proposed novel heterogeneous NCats containing subnanometric (< 1 nm) ReNSs consisting of different Re species for the catalytic hydrogenations of NB, 4-NP, 2-NA, 4-NA, and 2,4,6-TNP. The developed catalytic materials demonstrated unique characteristics arising from their in situ syntheses based on an anion-exchange reaction between ReO_4_^−^ and amino functionalities loaded onto polymer matrices and the capping and stabilisation of Re-active sites on these matrices. This approach is expected to effectively stabilise very small ReNSs in polymer matrices and lead to a synergistic effect between ReNSs and stabilising amino functionalities. Therefore, selected aliphatic, heterocyclic, and AAMs comprising chelating atoms, such as O and S, were used for the fabrication and capping of ReNSs. Because all Re species in ReNSs were expected to exhibit catalytic activities towards the hydrogenations of NACs^17,21–24^, in situ synthesis was performed in a way that allowed careful fabrication of very small ReNSs, which were blends of different O-doped ReNPs. Owing to subnanometric ReNSs and the synergistic effect between ReNSs and the polymer matrix, the resulting NCats demonstrated high catalytic activities towards the hydrogenations of NACs and better stabilities.

## Results and discussion

**Figure 1:**
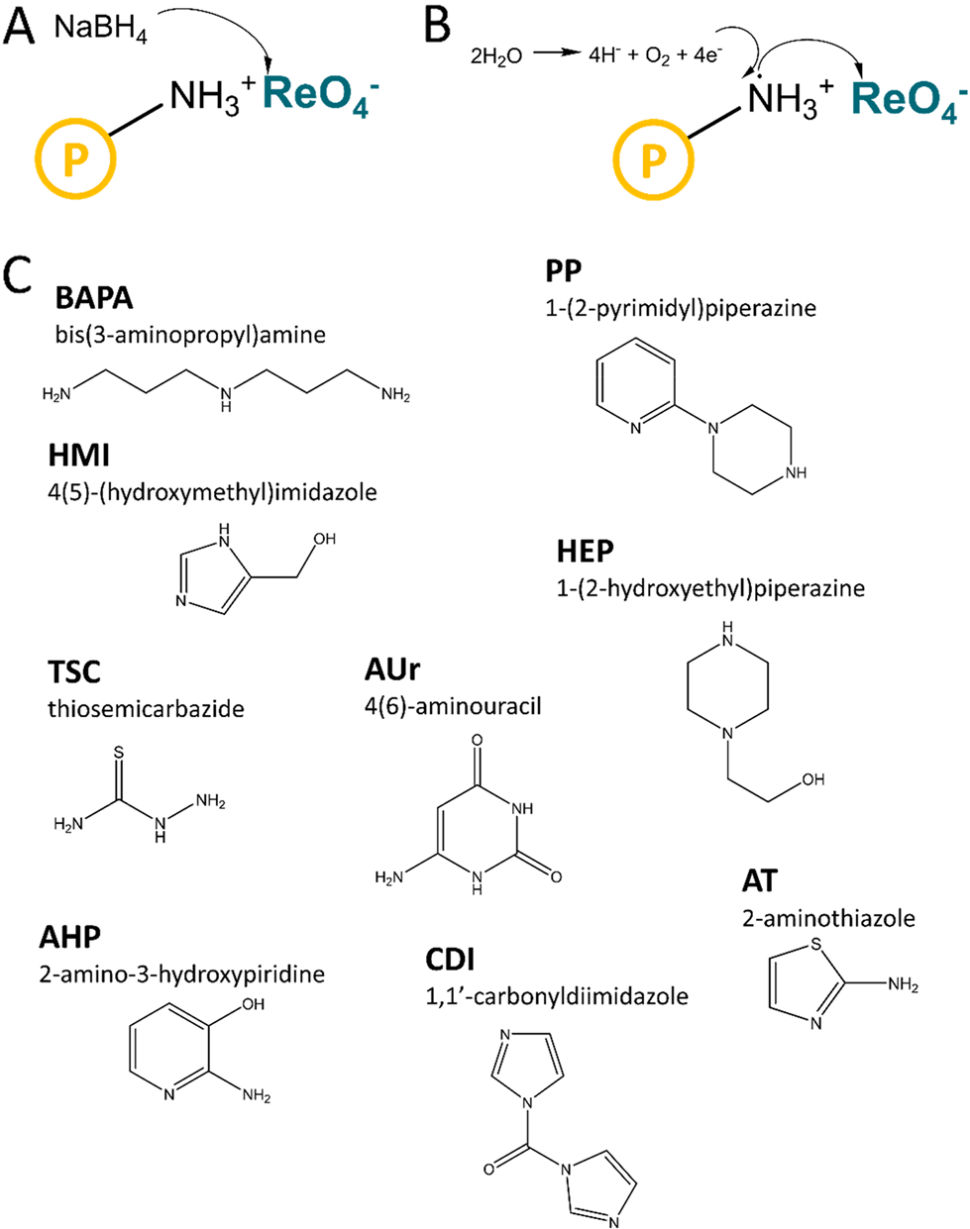
Schematics of the syntheses of Re nanostructures (NSs) in the polymer matrix using (**A**) a reducing agent and (**B**) reduction-coupled adsorption and (**C**) structures of the amines present in anion-exchange resins.

The new heterogenous catalysts base on polymeric matrices with stabilizing functionalities derived from bis(3-aminopropyl)amine (BAPA), 4(5)-(hydroxymethyl)imidazole (HMI), 1-(2-pyrimidyl)piperazine (PP), thiosemicarbazide (TSC), 2-amino-3-hydroxypiridine (AHP), 1-(2-hydroxyethyl)piperazine (HEP), 4(6)-aminouracil (AUr), 1,1′-carbonyldiimidazole (CDI) and 2-aminothiazol (AT). To the structure of these polymers, Re-reactive sites were introduced using two approaches, which are explained in Fig. 1. Briefly, the first approach involved the reduction of ReO_4_^−^ using NaBH_4_ as a reducing agent (Fig. 1A), while the second approach included the reduction of ReO_4_^−^ via the transfer of an electron from the N atom of the amino functionality to ReO_4_^−^ (Fig. 1B). The detailed discussion on the synthesis of catalysts is provided in Supplementary Information, Sect. S1. The catalyst samples were coded using abbreviated names of amines, with the prefix ^*ext*^*Re* or *Re* added for the samples obtained with the external reducing agent and reduction-coupled adsorption, respectively.

### Model reaction of 4-NP reduction

**Figure 2:**
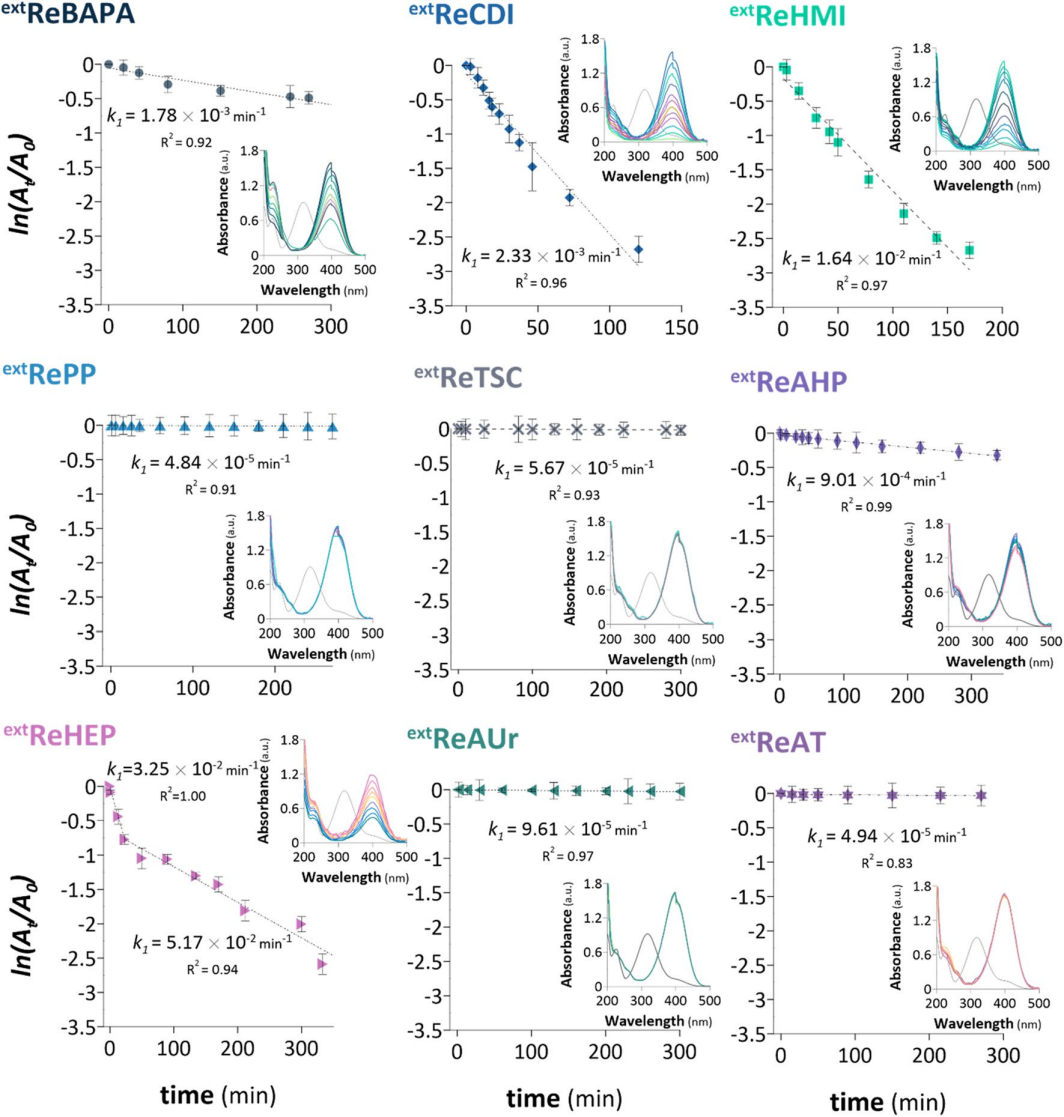
Pseudo-first-order kinetic plots and ultraviolet (UV)/visible (Vis) spectra for the reduction of 4-nitrophenol (4-NP) using catalysts containing ReNSs synthesised using an external reducing agent. Initial 4-NP concentration: 0.1 mmol L^−1^ and NaBH_4_ concentration: 0.1 mol L^−1^ (0.3 mL). The total reaction time and time intervals between subsequent UV/Vis spectra correspond to the data points shown in the pseudo-first-order kinetics plots.

Herein, the reduction of 4-NP to 4-aminophenol (4-AP) is chosen for modeling catalysts’ activity^25^. At first, the ^ext^Re samples were examined. The corresponding sets of pseudo-first-order kinetic plots and UV/Vis spectra for the catalytic reduction of 4-NP using each NCat sample are depicted in Fig. 2.

The obtained data (Fig. 2) implied that the model catalytic reaction appropriately obeyed the pseudo-first-order kinetics as correlation coefficients (*R*^2^) ranged from 0.83 to 1 (Fig. 2). The fabricated NCat samples were divided into two groups. The first group of samples comprised ^ext^ReBAPA, ^ext^ReCDI, ^ext^ReHMI, ^ext^ReAHP, and ^ext^ReHEP and demonstrated catalytic activities for the reduction of 4-NP to 4-AP. The highest pseudo-first-order rate constant (*k*_1_) (5.17 × 10^−2^ min^−1^) was observed in the case of ^ext^ReHEP, whereas the smallest *k*_1_ was observed for ^ext^ReAHP (9.01 × 10^−4^ min^−1^). This simultaneously influenced the 4-NP conversion yield (%). ^ext^ReHEP led to > 95% conversion of 4-NP within 300 min, whereas ^ext^ReCDI (*k*_1_ = 2.33 × 10^−3^ min^−1^) and ^ext^ReHMI (*k*_1_ = 1.64 × 10^−2^ min^−1^) resulted in 95 and 93% conversions of 4-NP within 125 and 175 min, respectively. ^ext^ReAHP was considerably less efficient as it led to only 27% conversion of 4-NP with *k*_1_ = 9.01 × 10^−4^ min^−1^. The second group of samples consisting of ^ext^RePP, ^ext^ReTSC, ^ext^ReAUr, and ^ext^ReAT exhibited insufficient catalytic activities for the reduction of 4-NP to 4-AP. The *k*_1_ values obtained for the second group of samples were approximately one order of magnitude lower than those achieved for the first group of samples and ranged from 4.84 × 10^−5^ to 9.61 × 10^−5^ min^−1^. Furthermore, negligible catalytic conversions of 4-NP (< 4%) were acquired using the second group of samples.

Adsorption of ReO_4_^−^ on anion-exchange resins more efficiently proceeds on amines with complex structures^26–28^. In this context, Certainly, there is a relationship between the observed catalytic activities of NCats and the structures of the amines incorporated into the polymer matrices of NCats (Fig. 1C). Samples containing amines with complex structures, namely, a clam-like structure (CDI), samples comprising substituents based on an aliphatic chain (HMI and HEP), or samples characterised by a long-chain aliphatic structure (BAPA) demonstrated significantly higher catalytic activities as compared to those of the samples containing short substituents (PP, TSC, AHP, AUr, and AT) (Fig. 1C). This indicated that the functionalities derived from CDI, HMI, HEP, and BAPA might provide a synergistic effect between the polymer matrix and the ReNSs, and thus, NCats with high catalytic activities would be obtained. This synergistic effect may not be the only reason for the differences observed between the apparent *k* of the catalytic reductions of 4-NP to 4-AP performed using different NCats. Several other factors, including the concentration of Re in the samples, average sizes of the fabricated ReNSs, and the oxidation state of Re in the synthesised ReNSs, might also influence the catalytic activities of NCats. The effects of these factors on the catalytic activities of NCats are discussed later in the manuscript.

**Figure 3:**
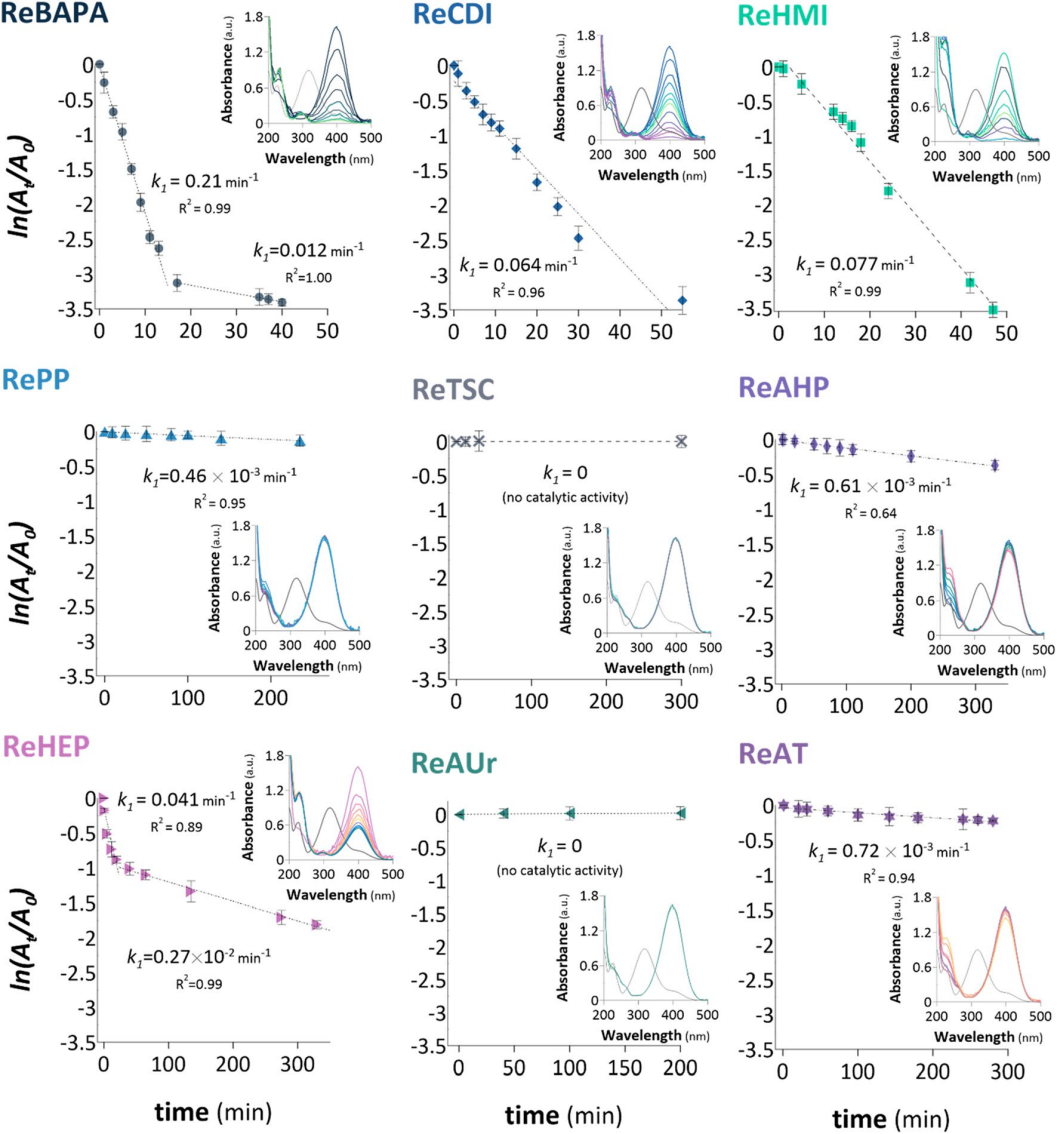
Pseudo-first-order kinetic plots and UV/Vis spectra for the reductions of 4-NP using different catalysts containing Re-active sites fabricated by reduction-coupled adsorption of ReO_4_^−^ on amino functionalities. Initial 4-NP concentration: 0.1 mmol L^−1^ and NaBH_4_ concentration: 0.1 mol L^−1^ (0.3 mL). The total reaction time and time intervals between subsequent UV/Vis spectra correspond to the data points depicted in the pseudo-first-order kinetic plots.

The catalytic activities of the samples obtained using second synthesis approach (Fig. 1B) were also analysed for the model reduction reaction, and the corresponding results are shown in Fig. 3. The highest catalytic activity was observed in the case of ReBAPA, which converted 97% 4-NP within 40 min with *k*_1_ = 0.012–0.21 min^−1^ (Fig. 3). The same 4-NP conversion yield was achieved within 47 min using ReHMI. However, in this case, *k*_1_ was 0.077 min^−1^. The remaining samples ReCDI, ReHEP, and ReAHP resulted in 97, 83, and 32% 4-NP conversions with *k*_*1*_ values of 0.064, 0.61 × 10^−3^, and 0.27–4.1 × 10^−2^ min^−1^, respectively. These data are consistent with the results obtained for the catalysts comprising ^ext^Re as the apparent catalytic activities of ReBAPA, ReCDI, ReHMI, ReHEP, and ReAHP (the first group of samples) are linked with the functionalities derived from BAPA, CDI, HMI, HEP, and AHP, respectively. RePP, ReTSC, ReAUr, and ReAT (the second group of samples) exhibited negligible or no catalytic activities for this reaction, similar to the cases of their equivalents acquired using NaBH_4_ (Figs. 2 and 3). This suggested that the conclusion regarding the synergistic effect between amino functionalities and ReNSs was valid.

To make comparing the data easier, Table S4 located in the Supplementary Information, Sect. S1.4 sets obtained rate constants, together with Turnover Frequencies (TOF) and maximum yields of NACs conversions for all of the samples. Based on Figs. 2 and 3 and Table S4 significant differences were observed between the catalytic activities of the NCat samples obtained using the two methods (Fig. 1A,B). Generally, the catalytic activities of the *Re* samples were substantially higher than those of the ^*ext*^*Re* samples. For instance, the *k*_*1*_ value calculated for ReBAPA (0.21 min^−1^) was an order of magnitude higher than the corresponding value evaluated for ^ext^ReBAPA (1.78 × 10^−3^ min^−1^). Similar phenomena were noticed for ReCDI and ^ext^ReCDI (*k*_1_ = 0.064 vs. 2.33 × 10^−3^ min^−1^), ReHIM and ^ext^ReHIM (*k*_1_ = 0.077 vs. 1.64 × 10^−2^ min^−1^), and ReHEP and ^ext^ReHEP (*k*_1_ = 0.041 vs. 3.25 × 10^−2^ min^−1^), respectively. These observations, combined with received TOF parameters (Table S4) suggest that this tendency is associated with the method applied for the syntheses of NCats. Thus, the origin of this tendency can be hypothesised to be the differences between the physiochemistries of ReO_4_^−^ reductions conducted by different methods. This could further influence the final morphologies of the NSs produced. It was speculated that the ReNSs formed using reduction-coupled adsorption might be considerably smaller and better dispersed than those constructed using NaBH_4_^29,30^. This could be the reason for the higher catalytic activities of the Re samples than those of the ^ext^Re samples. Therefore, the above-mentioned issue is comprehensively discussed in the next section.

### ReNSs in the polymer matrices

First, it was verified, whether the differences in the observed catalytic activities might have been caused by unequal concentrations of Re in the synthesized samples. The determined concentration of Re and detailed discussion is provided in Supplementary Information, Sect. S1.2 and Table S2. Briefly, the concentrations of Re in the *Re* samples were higher than those in the ^*ext*^*Re* samples. This explains why the *Re* samples demonstrated significantly higher catalytic activities than the ^*ext*^*Re* samples (Figs. 2 and 3). Further, the NCats demonstrated different concentrations of Re, ranging from 0 to 7.1% in the cases of the ^ext^Re samples and from 0 to 18.4% in the cases of the Re samples (Table S2). Among all the applied amines, AUr and TSC evidently prevented or suppressed the reduction of Re(VII) (*C*_Re_ values for these samples were ~ 0%, Table S2). Additionally, despite relatively high Re concentrations of ^ext^RePP, RePP, ^ext^ReAT, and ReAT (7.1, 17.0, 4.6, and 12.5%, respectively), these NCats exhibited negligible catalytic activities (Figs. 2 and 3); in contrast, other NCats with similar Re concentrations (for example, ^ext^ReHEP and ReHEP, Table S2) demonstrated outstanding catalytic activities. These observations confirm the synergistic effect between ReNSs and amino functionalities and allow us to conclude that the applied amine precisely regulates the synthesis routes of ReNSs irrespective of the method used.

**Figure 4:**
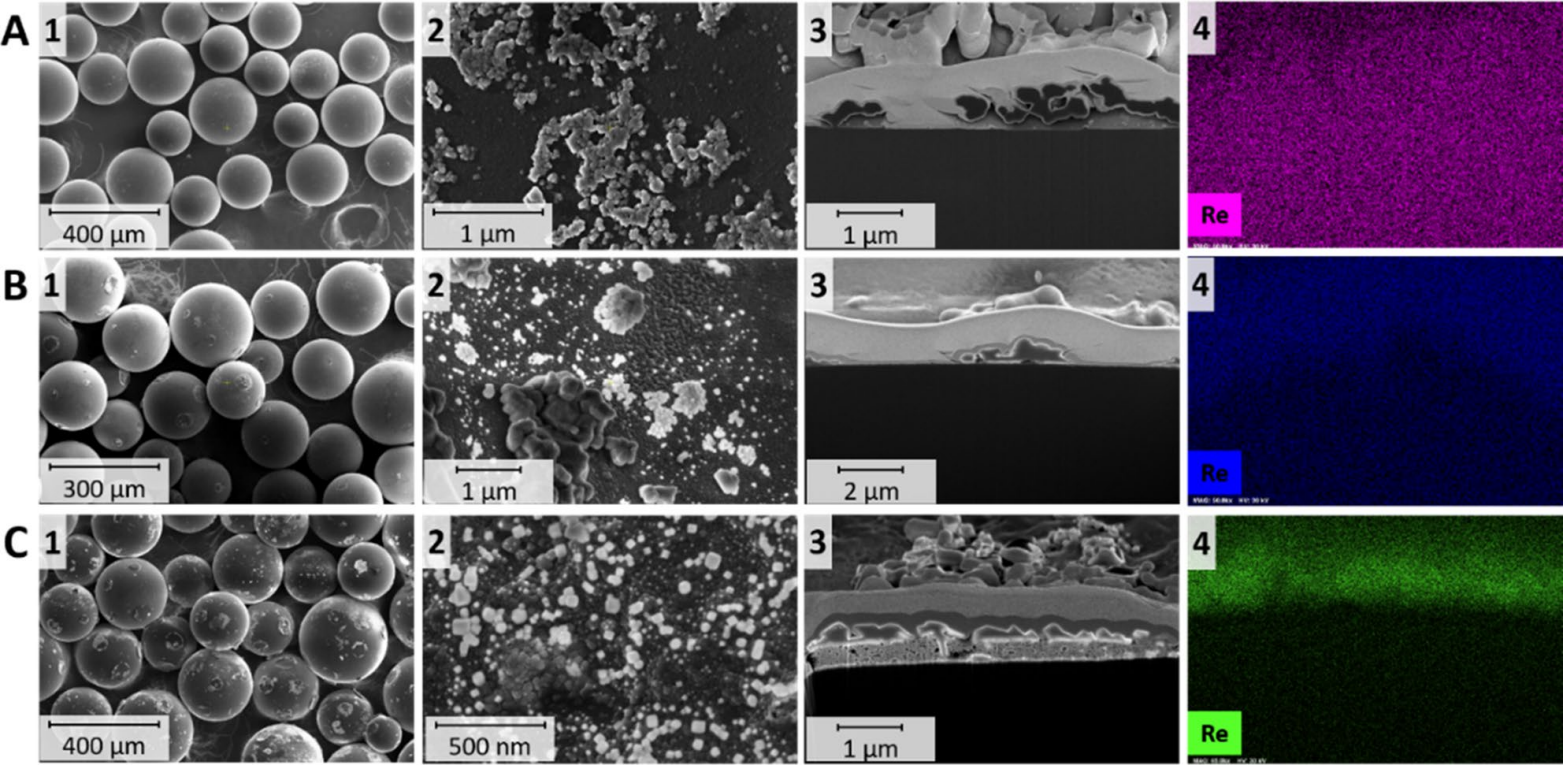
Ultra-high-resolution scanning electron microscopy images of (**A**) ReBAPA, (**B**) ^ext^ReBAPA, and (**C**) ^ext^RePP. (**1**) Morphologies of nanocatalyst (NCat) grains, (**2**) Morphologies of the NCat surfaces, (**3**) Focussed ion beam cross-sections, and (**4**) Energy-dispersive X-ray (EDX) maps of the cross-sections (towards Re).

^ext^RePP is covered with a large number of agglomerates derived from Re (as indicated by the EDX map) (Fig. 4C1). Although a closer observation (Fig. 4A2,B2, and C2) revealed the presence of ReNS agglomerates on the surfaces of all samples, the surfaces of ReBAPA and ^ext^ReBAPA had considerably less agglomerates. Observations of the polymer cross-sections revealed another phenomenon. The areas below the surfaces of the polymer grains were almost entirely clear in the cases of ReBAPA and ^ext^ReBAPA (Fig. 4A3,B3, respectively), whereas those in the case of ^ext^RePP comprised large particles and their agglomerates (Fig. 4C3). Simultaneously, the EDX mapping of Re on the cross-sections demonstrated that ReBAPA had a uniform distribution of Re from the surface to the centre of the polymer grain (Fig. 4A4), while within ^ext^ReBAPA (Fig. 4B4) the higher density of Re was observed in close proximity to the ^ext^ReBAPA surface. The absence of particles in the cross-sections of ReBAPA and ^ext^ReBAPA (Fig. 4A3,B3) implied that Re produced very small NSs, and UHR-SEM was unable to observe them. Thus, high-resolution transmission electron microscopy analysis (HRTEM) was conducted. Figure 5 depicts the HRTEM images of the cross-sections of ReBAPA, ^ext^ReBAPA, and ^ext^RePP as the representative samples.

Second, despite differences in Re concentrations, it was verified how amines, and the methods of syntheses influenced the morphology of the ReNSs. At first, the morphologies of the polymer samples were assessed via ultra-high resolution scanning electron microscopy (UHR-SEM); additional assessments of the polymer cross-sections (achieved using Ga-focused ion beam (FIB) and Xe-plasma FIB) and elemental mapping acquired using energy dispersive X-Ray spectrometer (EDX) were also performed. Figure 4 shows the SEM and UHR-SEM images of the representative samples ReBAPA, ^ext^ReBAPA, and ^ext^RePP. These samples were chosen because of the following reasons. The findings obtained for ReBAPA and ^ext^ReBAPA may explain why the two different methods of ReNS syntheses resulted in significantly different Re concentrations in the NCats. Moreover, combining these observations with those acquired for ^ext^RePP may clarify why ^ext^RePP exhibits negligible catalytic activity despite its higher Re concentration (Table S2) and why ^ext^ReBAPA outperforms it.

**Figure 5:**
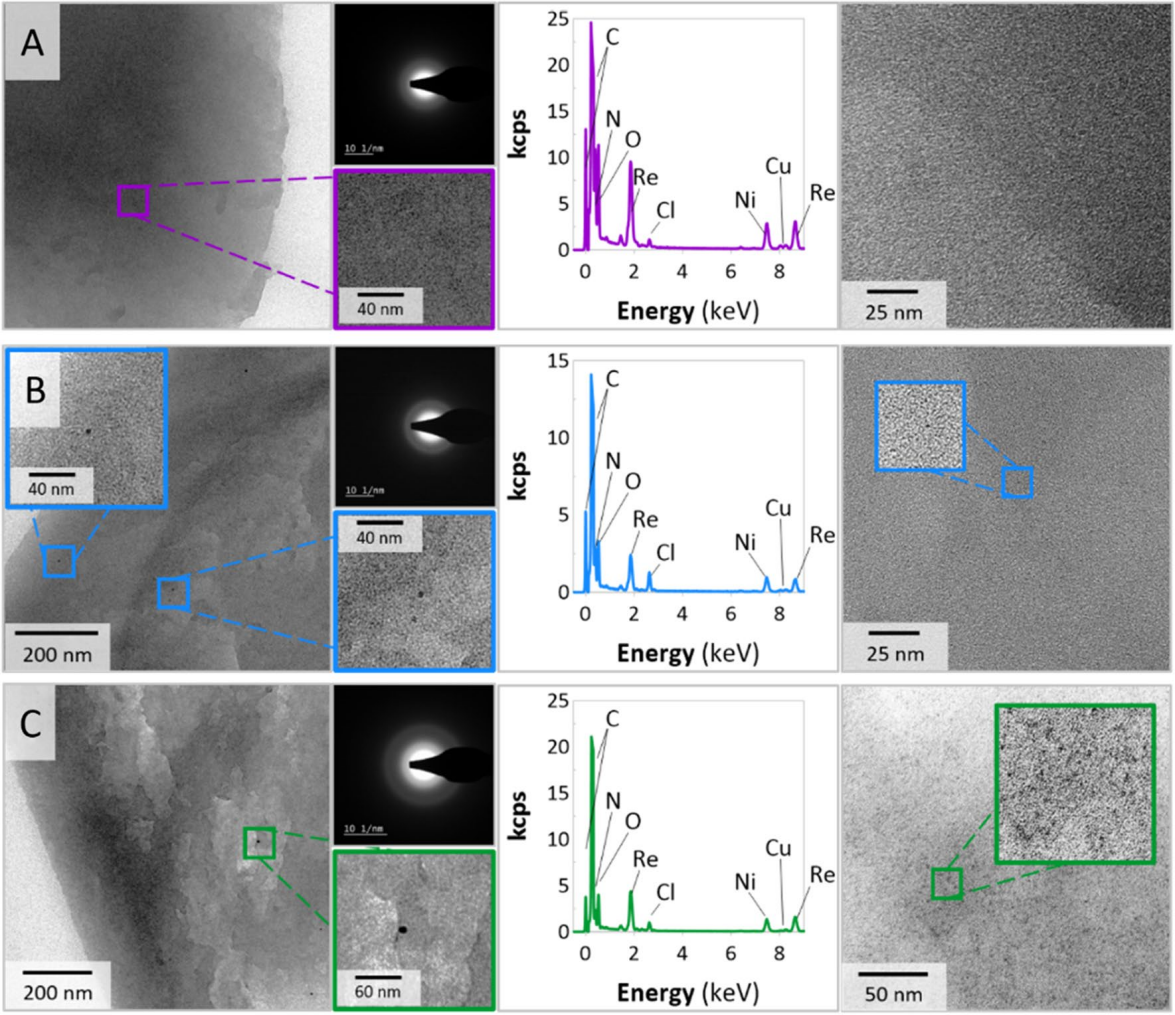
High- resolution transmission electron microscopy images, selected area electron diffraction patterns, and EDX spectra of (**A**) ReBAPA, (**B**) ^ext^ReBAPA, and (**C**) ^ext^RePP.

**Figure 6:**
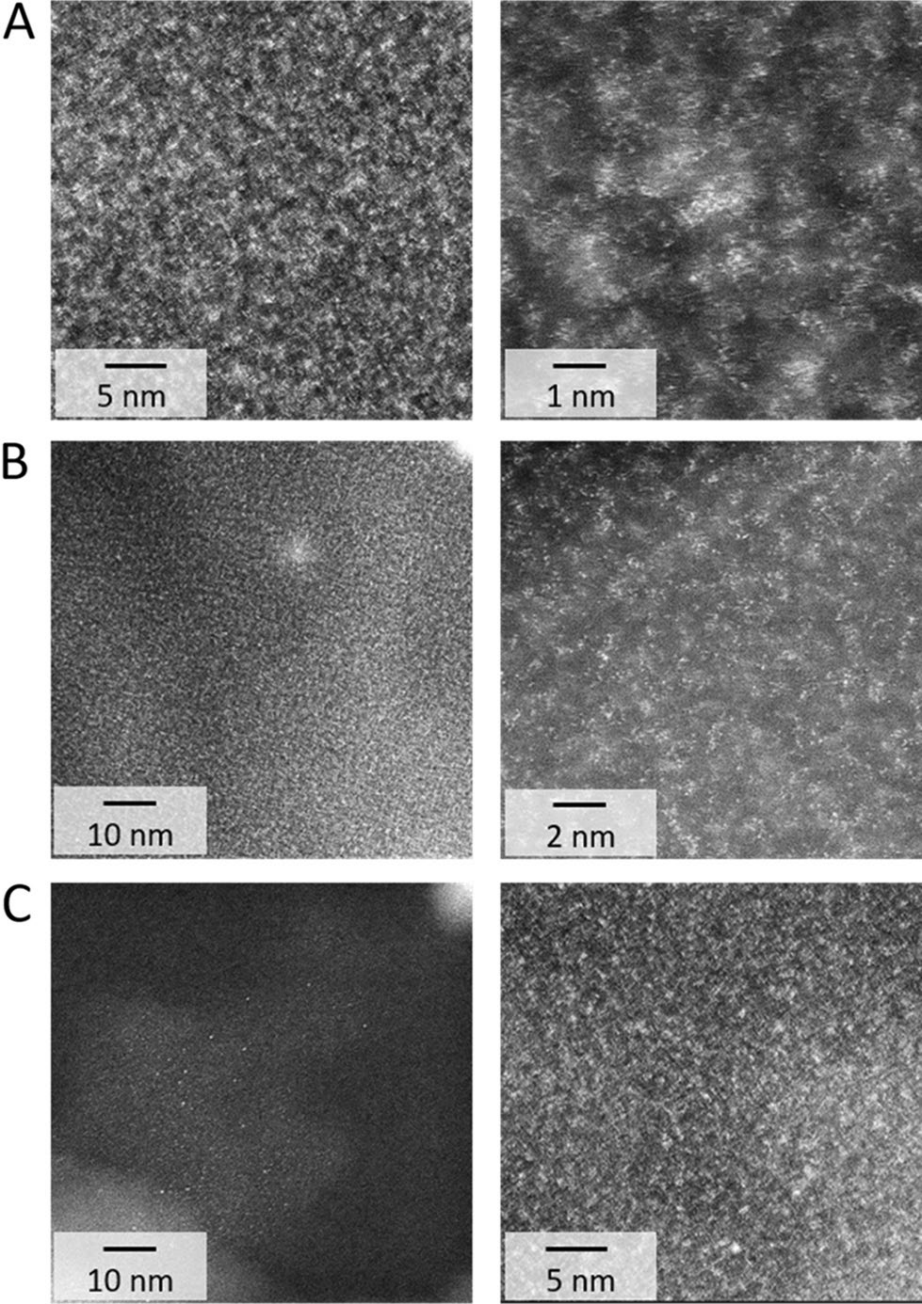
Scanning transmission electron microscopy images of (**A**) ReBAPA, (**B**) ^ext^ReBAPA, and (**C**) ^ext^RePP.

The observation of the NCat samples was difficult, even using HRTEM. The images of ^ext^RePP demonstrated some structures near the polymer grain surface (first panel) and in the inner part of the polymer grain (right panel) (Fig. 5C). According to the EDX spectra (Fig. 5), all these structures were based on Re. The particles situated near the polymer grain surface were bigger (sizes: ~ 2–10 nm), whereas the particles in the inner part of the polymer grain were smaller (sizes: 0.83 ± 0.52 nm, dark spots in Fig. 5C, right panel). The same phenomenon was observed for ^ext^ReBAPA (Fig. 5B). Nevertheless, in this case, the number of NSs was considerably lower than that of ^ext^RePP (Fig. 5B,C). The difference between the observed catalytic activities of ^ext^ReBAPA and ^ext^RePP led to further uncertainties as ^ext^ReBAPA outperformed ^ext^RePP in the catalytic reduction of 4-NP (Fig. 2). This suggested the primary role of BAPA in the syntheses and performances of NCats; however, a possible reason for this phenomenon could be deduced by investigating both samples containing BAPA functionalities (^ext^ReBAPA, ReBAPA). ^ext^ReBAPA comprised a small number of NSs, whereas ReBAPA contained no NSs (Fig. 5A). Some phase contrast was noticed near the polymer grain surface in Fig. 5A, which indicated the production of very small structures; nevertheless, HRTEM was not a suitable technique to examine these structures. The significantly high catalytic activity of ReBAPA than those of all other NCats further validates the hypothesis that some Re-based structures must have formed in ReBAPA (Figs. 2 and 3). This finding necessitates further investigation of ReBAPA. Thus, additionally, HRTEM in the scanning-transmission (STEM) mode using large magnifications and a high angle annular dark field detector (HAADF) was performed. Figure 6 shows the corresponding images.

Large magnifications, along with the use of the STEM-HAADF mode, allowed the observation of single Re atoms (bright spots in the images depicted in Fig. 6). These atoms occasionally grouped in sub-NSs, implied by the local increase in Re density (larger bright areas). Single NSs were observed for ^ext^ReBAPA and ^ext^RePP (Figs. 5B,C and 6B,C, left panels), whereas the situation was completely different in the case of ReBAPA, which consists of large areas entirely loaded with Re atoms that tended to group on the << 1 nm scale were noticed (Fig. 6A, right panel). The corresponding images might have been acquired at the very beginning of the formation of these structures, which would have been “frozen” by the polymer matrix itself. This was ascribed to the effective stabilisation of ReO_4_^−^ by amines, which might have prevented the growth of reduced Re forms. According to these findings, it was concluded that although BAPA prevented the formation of NSs, it facilitated the production of Re-sub-NSs, which were catalytically active and substantially boosted the catalytic activities of catalysts in the reduction of 4-NP. This conclusion might be extended to other samples that exhibited higher catalytic activities than those of other samples. Thus, CDI, HMI, and HEP functionalities must also contribute to the formation of Re-sub-NSs.

### Re oxidation states in the polymer matrices

The selected area energy diffraction (SAED) patterns obtained during HRTEM (Fig. 5) indicated amorphous morphologies of the investigated materials. These observations could indicate the existence of Re–Re bonds in ReNSs^21,31,32^. Nevertheless, Re can form various stable species^16,17^, and the Re-sub-NSs observed by STEM-HAADF exhibited amplitude contrasts originating from the lack of long-range orderings of the particles. Therefore, SAED was not sufficient to draw any binding conclusions in this case. Therefore, X-Ray photoelectron spectroscopy (XPS) analysis was performed to determine the types of Re species in the synthesized samples.

Surface concentrations of chemical bonds achieved by fitting the XPS data for all analyzed samples are presented in Table S3. Furthermore, the methodology for determining specific bonds is systematically described in Sect. S2 of the supplementary information. Moreover, all the acquired spectra are provided in S3.1 (survey scans) and S3.2 (high-resolution spectra). Re 4*f* spectra (Section S1.2) were fitted with up to three doublet structures (doublet separation *f*_7/2_–*f*_5/2_ = 2.43 eV) with the first 4f_7/2_ line centred at 42.4 eV, which implied the presence of Re^4+^ similar to that in ReO_2_. The second 4*f*_7/2_ line centred at 44.0 eV demonstrated the existence of Re^6+^ similar to that in ReO_3_, and the last 4*f*_7/2_ line at 46.1 eV indicated the presence of Re^7+^ similar to that in Re_2_O_7_^33,34^. Based on these results, it was concluded that the reduction of ReO_4_^−^ was indeed successful. Additionally, note that Re^7+^ in Re_2_O_7_ was different from Re^7+^ in NH_4_ReO_4_ (precursor).

Atomic% Re concentrations provided in Table S3 are consistent with those determined in Table S2. Generally, the concentrations of Re in ^ext^Re samples were lower than those in the Re samples. Moreover, the atomic% concentrations of Re^4+^, Re^6+^, and Re^7+^ were typically higher in the second group of samples (Table S3). All these findings agreed with the observed *k* values of 4-NP reduction for the Re samples; as evidenced in the model reaction, the Re samples outperformed the ^ext^Re samples (Figs. 2 and 3).

ReBAPA, ReCDI, ReHMI, ReHEP, ^ext^ReBAPA, ^ext^ReCIM, ^ext^ReHMI, and ^ext^ReHEP efficiently reduced ReO_4_^−^ as Re^4+^, Re^6+^, and Re^7+^ in the form of Re_2_O_7_ were present in these samples. However, RePP, ReTSC, ReAHP, ReAT, ^ext^RePP, ^ext^ReTSC, ^ext^ReAHP, and ^ext^ReAT also contained these forms of Re and exhibited inferior or no catalytic activities. This phenomenon can be explained by comparing the data provided in Tables S2 and S3. Based on the XPS results, the atomic% Re concentrations of ^ext^RePP and ^ext^ReTSC, which were not catalytically active, were higher than that of ^ext^ReBAPA, which outperformed ^ext^RePP and ^ext^ReTSC in the catalytic reduction of 4-NP. Hence, the following conclusions can be drawn: First, Re in the ^ext^Re samples is generally concentrated near the polymer grain surface. Second, according to the atomic% Re concentrations (Table S3), it can be specified that the same phenomenon (namely, the location of Re on the sample surface) is induced by the application of PP, TSC, AHP, and AT; the samples obtained using these amines contain more Re atoms on their surfaces than those in the cases of other catalytically active samples (Table S3). These results are consistent with the UHR-SEM, HRTEM, and EDX results. This indicated that the application of BAPA, CDI, HMI, and HEP typically resulted in the formation of several Re species in the whole volume of the sample, and therefore, the production of Re-sub-NSs in the inner part of the polymer grain was possible. This further confirmed the abovementioned synergistic effect between ReNSs and amino functionalities, which was the key to achieving high catalytic activities.

### Catalytic reductions of NACs

**Figure 7:**
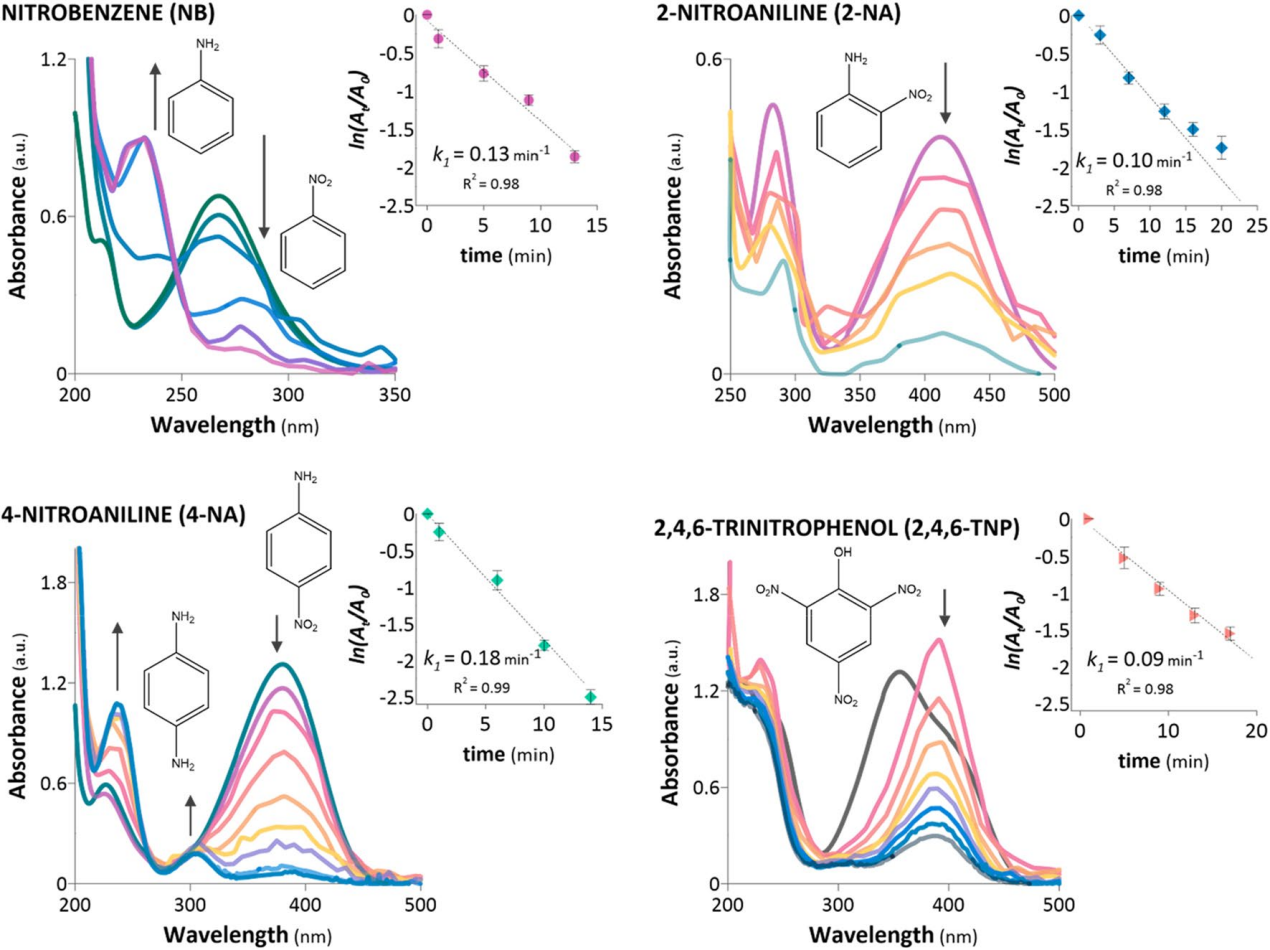
Reductions of NB, 2-NA, 4-NA, and 2,4,6-TNP using ReBAPA. Initial concentration of the nitroaromatic compound (NAC): 0.1 mmol L^−1^ and NaBH_4_ concentration: 0.1 mol L^−1^ (0.3 mL). The total reaction time and time intervals between subsequent UV/Vis spectra correspond to the data points presented in the first-order kinetic plots.

The results of catalytic activity analysis and the assessments of the characteristics of ReNSs and Re-sub-NSs implied a direct relationship among the amines present in the polymer matrix, properties of Re, and catalytic activities of the synthesised NCats. Although all the NCat samples containing functionalities derived from BAPA, CDI, HMI, and HEP were effective for the hydrogenation of 4-NP to 4-AP, ReBAPA outperformed all other NCats, leading to > 95% conversion of 4-NP in just 15 min with a *k*_1_ of 0.21 min^−1^ (Fig. 3). Therefore, ReBAPA was chosen for further analyses. Figure 7 depicts the first-order kinetic plots acquired for the reductions of NB, 2-NA, 4-NA, and 2,4,6-TNP using ReBAPA and the UV/Vis spectra for each reduction.

The obtained spectra demonstrated that ReBAPA enabled the reduction of NB to aniline^35^, the reduction of 4-NA to 1,4-diaminobenzene^36^, and the reductions of 2-NA^37^ and 2,4,6-TNP^38^. Although the highest *k*_*1*_ was acquired for the model 4-NP reduction, the *k*_1_ values of the catalytic processes performed using other NACs ranged from 0.09 to 0.18 min^−1^. As these differences were not considerable, these might have originated from the reaction itself rather than from the different catalytic activities of ReBAPA for different NACs. For instance, the *k*_*m*_ values calculated for the reductions of 4-NP and 2,4,6-TNP were 4.77 and 1.67 g^−1^ min^−1^, respectively. This almost three-fold difference between the *k*_*m*_ values for 4-NP and 2,4,6-TNP was proportional to the number of –NO_2_ groups present in the structures of 4-NP (one group) and 2,4,6-TNP (three groups). Nevertheless, this observation did not explain why the catalytic activity of ReBAPA was slightly lower for 2,4,6-TNP than those in the cases of other NACs. Consequently, the yields (%) of NAC reductions, along with the *TOF* values at 20, 50, 70, and 80% NAC reduction, were examined and detailed in Supplementary Information, Sect. S1 and Fig. S1. Based on these, it might be suggested that ReBAPA revealed similar catalytic activity towards each NAC (the detailed discussion is provided in Supplementary Information, Sect. S1.4).

### Present study versus other Re-based catalysts

Up to date, few studies have been reported on the application of homogeneous and heterogeneous Re-based NCats in the hydrogenations of NACs, which are summarised in Table 1. The results of this study confirmed that the heterogeneous NCat ReBAPP developed herein was more efficient than the homogeneous catalysts based on Re nanoclusters^19^ and raw ReNSs^23^. Additionally, the calculated *k*_*1*_ values were similar to those obtained using hybrid catalysts comprising Ag and AuNPs anchored on ReS_2_ nanosheets^31^. Nevertheless, ReBAPP also outperformed PtNPs immobilised on ReS_2_ in the reductions of 4-NP and 2-NA^31^. Although homogeneous NCats based on Re are rare, heterogeneous NCats based on Re are even rarer. When this manuscript was written (July 2023), a review of the literature revealed that few studies were reported on the application of Re-based heterogeneous NCats in the hydrogenations of NACs. In our previous studies, we attempted to optimise the structures of ReNSs by synthesising Re-based NMs with Re^0^^22^ or O-doped ReNSs^24^. Although the first approach resulted in Re^0^NPs with high catalytic activities, it did not offer catalyst stability as the Re^0^NPs interacted with O and thus lost their catalytic activities^22^. In contrast, the other approach based on reduction-coupled adsorption enabled the fabrication of O-doped ReNSs with slightly lower catalytic activities and higher stabilities than those of the abovementioned Re^0^NPs in the catalytic reduction of 4-NP^24^.

**Table 1:** Comparison of the performances of various ReNS-based NCats in the catalytic hydrogenations of NACs.

	NAC^a^	*k*_*1*_^b^	Refs
Homogeneous catalysts			
Re-nanocluster	4-NP	0.06	^19^
O-doped raw ReNSs	4-NP	0.160	^23^
AgNPs/ReS_2_ nanosheets	4-NP	0.306	^33^
	NB	0.450	
	2-NA	0.852	
AuNPs/ReS_2_ nanosheets	4-NP	0.318	
	NB	0.312	
	2-NA	0.402	
PtNPs/ReS_2_ nanosheets	4-NP	0.030	
	NB	0.216	
	2-NA	0.030	
Heterogeneous catalysts			
Re^0^NPs immobilised on			^22^
1,1′-Carbonyldiimidazole	4-NP	0.282	
	4-NA	0.329	
ReNSs with O-doped Re immobilised on			^24^
1-(2-hydroxyethyl)piperazine		0.205	
1,4-bis(3-aminopropyl)piperazine	4-NP	0.234	
1,1′-Carbonyldiimidazole		0.293	
Blend of O-doped ReNSs and Re-sub-NSs immobilised on			Present work
bis(3-aminopropyl)amine	4-NP	0.210	
	NB	0.130	
	2-NA	0.100	
	4-NA	0.180	
	2,4,6-TNP	0.090	

^a^Nitroaromatic compound; 4-NP: 4-nitrophenol; NB: nitrobenzene; 2-NA: 2-nitroaniline; 4-NA: 4-nitroaniline; 2,4,6-TNP: 2,4,6-trinitrophenol.

^b^Pseudo-first-order rate constant (min^−1^).

## Conclusions

In this study, we propose unique NCats loaded with ReNSs and Re-sub-NSs that can efficiently reduce NACs under mild conditions. The findings herein revealed that the unique reduction-coupled adsorption of ReO_4_^−^ on amino functionalities should be preferred for the fabrication of ReNSs rather than the approach involving the use of an external reducing agent. Reduction-coupled adsorption eliminates the application of toxic reducing agents and leads to NCats with Re-sub-NSs, which significantly boost the catalytic activities of heterogeneous catalysts.

Results indicated a synergistic effect between the ReNSs and the amino functionalities present in the polymer matrix. The amino functionalities with complex structures enabled efficient stabilisation of Re atoms, resulting in Re-sub-NSs. Moreover, a synergistic effect was noticed between NCats and the amines applied for their stabilisation. The amino functionalities with complex structures provided high catalytic activities to NCats. The mechanism of ReO_4_^−^ reduction was simple and did not require additional preparation or strict control over the synthesis conditions. Consequently, ReO_4_^−^ present in the polymer matrix formed Re^4+^, Re^6+^, and Re^7+^ O-doped species, which exhibited outstanding catalytic activities when combined with a synergistic amine. The type of Re species generated did not influence the catalytic activities of NCats as the corresponding ReNSs and Re-sub-NSs were versatile. In this regard, the kind of amine was the only limiting factor affecting the catalytic activities of NCats. Using a selected amine, the sizes of the structures formed by Re can be regulated, facilitating the development of NCats with high catalytic activities.

This study provides further insight into these unique NMs. First, Re-based NCats can be applied to other reductions instead of only the model reaction as this type of NCats effectively reduce other NACs in addition to 4-NP. Second, because of the versatility of ReNSs, the forms in which Re-based NPs are produced do not influence the catalytic activities of NCats, and thus, ReNSs can be prepared via simpler procedures. Third, the application of an appropriate amine enables the formation of Re subnanometric structures that further boost the catalytic activities of Re-based materials. Fourth, owing to the synergistic effect between ReNSs and amino functionalities and the affinity of the polymer matrix to NACs, the catalytic activities of the developed NCats can be enhanced or tailored. Thus, based on the results of this study, a process for NAC reduction that can be performed in a flow mode with the simultaneous production of AAMs can be envisioned.

## Material and methods

Detailed list of standard materials, instrumentation, and synthetic protocols is provided in Supplementary Information, Sect. S1.

### Materials, instruments and analyses methods

For the synthesis of polymeric base (anion exchange resins) the following materials were used: vinylbenzyl chloride (VBC), divinylbenzene (DVB), benzoyl peroxide, poly(vinyl alcohol), bis(3-aminopropyl)amine (BAPA), 4(5)-(hydroxymethyl)imidazole (HMI), 1-(2-pyrimidyl)piperazine (PP), thiosemicarbazide (TSC), 2-amino-3-hydroxypiridine (AHP), 1-(2-hydroxyethyl)piperazine (HEP), 4(6)-aminouracil (AUr), 1,1′-carbonyldiimidazole (CDI) and 2-aminothiazol (AT). The loading of ReNSs, the ammonium perrhenate (NH_4_ReO_4_) was used. For the catalytic studies selected NACs, that is, NB, 4-NP, 2-NA, 4-NA, and 2,4,6-TNP, together with NaBH_4_ were used.

The concentration of N (*Z*_*N*_) derived from amino functionalities was determined by elemental analysis using. Morphologies of the polymer samples were assessed by ultra-high-resolution scanning electron microscopy (UHR-SEM) aided by Ga-focused ion beam (Ga-FIB) and Xe-PFIB and energy-dispersive X-ray (EDX) spectrometers. Moreover, ReNSs loaded onto different polymers were characterised by high-resolution transmission electron microscopy (HRTEM) aided by a selected area electron diffractometer, EDX spectrometer, and high-angle annular dark-field (HAADF) detector in the scanning transmission electron microscopy (STEM) mode. Concentrations of Re in the NCat samples were determined via inductively coupled plasma optical emission spectrometry (ICP-OES). Before analysis, the ReNS-loaded polymer samples were washed with a 1% NH_4_OH solution to remove unreduced ReO_4_^−^. The Re oxidation states were determined using X-ray photoelectron spectroscopy (XPS) by obtaining high-energy resolution spectra for Re 4*f* region. The catalytic reaction rate was monitored by ultraviolet/visible (UV/Vis) spectrophotometry. The reaction rates were determined using the corresponding UV/Vis spectra by calculating the maximum absorbance of NACs at a specific wavelength (λ_max_). For the reaction, 2.5 mL of a NAC solution (0.1 mmol L^−1^) was added to a 3 mL quartz cuvette. Subsequently, 0.3 mL NaBH_4_ solution (0.1 mol L^−1^) was introduced, and the absorbance at λ_max_ = 400, 275, 410, 380, 390 nm was measured for 4-NP, NB, 2-NA, 4-NA, and 2,4,6-TNP, respectively. Based on the decrease of absorbance, − ln(*A*_*t*_/*A*_0_) versus *t* plots were constructed (where *A*_*t*_ is the absorbance at time* t* and *A*_0_ is the absorbance at the beginning of the process), and rate constants (*k* (min^−1^)) were evaluated from the slopes of these plots. Next, the *k* values were recalculated to obtain the mass-normalised rate constants *k*_*m*_ (g^−1^ min^−1^). Finally, the mass-dependent activity of each catalyst was estimated using turnover frequency (*TOF*, min^−1^), defined as *n*_*4-NP*_ × *r* × *n*_*Re*_^−1^ × t^−1^, where *n*_*4-NP*_ and *n*_*Re*_ represent the number of moles of 4-NP and ReNSs at the start of the process, respectively, *t* is the process time (min), and *r* is the yield (%) of reduction at which *TOF* is calculated.

### Syntheses of Re-loaded NCats

At first, the polymer matrix, namely, VBC-co-DVB (copolymer), was fabricated by suspension polymerisation. Then, the resultant copolymer VBC-co-DVB was separately modified by selected amines, that is, BAPA, 1,1′-carbonyldiimidazole (CDI), HMI, PP, TSC, AHP, HEP, AUr, and AT. Subsequently, the resulting anion-exchange resins (0.1 g) were mixed with solutions (50 mL, 500 mg Re L^−1^) containing NH_4_ReO_4_ in 0.1 mol L^−1^ HCl. This led to anion exchange between amino functionalities and ReO_4_^−^. Thereafter, two approaches were used to synthesise and stabilise ReNSs in the polymer matrix (Fig. 1).

The first approach involved the reduction of ReO_4_^−^ using NaBH_4_ as a reducing agent. Typically, the Re(VII)-loaded anion-exchange resins were separated from the precursor solution by filtration and washed with water on a fritted-glass funnel followed by introduction into 50 mL water comprising 0.1 mol L^−1^ NaBH_4_. This resulted in the immediate syntheses and precipitations of ReNSs in the polymer matrix. After 24 h, each resin was separated by filtration, washed with water, and used in its swollen state. The second approach included the reduction of ReO_4_^−^ via the transfer of an electron from the N atom of the amino functionality to ReO_4_^−^ (Fig. 1B). Subsequently, the Re(VII)-loaded anion-exchange resins were separated from the precursor solution, washed with water, and stored in 50 mL water for 4 weeks. This caused gradual reduction and precipitation of ReNSs. Although both methods involved in situ reduction, the second method provided a stable concentration gradient between the reactive groups (amino functionalities) and ReO_4_^−^ at the solid/liquid interface without any external reducing agent; thus, the resulting ReNSs were expected to be smaller and appropriately dispersed in the polymer matrix as compared to the cases of NMs achieved using NaBH_4_.

NCat samples were named according to the abbreviations of the amines present in the prepared polymer matrices, and the prefixes ^ext^Re and Re were used to represent ReNSs obtained using an external reducing agent (for example, ^ext^ReBAPA) and reduction-coupled adsorption (for instance, ReBAPA), respectively.

### Supplementary Information

Supplementary Information.

## Data Availability

All data generated or analysed during this study are included in this published article and its supplementary information.
