# Supplementary material for: Catalytic reductions of nitroaromatic compounds over heterogeneous catalysts with rhenium sub-nanostructures
Source: Sci Rep. 2023 Aug 7;13:12789. doi: 10.1038/s41598-023-39830-y (PMC10406812; doi:10.1038/s41598-023-39830-y)
Supplement: Supplementary file 1 — Supplementary Information. [file 41598_2023_39830_MOESM1_ESM.docx]

Supplementary information

**Catalytic reductions of nitroaromatic compounds over heterogeneous catalysts with rhenium sub-nanostructures**

Piotr Cyganowski^a*^, Anna Dzimitrowicz^b^, Mateusz M. Marzec^c^, Sebastian Arabasz^d^, Krystian Sokołowski^c^, Anna Lesniewicz^b^, Sylwia Nowak^e^, Pawel Pohl^b^, Andrzej Bernasik^c,f^, Dorota Jermakowicz-Bartkowiak^a^

^a^Department of Process Engineering and Technology of Polymer and Carbon Materials, Faculty of Chemistry, Wroclaw University of Science and Technology, Wybrzeze S. Wyspianskiego 27, 50-370, Wroclaw, Poland

^b^Department of Analytical Chemistry and Chemical Metallurgy, Faculty of Chemistry, Wroclaw University of Science and Technology, Wybrzeze S. Wyspianskiego 27, 50-370, Wroclaw, Poland

^c^Academic Centre for Materials and Nanotechnology, AGH University of Science and Technology, A. Mickiewicza Av. 30, 30-059 Kraków, Poland

^d^Łukasiewicz Research Network - PORT Polish Center for Technology Development, Stablowicka 147, 54-066 Wrocław, Poland

^e^Laboratory of Microscopic Techniques, Faculty of Biological Sciences, University of Wroclaw, H. Sienkiewicza 21, 50-335 Wroclaw, 51-148 Wroclaw, Poland

^f^Faculty of Physics and Applied Computer Science, AGH University of Science and Technology, A. Mickiewicza Av. 30, 30-059 Kraków, Poland

^*^Correspondence to P. Cyganowski; E-mail: piotr.cyganowski@pwr.edu.pl; Phone: +48 71 320 2383

**List of abbreviations**

| NACs | Nitroaromatic compounds |
| --- | --- |
| AAMs | Aromatic amines |
| NPs | Nanoparticles |
| NSs | Nanostructures |
| sub-NSs | sub-nanostructures |
| NB | Nitrobenzene |
| 4-NP | 4-Nitrophenol |
| 2-NA | 2-Nitroaniline |
| 4-NA | 4-Nitroaniline |
| 2,4,6-TNP | 2,4,6-Trinitrophenol |
| NMs | Nanomaterials |
| NCats | Nanocatalysts |
| DNA | deoxyribonucleic acid |
| HRTEM | High-resolution transmission electron microscopy |
| UHR-SEM | Ultra-high-resolution scanning electron microscopy |
| FIB | Focused ion beam |
| EDX | Energy-dispersive X-ray |
| UV/Vis | Ultraviolet/visible light |
| SAED | Selected area electron diffraction |
| HAADF | High-angle annular dark-field |
| XPS | X-ray photoelectron spectroscopy |
| BAPA | Bis(3-aminopropyl)amine |
| CDI | 1,1’-Carbonyldiimidazole |
| HMI | 4(5)-(Hydroxymethyl)imidazole |
| PP | 1-(2-Pyrimidyl)piperazine |
| TSC | Thiosemicarbazide |
| AHP | 2-Amino-3-hydroxypiridine |
| HEP | 1-(2-Hydroxyethyl)piperazine |
| AUr | 4(6)-Aminouracil |
| AT | 2-Aminothiazol |
| ^ext^Re[amine] | Sample obtained using an external reducing agent; |
| Re[amine] | Sample obtained using reduction-coupled adsorption; |
| VBC | Vinylbenzyl chloride |
| DVB | Divinylbenzene |
| PFIB | Plasma FIB |
| STEM | Scanning transmission electron microscopy |
| ICP-OES | Inductively coupled plasma optical emission spectrometry |

**S1.** **Detailed Results and Discussion**

**S1.1. Syntheses of polymer matrices**

As amino functionalities played vital roles in the fabrication and stabilisation of Re nanostructures (ReNSs), the syntheses of polymer matrices were examined by determining whether the introduction of amino functionalities into the vinylbenzyl chloride (VBC)-co-divinylbenzene (DVB) copolymer was successful. This was accomplished via elemental analysis that allowed us to establish the number of functional groups introduced into the VBC-co-DVB copolymer, and the corresponding results were further confirmed by XPS, which will be discussed below. Table 1 presents *Z_N_* and the concentrations of functional groups derived from bis(3-aminopropyl)amine (BAPA), 4(5)-(hydroxymethyl)imidazole (HMI), 1-(2-pyrimidyl)piperazine (PP), thiosemicarbazide (TSC), 2-amino-3-hydroxypiridine (AHP), 1-(2-hydroxyethyl)piperazine (HEP), 4(6)-aminouracil (AUr), 1,1’-carbonyldiimidazole (CDI) and 2-aminothiazol (AT).

**Table S1.** Concentrations of N and functional groups in the developed polymer matrices.

| Polymer matrix | *Z*_N_^1^ | *N*^2^ | *Z*_L_^3^ |
| --- | --- | --- | --- |
| VBC-co-DVB | 0 | 0 | 0 |
| VBC-co-DVB modified with BAPA | 3.63 | 3 | 1.21 |
| VBC-co-DVB modified with CDI | 4.62 | 4 | 1.16 |
| VBC-co-DVB modified with HMI | 3.62 | 1 | 3.62 |
| VBC-co-DVB modified with PP | 7.32 | 4 | 1.83 |
| VBC-co-DVB modified with TSC | 5.20 | 3 | 1.73 |
| VBC-co-DVB modified with AHP | 2.46 | 1 | 2.46 |
| VBC-co-DVB modified with HEP | 4.09 | 2 | 2.04 |
| VBC-co-DVB modified with AUr | 0.26 | 2 | 0.13 |
| VBC-co-DVB modified with AT | 2.56 | 1 | 2.56 |
| ^1^ The concentration of N (mmol g^-1^)  ^2^ The number of N atoms in the functionality  ^3^ The ligand concentration (number of functionalities) (mmol g^-1^) | | | |

The corresponding results verified that the modification of VBC-co-DVB with amino functionalities resulted in the incorporation of N-based moieties into the copolymer. The highest *Z_N_* was attained for the sample modified with PP (7.32 mmol g^-1^) followed by those modified with TSC (5.2 mmol g^-1^), CDI (4.62 mmol g^-1^), HEP (4.09 mmol g^-1^), BAPA (3.63 mmol g^-1^), and HMI (3.62 mmol g^-1^). This order was slightly different for the number of amino ligands (*Z_L_*, calculated as *Z_N_*/*N*, where *N* is the number of N atoms in the functionality) present in each sample. Nevertheless, *Z_L_* in the abovementioned samples ranged from 2.04 to 3.62 mmol g^-1^.

Because ReO_4_^-^ is adsorbed by amino moieties (Fig. 1A and B), the N atoms can further serve as reactive sites for the fabrication of ReNSs (Fig. 1B). Additionally, *Z_N_* is the primary factor enabling the estimation of the efficiency of ReNS production as the higher the *Z_N_*, the higher the adsorption and reduction rate of Re(VII). The data provided in Table 1 indicate that all samples, except for the sample modified with AUr (*Z_N_* = 0.26 mmol g^-1^), should be suitable matrices for nanocatalysts (NCats) with ReNSs. This will be further verified below.

**S1.2 ReNSs in the polymer matrices**

Significant differences between the catalytic activities of different NCat samples suggested that the amounts of ReNSs produced in different polymer matrices were not equal. To confirm this, the concentrations of Re in the NCat, determined by inductively coupled plasma-optical emission spectroscopy (ICP-OES) were evaluated, and the results are provided in Table S2.

**Table S2.** Re concentrations (wt.%) in the NCat samples.

| **Samples containing**  **^ext^Re^1^** | ***C*_Re_^2^** | **Samples**  **containing**  **Re^3^** | ***C*_Re_^2^** |
| --- | --- | --- | --- |
| ^ext^ReBAPA | 5.4 | ReBAPA | 16.7 |
| ^ext^ReCDI | 4.0 | ReCDI | 18.4 |
| ^ext^ReHMI | 3.0 | ReHMI | 15.9 |
| ^ext^RePP | 7.1 | RePP | 17.0 |
| ^ext^ReTSC | 3.7 | ReTSC | 5.4 |
| ^ext^ReAHP | 2.7 | ReAHP | 12.9 |
| ^ext^ReHEP | 5.7 | ReHEP | 16.2 |
| ^ext^ReAUr | 0.0 | ReAUr | 0.0 |
| ^ext^ReAT | 4.6 | ReAT | 12.5 |
| ^1^ Samples obtained using NaBH_4_ as the reducing agent  ^2^ The concentration of Re (wt.%).  ^3^ Samples acquired via reduction-coupled adsorption | | | |

The results presented in Table 1 correspond only to the concentration of Re in the forms of ReNSs, as the unreacted ReO_4_^-^ was desorbed from a polymer before the analysis (see supplementary materials for details). These results indicated that the concentrations of Re in the Re samples were higher than those in the ^ext^Re samples. This explains why the Re samples demonstrated significantly higher catalytic activities than the ^ext^Re samples (Fig. 2 and 3). The concentration of Re in each NCat was expected to be more or less equal. This expectation was supported by the mass balance, implying that each anion-exchange resin removed >98% of Re(VII) from the precursor solution. Nevertheless, despite equal saturation of amino functionalities, the NCats demonstrated different concentrations of Re, ranging from 0 to 7.1% in the cases of the ^ext^Re samples and from 0 to 18.4% in the cases of the Re samples (Table S2). This suggested that among all the applied amines, AUr and TSC evidently prevented or suppressed the reduction of Re(VII) (*C*_Re_ values for these samples were ~0%, Table S2). Additionally, despite relatively high Re concentrations of ^ext^RePP, RePP, ^ext^ReAT, and ReAT (7.1, 17.0, 4.6, and 12.5%, respectively), these NCats exhibited negligible catalytic activities (Fig. 2 and 3); in contrast, other NCats with similar Re concentrations (for example, ^ext^ReHEP and ReHEP, Table S2) demonstrated outstanding catalytic activities. These observations confirm the synergistic effect between ReNSs and amino functionalities and allow us to conclude that the applied amine precisely regulates the synthesis routes of ReNSs irrespective of the method used.

*Electron microscopy imaging*

Based on the ultra-high resolution scanning electron microscopy (UHR-SEM), the following detailed conclusions can be drawn. The synthetic approach affected the distribution of Re in NCat. Application of NaBH_4_ to reduce ReO_4_^-^ resulted in the agglomeration of large particles, and the agglomerates were mainly located near the surface of the polymer grain. In contrast, reduction-coupled adsorption generated ReNSs over the entire volume of the polymer grain, and the fabricated ReNSs were significantly smaller and better dispersed than those synthesised using NaBH_4_. Finally, the amino functionality might influence both locations and dispersions of ReNSs in the polymer matrix regardless of the applied synthetic method.

All these conclusions were supported by the observed concentrations of Re (Table 1) and the catalytic activities (Fig. 2 and 3) of the NCats. The concentrations of Re in the Re samples were higher because the ReNSs were produced over the whole volume of the polymer grain; in contrast, Re in the ^ext^Re samples was primarily situated near the surface of the polymer grain. This explained the higher catalytic activities of the Re samples (Fig. 3) than those of the ^ext^Re samples (Fig. 2). Moreover, the primary role of the applied amine was outlined. The samples (including ^ext^RePP, ^ext^ReTSc, ^ext^ReAT, RePP, ReAHP, and ReAT) characterised by the lowest catalytic activities despite considerable Re concentrations had numerous agglomerates of ReNSs on the polymer grain surfaces, rendering them ineffective in the catalytic process. This further verified the synergistic effect between amino functionalities and the ReNSs formed on them.

The very small sizes of these groups indirectly explain the insufficiency of high-resolution transmission electron microscopy (HRTEM) for the analyses of NCat samples. In the case of HRTEM, the phase contrast can be observed as the effect of electron diffraction in the crystalline structure of the observed object. In contrast, scanning-transmission electron microscopy (STEM) conducted using high-angle annular dark field detector (HAADF) provides amplitude contrast as a sign of scattering. This suggested that although no “volumetric” structures (nanoparticles) were noticed in ReBAPA, the corresponding images might have been acquired at the very beginning of the formation of these structures, which would have been “frozen” by the polymer matrix itself. This was ascribed to the effective stabilisation of ReO_4_^-^ by amines, which might have prevented the growth of reduced Re forms.

HRTEM-STEM-HAADF results demonstrated the differences between the abovementioned subnanometric Re groups in the investigated samples. In the case of ReBAPA, these groups were noticed in the whole volume of the polymer grain, covering almost the entire cross-section. For ^ext^ReBAPA, the densities of these groups were lower than those in the case of ReBAPA (Fig. 6B, right panel), and occasionally, NSs were indeed produced (Fig. 6B, left panel). In this case, areas with no Re atom groups were observed. Finally, ^ext^RePP contained a considerable number of NSs (Fig. 5C and 6C) and Re atom groups (Fig. 6C, right panel). Nevertheless, in ^ext^RePP, the Re atoms grouped into larger structures as compared to those in ReBAPA and ^ext^ReBAPA, and the areas where they were located were rarely observed. These findings were consistent with those of the EDX maps acquired using UHR-SEM (Fig. 4). However, most importantly, there was a direct association between the formation of Re-sub-NSs and the catalytic activities of the developed NCats. In this regard, *k* values of 4-NP reduction followed the order ReBAPA > ^ext^ReBAPA >> ^ext^RePP (Fig. 2 and 3). According to these findings, it was concluded that although BAPA prevented the formation of NSs, it facilitated the production of Re-sub-NSs, which were catalytically active and substantially boosted the catalytic activities of catalysts in the reduction of 4-NP. This conclusion might be extended to other samples that exhibited higher catalytic activities than those of other samples. Thus, CDI, HMI, and HEP functionalities must also contribute to the formation of Re-sub-NSs.

**S1.3 Bonds and Re oxidation states in catalysts samples**

**Table S3.** Surface compositions (atomic %) determined by fitting XPS spectra for all analysed samples.

|  | **C** | | | **N** | | **O** | | | **Si** | | **Cl** | | **S** | | **Re** | | |
| --- | --- | --- | --- | --- | --- | --- | --- | --- | --- | --- | --- | --- | --- | --- | --- | --- | --- |
| Binding energy [eV] | 285.0 | 286.6 | 288.3 | 400.2 | 402.0 | 531.7 | 532.8 | 533.9 | 101.8 | 103.2 | 198.3 | 200.1 | 162.5 | 164.0 | 42.4 | 44.0 | 46.1 |
| Sample / Bonds | C-C | C-O  C-S  C-Cl  C-N | O-C=O  C=O | C-NH | NH_4_^+^ | O-Re  O-Si | O-C  O=C | -OH  H_2_O_ads_ | silicon  siloxane | SiO_2_ | Cl^-^ | Cl-C | S^2-^ | C-S | Re^4+^ | Re^6+^ | Re^7+^ |
| ^ext^ReBAPA | 34.5 | 23.4 | 7.5 | 3.8 | 1.0 | 6.8 | 14.1 | 4.5 | 2.4 | 2.1 | 0.0 | 0.0 | 0.0 | 0.0 | 0.01 | 0.02 | 0.01 |
| ^ext^ReCDI | 39.3 | 21.6 | 5.5 | 2.6 | 1.1 | 5.9 | 13.1 | 6.4 | 2.3 | 2.1 | 0.0 | 0.0 | 0.0 | 0.0 | 0.00 | 0.01 | 0.05 |
| ^ext^ReHMI | 37.5 | 19.2 | 7.0 | 4.7 | 0.5 | 6.1 | 15.3 | 4.2 | 3.3 | 2.2 | 0.0 | 0.0 | 0.0 | 0.0 | 0.01 | 0.01 | 0.02 |
| ^ext^RePP | 47.2 | 17.7 | 2.2 | 2.2 | 1.2 | 4.3 | 12.2 | 6.0 | 3.4 | 3.3 | 0.0 | 0.0 | 0.0 | 0.0 | 0.00 | 0.14 | 0.15 |
| ^ext^ReTSC | 43.1 | 18.1 | 5.0 | 2.2 | 0.9 | 3.7 | 15.8 | 3.7 | 2.6 | 3.0 | 0.1 | 0.4 | 0.3 | 0.9 | 0.14 | 0.06 | 0.12 |
| ^ext^ReAHP | 50.2 | 15.4 | 4.1 | 1.5 | 0.5 | 3.8 | 16.6 | 2.6 | 2.7 | 2.4 | 0.2 | 0.1 | 0.0 | 0.0 | 0.01 | 0.04 | 0.07 |
| ^ext^ReHEP | 43.5 | 18.3 | 4.3 | 2.7 | 0.7 | 3.4 | 19.8 | 1.7 | 2.4 | 2.9 | 0.2 | 0.0 | 0.0 | 0.0 | 0.02 | 0.08 | 0.05 |
| ^ext^ReAUr | 48.5 | 17.9 | 3.2 | 0.8 | 0.0 | 1.9 | 18.2 | 3.4 | 2.1 | 3.0 | 0.2 | 0.7 | 0.0 | 0.0 | 0.00 | 0.01 | 0.01 |
| ^ext^ReAT | 41.6 | 17.3 | 5.9 | 2.0 | 0.9 | 6.0 | 15.8 | 3.5 | 2.4 | 3.4 | 0.3 | 0.3 | 0.2 | 0.3 | 0.03 | 0.05 | 0.13 |
| ReBAPA | 37.8 | 21.9 | 7.7 | 2.3 | 0.7 | 4.4 | 14.3 | 5.3 | 2.2 | 2.7 | 0.1 | 0.2 | 0.0 | 0.0 | 0.04 | 0.13 | 0.23 |
| ReCDI | 45.3 | 17.6 | 5.5 | 3.1 | 1.4 | 4.5 | 13.3 | 4.2 | 2.5 | 2.3 | 0.0 | 0.0 | 0.0 | 0.0 | 0.01 | 0.11 | 0.29 |
| ReHMI | 50.3 | 15.9 | 3.7 | 1.6 | 0.7 | 2.8 | 16.3 | 3.5 | 3.2 | 1.8 | 0.0 | 0.0 | 0.0 | 0.0 | 0.01 | 0.07 | 0.19 |
| RePP | 40.8 | 18.6 | 5.1 | 2.6 | 0.8 | 8.2 | 11.8 | 4.4 | 1.3 | 5.7 | 0.0 | 0.0 | 0.0 | 0.0 | 0.00 | 0.30 | 0.43 |
| ReTSC | 44.4 | 17.3 | 4.3 | 3.1 | 0.7 | 6.1 | 14.2 | 3.6 | 2.2 | 2.9 | 0.0 | 0.0 | 0.0 | 1.0 | 0.01 | 0.05 | 0.15 |
| ReAHP | 41.8 | 19.0 | 5.6 | 3.1 | 0.5 | 7.1 | 14.2 | 3.2 | 1.8 | 3.0 | 0.2 | 0.0 | 0.0 | 0.0 | 0.06 | 0.11 | 0.26 |
| ReHEP | 47.9 | 18.5 | 3.2 | 2.7 | 0.6 | 4.4 | 14.7 | 3.6 | 1.1 | 3.0 | 0.0 | 0.0 | 0.0 | 0.0 | 0.01 | 0.18 | 0.26 |
| ReAUr | 45.3 | 17.9 | 4.6 | 1.8 | 0.5 | 4.3 | 15.5 | 4.0 | 2.2 | 2.7 | 0.2 | 0.9 | 0.0 | 0.0 | 0.00 | 0.00 | 0.00 |
| ReAT | 43.8 | 19.8 | 4.7 | 1.8 | 0.7 | 6.2 | 14.5 | 3.6 | 2.1 | 2.4 | 0.0 | 0.0 | 0.0 | 0.0 | 0.04 | 0.06 | 0.25 |

**S1.4 Catalytic reductions of nitroaromatics (NACs) over ReBAPA catalyst**

The catalytic reductions of nitrobenzene (NB), 4-nitrophenol (4-NP), 2-nitroaniline (2-NA), 4-nitroaniline (4-NA), and 2,4,6-trinitrophenol (2,4,6-TNP) were carried out. Maximum conversions of NACs ranged between 83% (for 2-NA reduction) and 97% (for 4-NP and 4-NA reductions) (Fig. S1A). Interestingly, ReBAPA led to maximum or almost maximum NAC conversions within just 20 min of the start of the process (Fig. S1A). This might suggest similar catalytic activity of ReBAPA towards each NAC. The actual reason for the different *k_1_* values of different reductions was probably the molar activity of the catalyst, expressed as *TOF* (Fig. S1B). The *TOF* values calculated for 70 and 80% reductions were similar for each catalytic process and proportionally decreased as the maximum reduction approached. Interestingly, the *TOF* values evaluated at the beginning of the process (that is, at 20% NAC conversions) were significantly different from those measured thereafter (Fig. S1B). Although ReBAPA exhibited the highest *k_1_* values for the catalytic reductions of 4-NP (0.21 min^-1^) and 4-NA (0.18 min^-1^), the highest activity of ReBAPA at the start of the process was observed for the reduction of NB. In this case, the *TOF* value for 20% NB conversion was over two times those for other NACs (Fig. S1B). As this effect was noticed only at the beginning of the process (initial 1–3 min), it may be associated with the mechanism of hydrogenation. The Re-based catalytic centres are obstructed within the grains of the cross-linked suspension copolymer. Therefore, each NAC must be adsorbed by the polymer matrix before its actual reduction. Owing to considerable differences between the *TOF* values obtained at the beginning of the process and those evaluated thereafter, it can be deduced that the rate-limiting step at the beginning of the process may indeed be diffusion limitations that the substrates experience. These results may be further discussed in terms of the structural morphology of each NAC. NB demonstrates the simplest structure among those of all the tested NACs. It consists of a benzene ring and a ‒NO_2_ group, and no other substituents are present (Fig. 7). Thus, the diffusion of NB in the polymer matrix is the easiest. Next, the structures of 4-NP and 4-NA are similar to each other, characterised by a single ‒NO_2_ group separately attached to a hydroxy- and amino-substituted benzene ring. In these cases, the *TOF* values were almost the same (Fig. S1B). The lowest activities of ReBAPA were observed for 2,4,6-TNP and 2-NA reductions. Considering the abovementioned context, diffusion limitations in the case of 2,4,6-TNP must be the highest because the structure of 2,4,6-TNP is the most complex. Furthermore, *ortho*-substituted amines, such as 2-NA, experience steric hindrances [1], which may be the reason for difficulties in accessing active centres in these cases. The higher *TOF* values for 50% 2-NA and 2,4,6-TNP conversions and, thus, the similar activities of ReBAPA for 2-NA and 2,4,6-TNP reductions as that for NB reduction further support the conclusion related to NAC diffusion limitations. Based on all these observations, it can be concluded that ReBAPA is a universal catalyst for the reduction of NACs.

[1] V.V. Patil, G.S. Shankarling, Steric-Hindrance-Induced Regio- and Chemoselective Oxidation of Aromatic Amines, J. Org. Chem. 80 (2015) 7876–7883. https://doi.org/10.1021/acs.joc.5b00582.

**
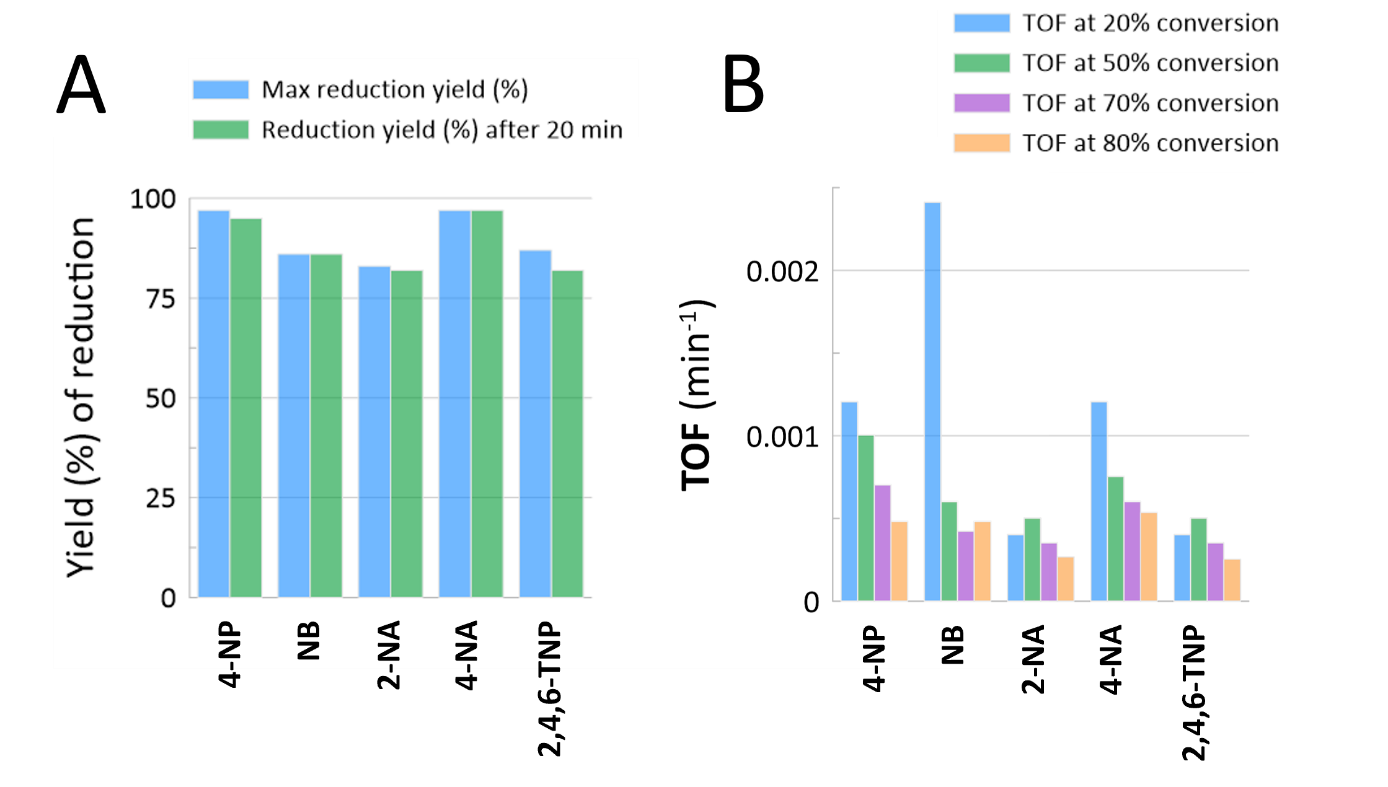

Fig. S1.** Parameters for NAC reductions over ReBAPA. (**A**) NAC reduction yields (%) and (**B**) turnover frequency (*TOF*) values for each catalytic reaction.

**Table S4.** Rate constants, TOF parameters and yield of conversions for catalytic reductions of NACs

| **Stabilising amine^1^** | **Sample^2^** | **Substrate^3^** | ***k_1_^4^*** *×10^-3^* | ***TOF^5^*** *×10^-3^* | **Maximum conversion**  (%) | **Sample^2^** | **Substrate^3^** | ***k_1_^4^****×10^-3^* | ***TOF^5^****×10^-3^* | **Maximum conversion**  (%) |
| --- | --- | --- | --- | --- | --- | --- | --- | --- | --- | --- |
| BAPA | Re | NB  2-NA  4-NA  2,4,6-TNP | 130  100  180  90 | 0.26  0.20  0.29  0.1 | 86  83  97  87 | ^ext^Re |  |  |  |  |
|  |  | 4-NP | 210 | 0.29 | 97 |  | 4-NP | 1.78 | 0.0028 | 41 |
| CDI |  |  | 64 | 0.096 | 96 |  |  | 2.33 | 0.20 | 95 |
| HMI |  |  | 77 | 0.13 | 97 |  |  | 16.4 | 0.18 | 93 |
| PP |  |  | 0.46 | 0.002 | 16 |  |  | 0.048 | ~0 | 2 |
| TSC |  |  | 0 | 0 | 0 |  |  | 0.057 | ~0 | 2 |
| AHP |  |  | 0.61 | 0.007 | 32 |  |  | 0.90 | 0.003 | 27 |
| HEP |  |  | 41 | 0.016 | 84 |  |  | 32.5 | 0.05 | 95 |
| AUr |  |  | 0 | 0 | 0 |  |  | 0.096 | ~0 | 3 |
| AT |  |  | 0.72 | 0.005 | 20 |  |  | 0.049 | ~0 | 3 |
| ^1^ BAPA: bis(3-aminopropyl)amine; CDI: 1,1’-carbonyldiimidazole; HMI: 4(5)-(hydroxymethyl)imidazole; PP: 1-(2-pyrimidyl)piperazine; TSC: thiosemicarbazide; AHP: 2-amino-3-hydroxypiridine; HEP: 1-(2-hydroxyethyl)piperazine; AUr: 4(6)-aminouracil; AT: 2-aminothiazol  ^2^ Samples obtained using (^ext^Re) NaBH_4_ as the reducing agent or (Re) reduction-coupled adsorption  ^3^ 4-NP: 4-nitrophenol; NB: nitrobenzene; 2-NA: 2-nitroaniline; 4-NA: 4-nitroaniline, 2,4,6-TNP: 2,4,6-trinitrophenol (initial concentration 0.1 mmol L^-1^).  ^4^ Pseudo first order rate constant (min^-1^)  ^5^ Turnover frequency (min^-1^); calculated at maximum conversion | | | | | | | | | | |

**S2.** **Detailed Methods and Materials**

**2.1 Materials**

Reagents, *namely*, vinylbenzyl chloride (VBC), divinylbenzene (DVB), benzoyl peroxide, poly(vinyl alcohol), bis(3-aminopropyl)amine (BAPA), 4(5)-(hydroxymethyl)imidazole (HMI), 1-(2-pyrimidyl)piperazine (PP), thiosemicarbazide (TSC), 2-amino-3-hydroxypiridine (AHP), 1-(2-hydroxyethyl)piperazine (HEP), 4(6)-aminouracil (AUr), 1,1’-carbonyldiimidazole (CDI) and 2-aminothiazol (AT), used for the preparation of anion-exchange resins were acquired from Merck (Poland). Ammonium perrhenate (NH_4_ReO_4_) was purchased from Merck (Poland). Selected NACs, that is, NB, 4-NP, 2-NA, 4-NA, and 2,4,6-TNP, and NaBH_4_ (analytical grade), for catalytic studies were also procured from Merck (Poland). All other reagents listed in this manuscript were acquired from Avantor Performance Materials Ltd. (Gliwice, Poland). All reagents were of analytical grade or higher and were used as received.

**2.2 Instruments and analyses**

The concentration of N (*Z_N_*) derived from amino functionalities was determined by elemental analysis using a Vario (Elementar Analysensysteme GmbH, Germany) automatic analyzer. Morphologies of the polymer samples were assessed by ultra-high-resolution scanning electron microscopy (UHR-SEM) using FEI Helios NanoLab™ 600i and FEI Helios plasma focused ion beam (PFIB) (OR, USA) equipped with Ga-focused ion beam (Ga-FIB) and Xe-PFIB and energy-dispersive X-ray (EDX) spectrometers. Moreover, ReNSs loaded onto different polymers were characterised by high-resolution transmission electron microscopy (HRTEM) using FEI TITAN^3^ equipped with a selected area electron diffractometer, EDX spectrometer, and high-angle annular dark-field (HAADF) detector in the scanning transmission electron microscopy (STEM) mode. The samples were prepared by embedding the polymers in Epon 812 resin (Serva, Germany). Thereafter, the synthesised materials were sectioned using a Reichert-Jung Ultracut E (Leica) ultramicrotome, and 100 nm slices were placed on Ni Formvar/C grids (100 mesh, Agar Scientific Ltd., Stansted, Great Britain). Concentrations of Re in the NCat samples were determined via inductively coupled plasma optical emission spectrometry (ICP-OES) using an Agilent 5110 instrument. The samples were mineralised in concentrated HNO_3_ at 170–190 °C using an SCP Science DigiPREP Jr. digestion block and quartz flat-bottom digestion tubes (100 mL) with appropriate watch glasses or a microwave digestion system (Anton Paar Multiwave PRO). Before analysis, the ReNS-loaded polymer samples were washed with a 1% NH_4_OH solution to remove unreduced ReO_4_^-^. ICP-OES was also employed to evaluate the equilibrium concentration of Re(VII) after the uptake of Re(VII) by anion-exchange resins.

X-ray photoelectron spectroscopy (XPS) was conducted by a PHI VersaProbeII Scanning XPS system using monochromatic Al Kα (1486.6 eV) X-rays focused to a 100 µm spot and scanned over an area of 400 × 400 µm^2^. The photoelectron take-off angle was 45°, and the pass energy in the analyser was set to 117.50 eV (step size: 0.5 eV) for survey scans and 46.95 eV (step size: 0.1 eV) to obtain high-energy resolution spectra for C 1s, O 1s, Si 2p, N 1s, S 2p, Cl 2p, and Re 4f regions. Dual beam charge compensation with 7 eV Ar^+^ and 1 eV electrons was utilised to maintain a constant sample surface potential regardless of the sample conductivity. All XPS spectra were charge-referenced to the unfunctionalized C-C C 1s peak at 285.0 eV. The operating pressure in the analytical chamber was less than 3 × 10^-9^ mbar. Deconvolution of the spectra was performed using PHI MultiPak (v. 9.9.3). Spectrum background was subtracted using the Shirley method. Samples were prepared by successive drop-casting and evaporation of the solvent (H_2_O) from the polymeric particles deposited on a Si wafer substrate.

The catalytic reaction rate was monitored by ultraviolet/visible (UV/Vis) spectrophotometry using SPECORD 210 PLUS (Analytik-Jena, Jena, Germany). The corresponding spectra were acquired in the range of 200–700 nm with a resolution of 1 nm using a quartz cuvette.

**2.3 Syntheses of Re-loaded NCats**

At first, the polymer matrix, namely, VBC-co-DVB (copolymer), was fabricated by suspension polymerisation as reported in a previous study [1]. Then, the resultant copolymer VBC-co-DVB was separately modified by selected amines, that is, BAPA, 1,1’-carbonyldiimidazole (CDI), HMI, PP, TSC, AHP, HEP, AUr, and AT, using a previously reported method [1]. Subsequently, the resulting anion-exchange resins (0.1 g) were mixed with solutions (50 mL, 500 mg Re L^-1^) containing NH_4_ReO_4_ in 0.1 mol L^-1^ HCl. This led to anion exchange between amino functionalities and ReO_4_^-^. Thereafter, two approaches were used to synthesise and stabilise ReNSs in the polymer matrix (Fig. 1).

The first approach involved the reduction of ReO_4_^-^ using NaBH_4_ as a reducing agent. Typically, the Re(VII)-loaded anion-exchange resins were separated from the precursor solution by filtration and washed with water on a fritted-glass funnel followed by introduction into 50 mL water comprising 0.1 mol L^-1^ NaBH_4_. This resulted in the immediate syntheses and precipitations of ReNSs in the polymer matrix. After 24 h, each resin was separated by filtration, washed with water, and used in its swollen state. The second approach included the reduction of ReO_4_^-^ *via* the transfer of an electron from the N atom of the amino functionality to ReO_4_^-^ (Fig. 1B). Subsequently, the Re(VII)-loaded anion-exchange resins were separated from the precursor solution, washed with water, and stored in 50 mL water for 4 weeks. This caused gradual reduction and precipitation of ReNSs. Although both methods involved *in situ* reduction, the second method provided a stable concentration gradient between the reactive groups (amino functionalities) and ReO_4_^-^ at the solid/liquid interface without any external reducing agent; thus, the resulting ReNSs were expected to be smaller and appropriately dispersed in the polymer matrix as compared to the cases of nanomaterials (NMs) achieved using NaBH_4_.

NCat samples were named according to the abbreviations of the amines present in the prepared polymer matrices, and the prefixes ^ext^Re and Re were used to represent ReNSs obtained using an external reducing agent (for example, ^ext^ReBAPA) and reduction-coupled adsorption (for instance, ReBAPA), respectively.

**2.4 Investigation of the catalytic activities of the NCats containing ReNSs**

To verify the catalytic activities of the NCats containing ReNSs, the reduction of 4-NP to 4-AP was analysed as the model reduction process for each sample. The reaction rates were determined using the corresponding UV/Vis spectra by calculating the maximum absorbance of 4-NP at a specific wavelength (λ_max_). Initially, 2.5 mL 4-NP solution (0.1 mmol L^-1^) was added to a 3 mL quartz cuvette, and the absorbance of 4-NP at λ_max_ = 318 nm was measured. Subsequently, 0.3 mL NaBH_4_ solution (0.1 mol L^-1^) was introduced into the 4-NP solution. This shifted the λ_max_ of 4-NP towards higher wavelengths because of the formation of 4-nitrophenolate (Ar‒O^-^), and the maximum absorbance was acquired at λ_max_= 400 nm. Thereafter, 0.05 g of a certain NCat was added to the as-prepared mixture, gradually decreasing the absorbance of the absorption band at λ_max_= 400 nm and leading to the appearance of a new band at 295 nm, which was assigned to 4-AP. This process was monitored until the maximum conversion of 4-NP was achieved (evidenced by the lowest absorbance at λ_max_= 400 nm). Due to the large excess of NaBH_4_, the reduction kinetics were assessed using the pseudo-first-order kinetic model, speculating that λ_max_ was proportional to the change in the concentration of 4-NP. Consequently, –ln(*A_t_*/*A_0_*) versus *t* plots were constructed (where *A_t_* is the absorbance at time *t* and *A_0_* is the absorbance at the beginning of the process), and rate constants (*k* (min^-1^)) were evaluated from the slopes of these plots [2,3]. Next, the *k* values were recalculated to obtain the mass-normalised rate constants *k_m_* (g^-1^ min^-1^). Finally, the mass-dependent activity of each catalyst was estimated using turnover frequency (*TOF*, min^-1^), defined as *n_4-NP_* × *r* × *n_Re_*^-1^ × t^-1^, where *n_4-NP_* and *n_Re_* represent the number of moles of 4-NP and ReNSs at the start of the process, respectively, *t* is the process time (min), and *r* is the yield (%) of reduction at which *TOF* is calculated.

After assessing the catalytic activities of the developed NCats in the model kinetic reaction, these NCats were utilised to reduce other NACs, that is, NB, 2-NA, 4-NA, and 2,4,6-TNP, using single-component solutions and the same reaction conditions as those employed for 4-NP reduction. The reaction progress was monitored by observing changes in the absorbances of NB, 2-NA, 4-NA, and 2,4,6-TNP at λ_max_ = 275, 410, 380, and 390 nm, respectively, and the corresponding results were further used to calculate *k*, *k_m_*, and *TOF*.

[1] P. Cyganowski, A. Dzimitrowicz, Heterogenous nanocomposite catalysts with rhenium nanostructures for the catalytic reduction of 4-nitrophenol, Sci. Rep. 12 (2021) 6228. https://doi.org/10.1038/s41598-022-10237-5.

[2] Y. Lu, Y. Mei, M. Drechsler, M. Ballauff, Thermosensitive Core–Shell Particles as Carriers for Ag Nanoparticles: Modulating the Catalytic Activity by a Phase Transition in Networks, Angew. Chem. Int. Ed. 45 (2006) 813–816. https://doi.org/10.1002/anie.200502731.

[3] Y. Wang, Q. Li, P. Zhang, D. O'Connor, R.S. Varma, M. Yu, D. Hou, One-pot green synthesis of bimetallic hollow palladium-platinum nanotubes for enhanced catalytic reduction of p-nitrophenol, J. Colloid Interface Sci. 539 (2019) 161–167. https://doi.org/10.1016/j.jcis.2018.12.053.

**S2.5 XPS spectra acquired for the investigated samples**

The N 1s spectra were fitted with two lines, first centered at 400.2 eV which indicate presence of either C-NH and/or N-(C=O) type bonds in organic compounds, which is attributed to the presence of amino functionalities in the structures of the analyzed samples, and thus, may indicate on the successful modification of VBC-co-DVB copolymers. Second line in the N 1s spectra was found at 402.0 eV indicating presence of NH_4_^+^ ions [1], that may originate from residual desorbing agent applied for the removal of ReO_4_^-^ (the atomic % concentration was ~1, Table 3). The Cl 2p spectra were fitted with two doublet structures (doublet separation p_3/2_ – p_1/2_ equals 1.6 eV) with first 2p_3/2_ line centered at 198.3 eV which indicate presence of Cl^-^ ions in chlorides and second 2p_3/2_ line positioned at 200.1 eV indicating presence of organic type C-Cl bonds [1-4]. The first group (ionic Cl) originated from protonated amines, as the immobilization of ReO_4_^-^ oxyanion was carried out in HCl. The second group (covalently-bonded Cl) is attributed to the unreacted ‒CH_2_Cl groups remaining in the VBC-co-DVB copolymer.

The S 2p spectra were fitted with up to two doublet structures (doublet separation p_3/2_ – p_1/2_ equals 1.18 eV) with first 2p_3/2_ line centered at 162.5 eV which indicate presence of S^2-^ ions in sulfides and second 2p_3/2_ line positioned at 164.0 eV indicating presence of organic type C-S bonds like in thiols [3, 5, 6]. These were detected only in the samples modified using TSC and AT (Table 3, Section S1 in the supplementary information). The O 1s spectra were fitted with three components: first line centered at 531.7 eV which points out the existence of oxygen in metal oxides (Re-O, Si-O), second line centered at 532.8 indicate presence of O-C and/or O=C type bonds and third line at 533.9 eV indicate –OH type compounds and/or adsorbed water [2]. The C 1s spectra can be fitted with three components: first line at 285.0 eV indicate presence of aliphatic carbon, second line either C-O or C-N or C-Cl or C-S groups all laying at the same binding energy (286.6 eV) and C=O and/or O-C=O groups evidenced with the line centered at 288.3 eV [3]. All of these can come from either, the polymeric matrices (see Figure 1 for details) and ReNSs as well as ReASSs present in the synthesized catalysts.

The Re 4f spectra were fitted with up to three doublet structures (doublet separation f_7/2_ – f_5/2_ equals 2.43 eV) with first 4f_7/2_ line centered at 42.4 eV which indicate Re^4+^ oxidation state like in ReO_2_, second 4f_7/2_ line centered at 44.0 eV points out the existence of Re^6+^ oxidation state like in ReO_3_ and last 4f_7/2_ line found at 46.1 eV indicate Re^7+^ oxidation state like in Re_2_O_7_ [2, 7]. Based on these results, it can be stated that the reduction of ReO_4_^-^ was indeed successful. Also, it must be noticed, that Re^7+^ in Re_2_O_7_ could be differed from the Re^7+^ in NH_4_ReO_4_ (precursor).

[1] D. Briggs, Surface Analysis of Polymers by XPS and Static SIMS, Cambridge University Press, New York, 2005.

[2] A.D. Wagner, A.V. Naumkin, A. Kraut-Vass, J.W. Allison, C.J. Powell, J.R.J. Rumble, NIST Standard Reference Database 20, http:/srdata.nist.gov/xps/, 2003.

[3] High Resolution XPS of Organic Polymers: The Scienta ESCA300 Database (Beamson, G.; Briggs, D.), Journal of Chemical Education 70(1) (1993) A25.

[4] P. Rouxhet, M. Genet, XPS analysis of bio-organic systems, Surface and Interface Analysis 43(12) (2011) 1453-1470.

[5] M. Fantauzzi, B. Elsener, D. Atzei, A. Rigoldi, A. Rossi, Exploiting XPS for the identification of sulfides and polysulfides, RSC Advances 5(93) (2015) 75953-75963.

[6] T. Ishida, N. Choi, W. Mizutani, H. Tokumoto, I. Kojima, H. Azehara, H. Hokari, U. Akiba, M. Fujihira, High-resolution X-ray photoelectron spectra of organosulfur monolayers on Au(111): S(2p) spectral dependence on molecular species, Langmuir 15(20) (1999) 6799-6806.

[7] J. Okal, W. Tylus, L. Kȩpiński, XPS study of oxidation of rhenium metal on γ-Al2O3 support, 225(2) (2004) 498-509.

**S3. XPS spectral data**

**S3.1. XPS Survey scans**

**
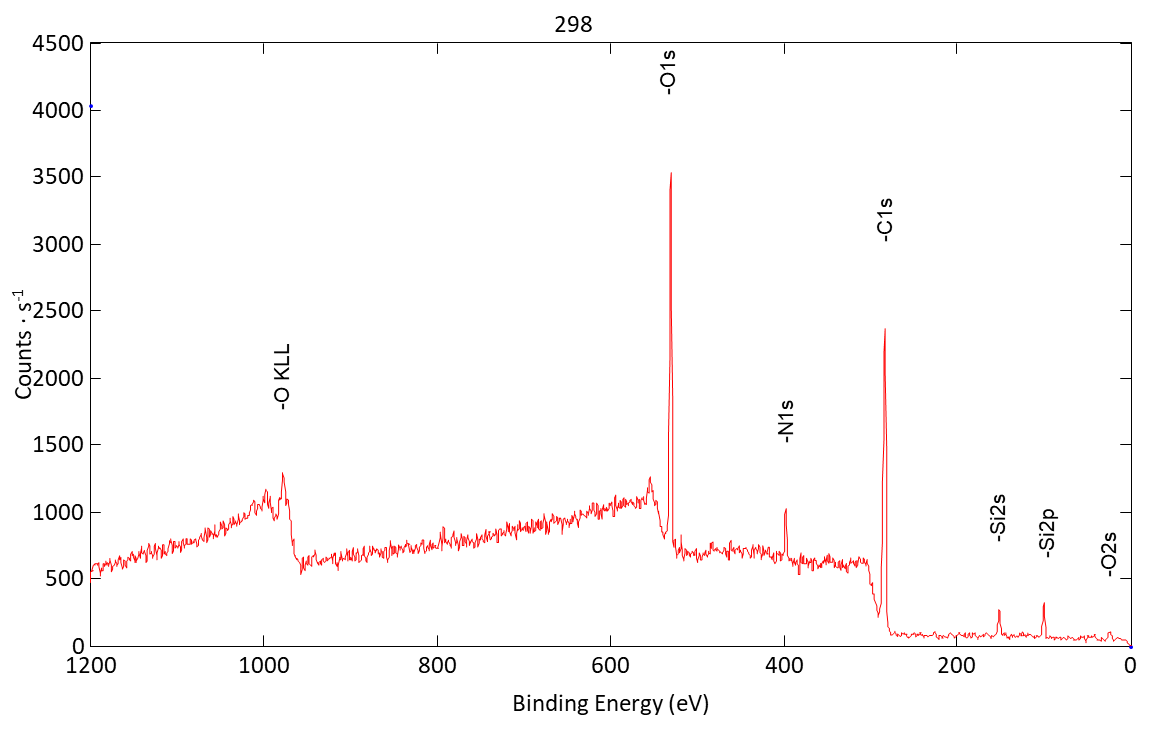
**

**Figure S2.** XPS survey scan of the ^ext^ReBAPA sample

**
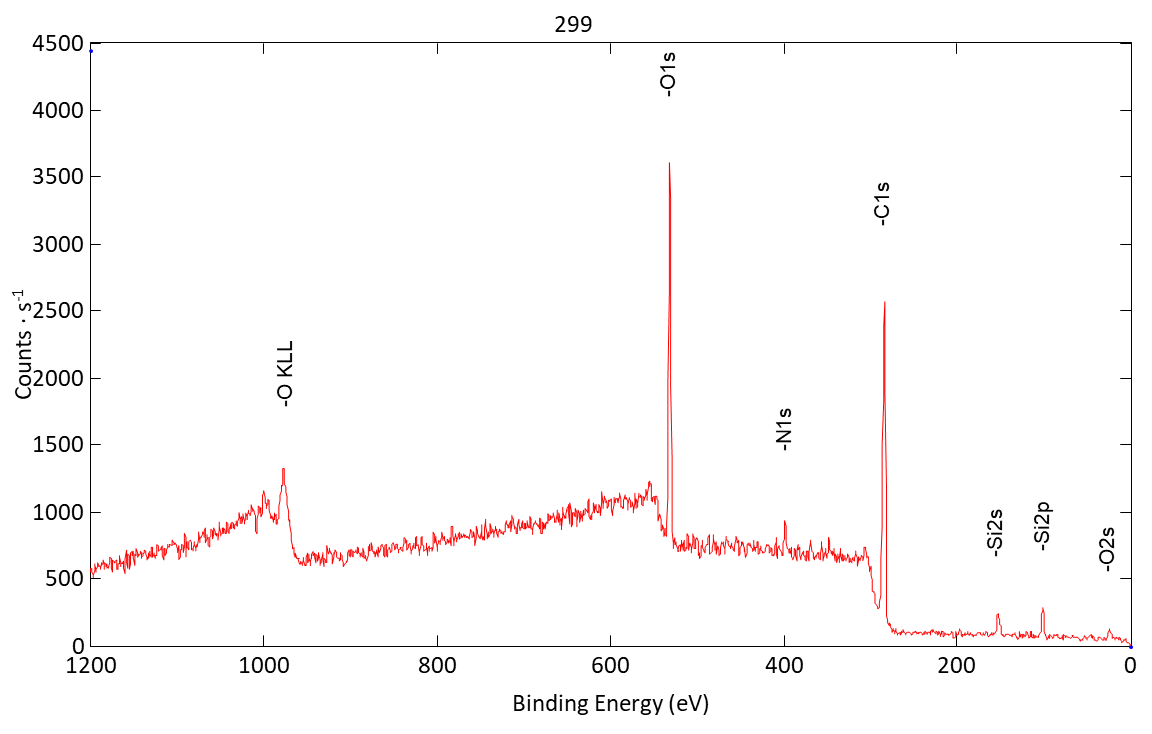
**

**Figure S3.** XPS survey scan of the ^ext^ReCDI sample

**
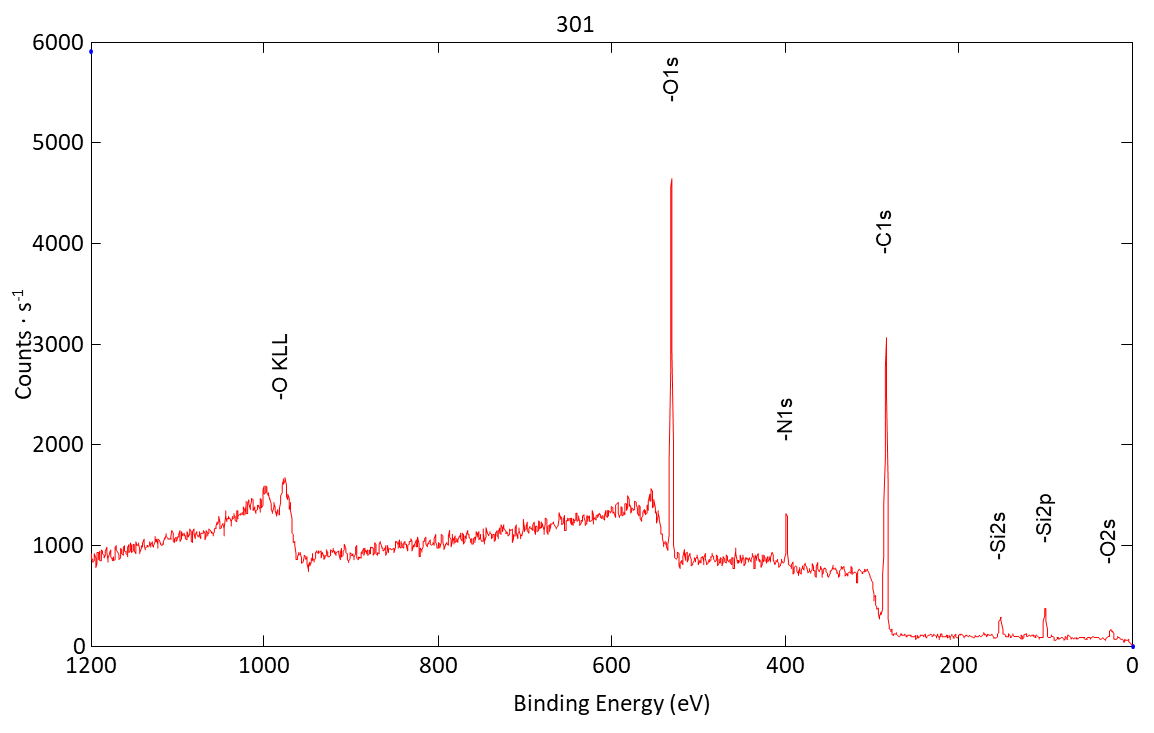
**

**Figure S4.** XPS survey scan of the ^ext^ReHMI sample

**
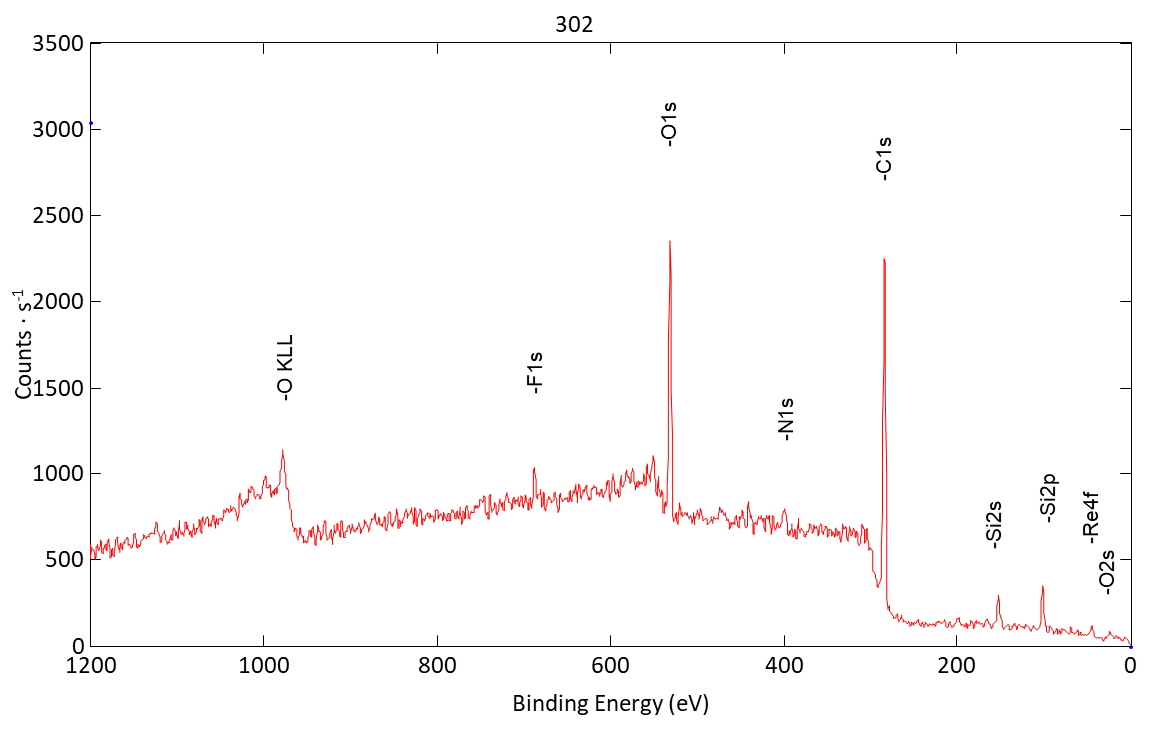
**

**Figure S5.** XPS survey scan of the ^ext^RePP sample

**
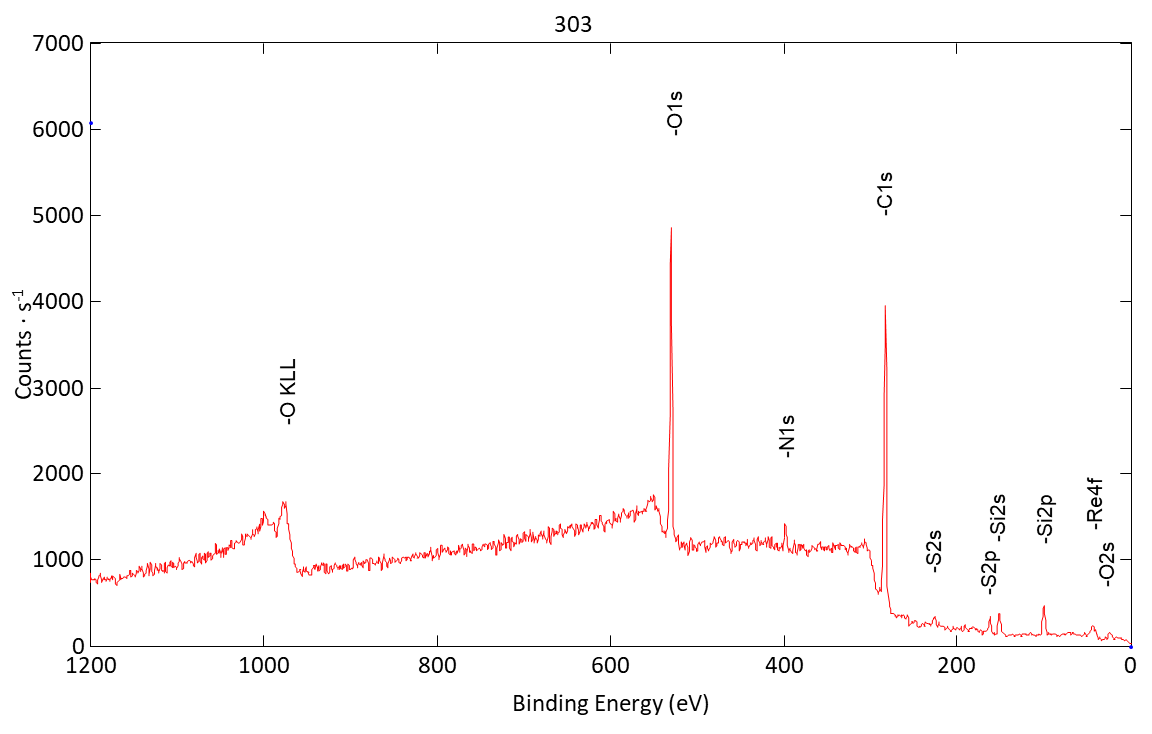
**

**Figure S6.** XPS survey scan of the ^ext^ReTSC sample

**
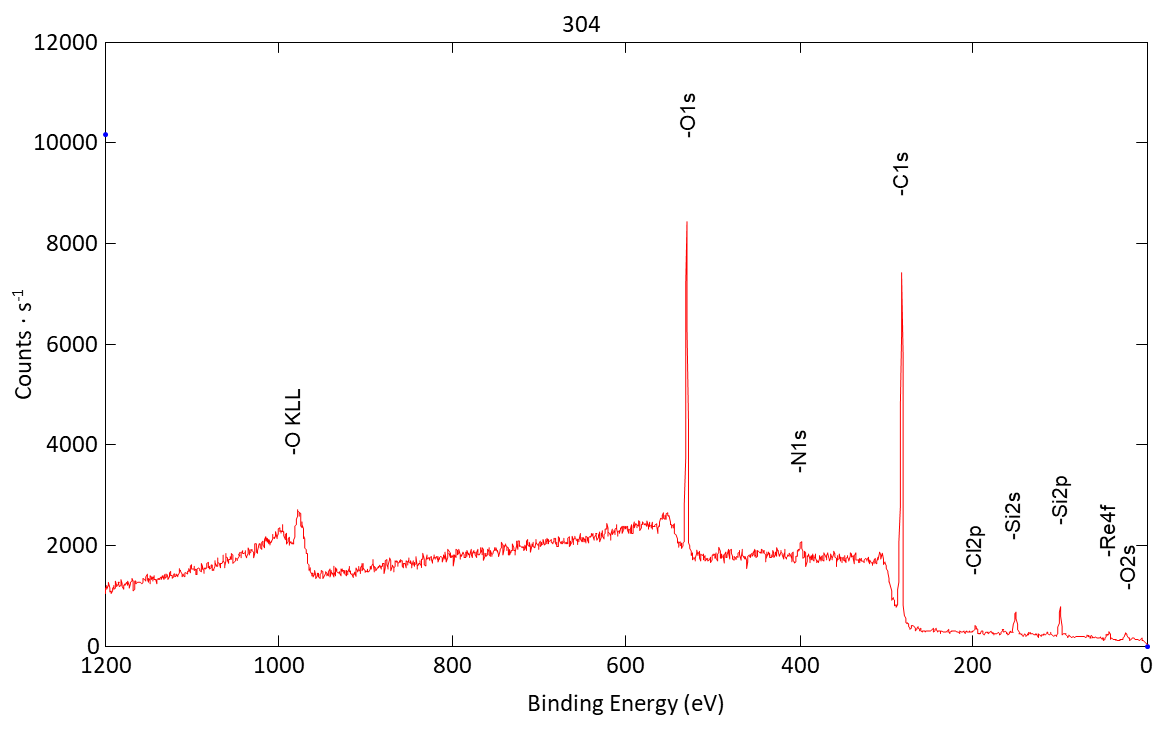
**

**Figure S7.** XPS survey scan of the ^ext^ReAHP sample

**
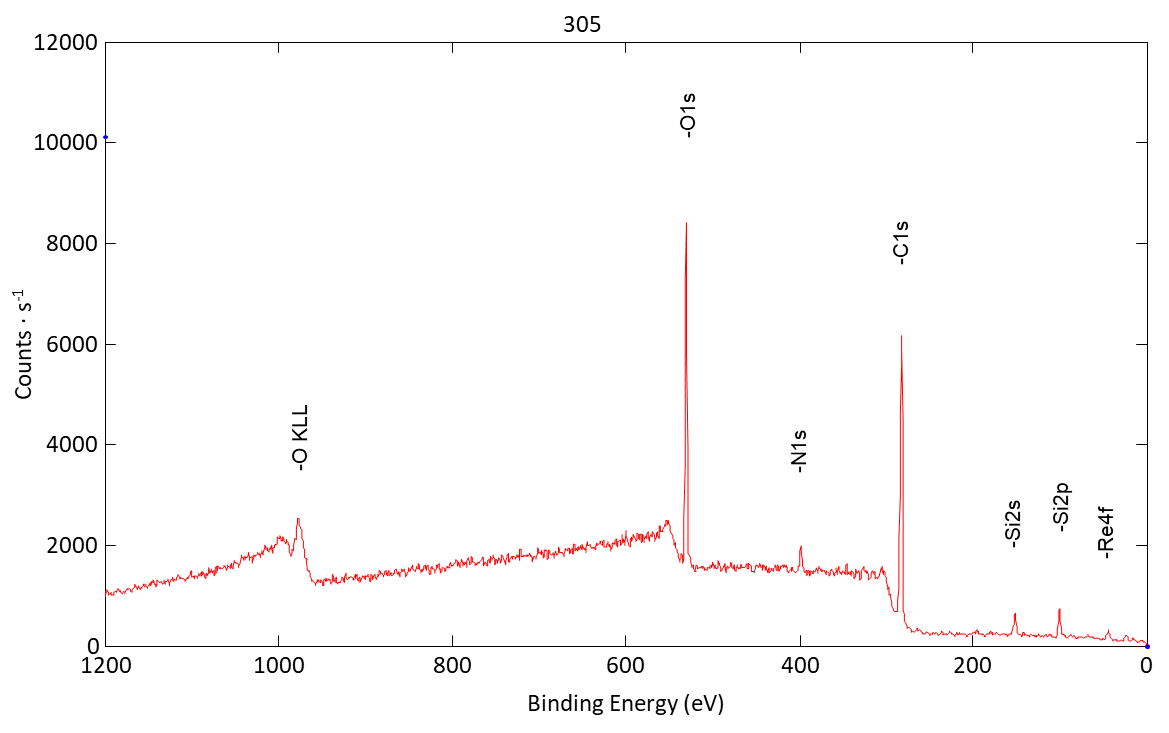
**

**Figure S8.** XPS survey scan of the ^ext^ReHEP sample

**
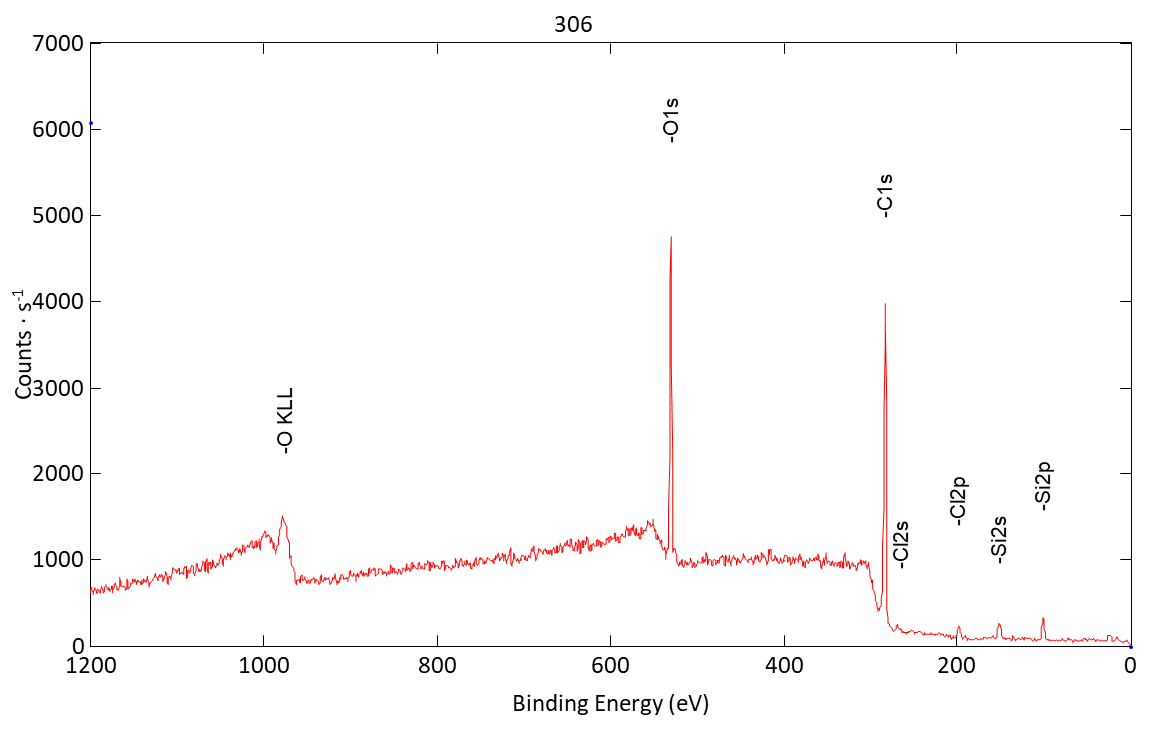
**

**Figure S9.** XPS survey scan of the ^ext^ReAUr sample

**
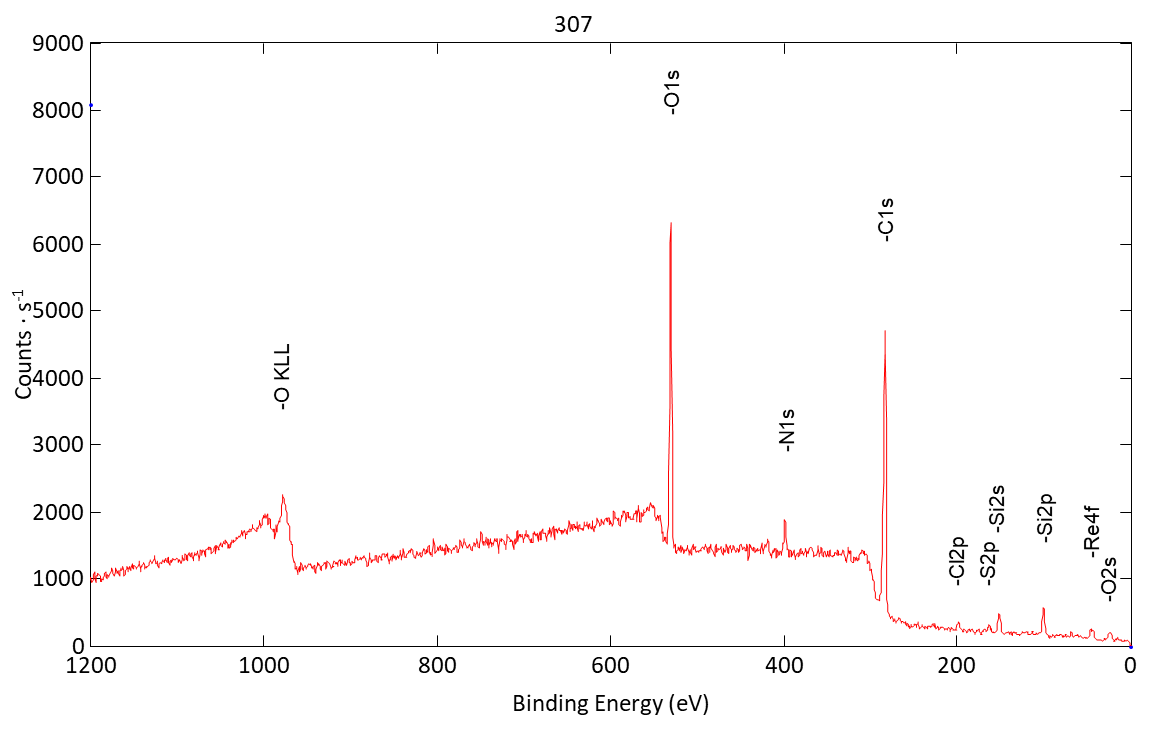
**

**Figure S10.** XPS survey scan of the ^ext^ReAT sample

**
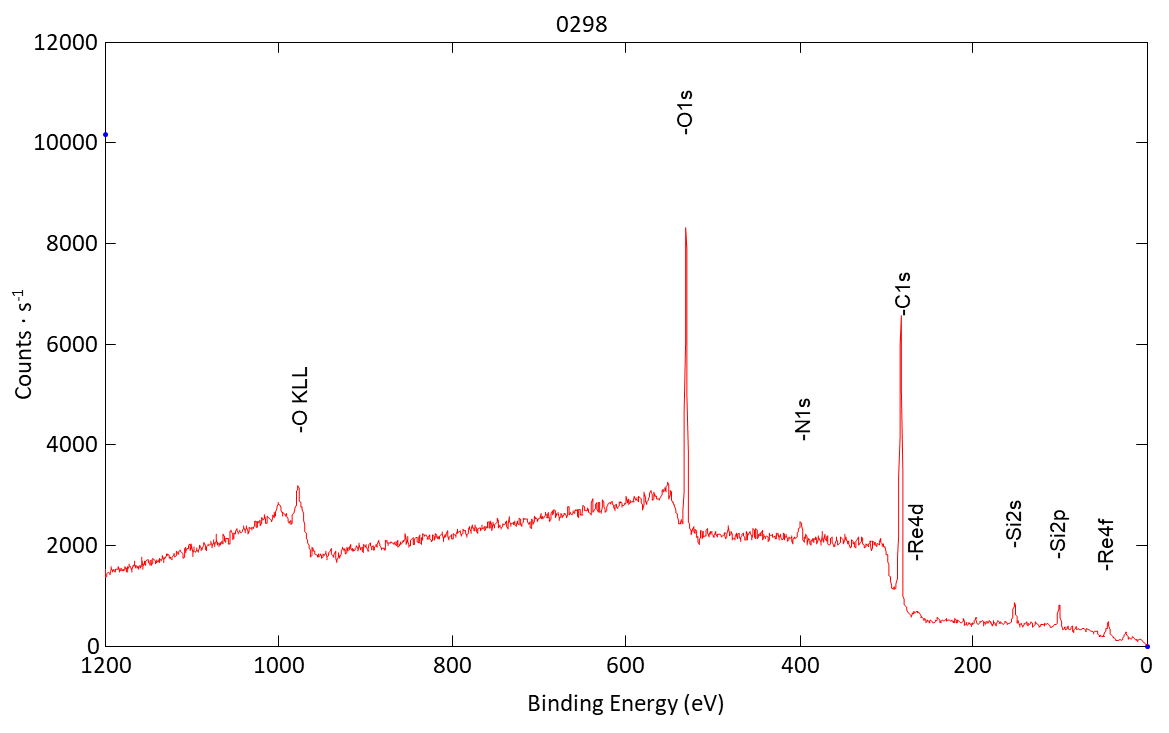
**

**Figure S11.** XPS survey scan of the ReBAPA sample

**
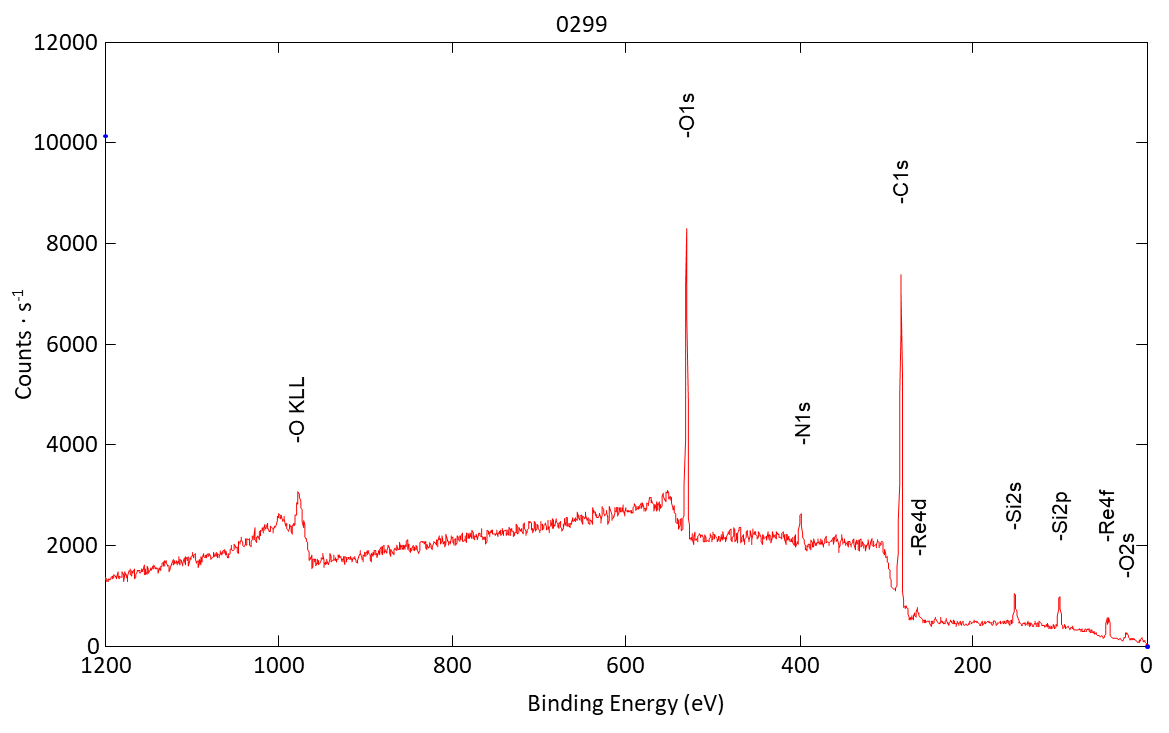
**

**Figure S12.** XPS survey scan of the ReCDi sample

**
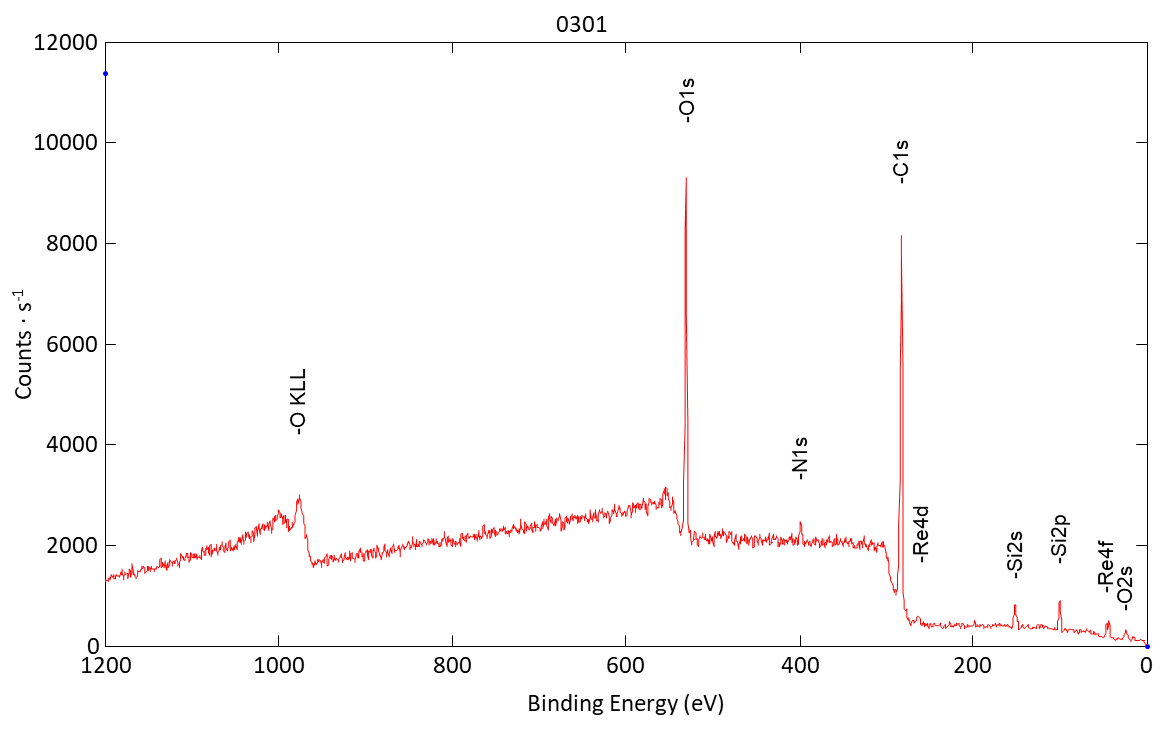
**

**Figure S13.** XPS survey scan of the ReHMI sample

**
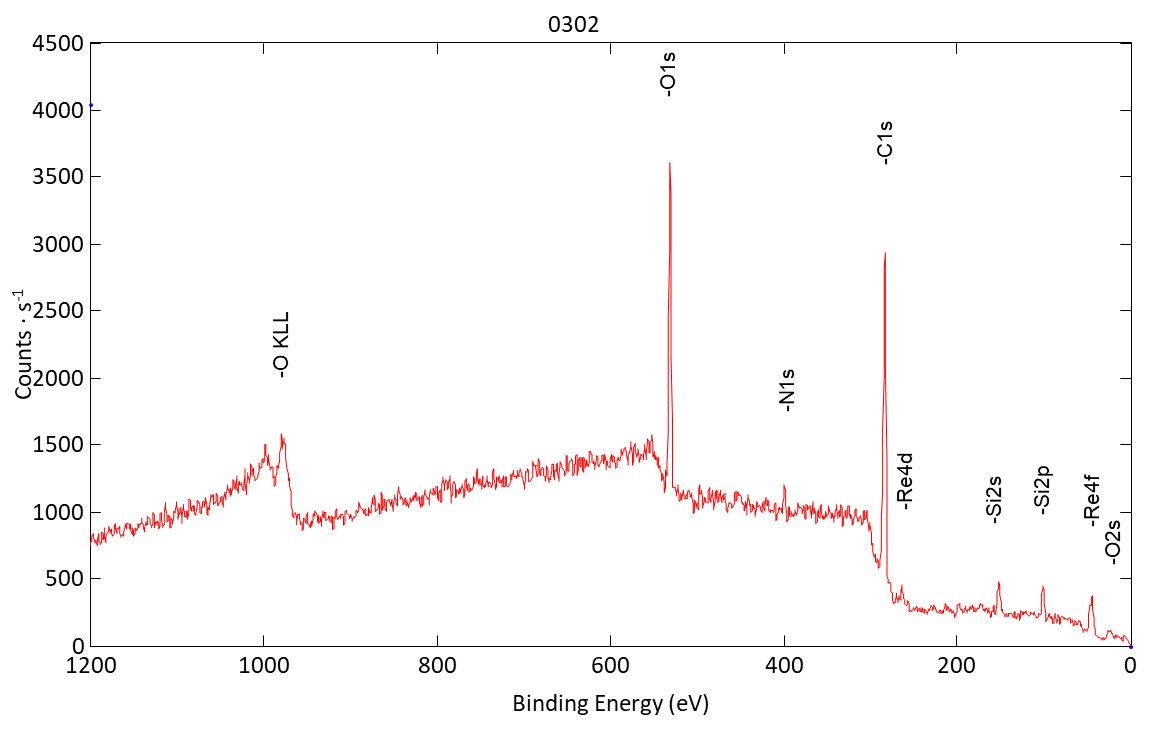
**

**Figure S14.** XPS survey scan of the RePP sample

**
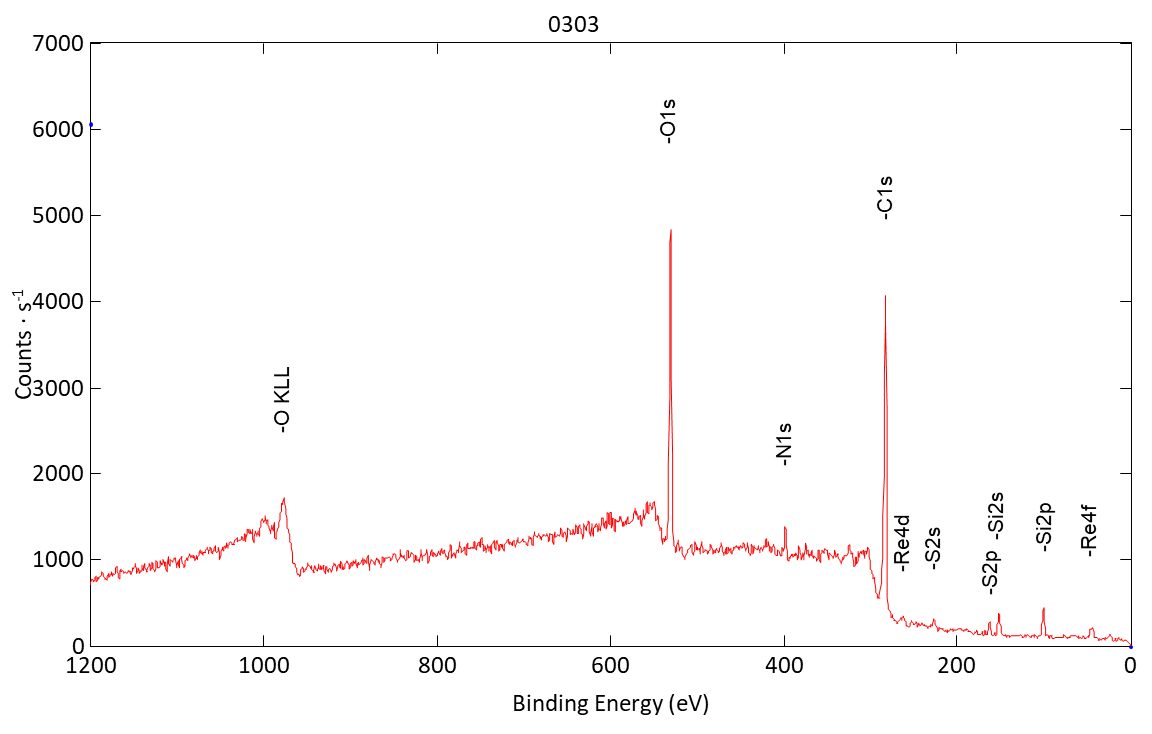
**

**Figure S15.** XPS survey scan of the ReTSC sample

**
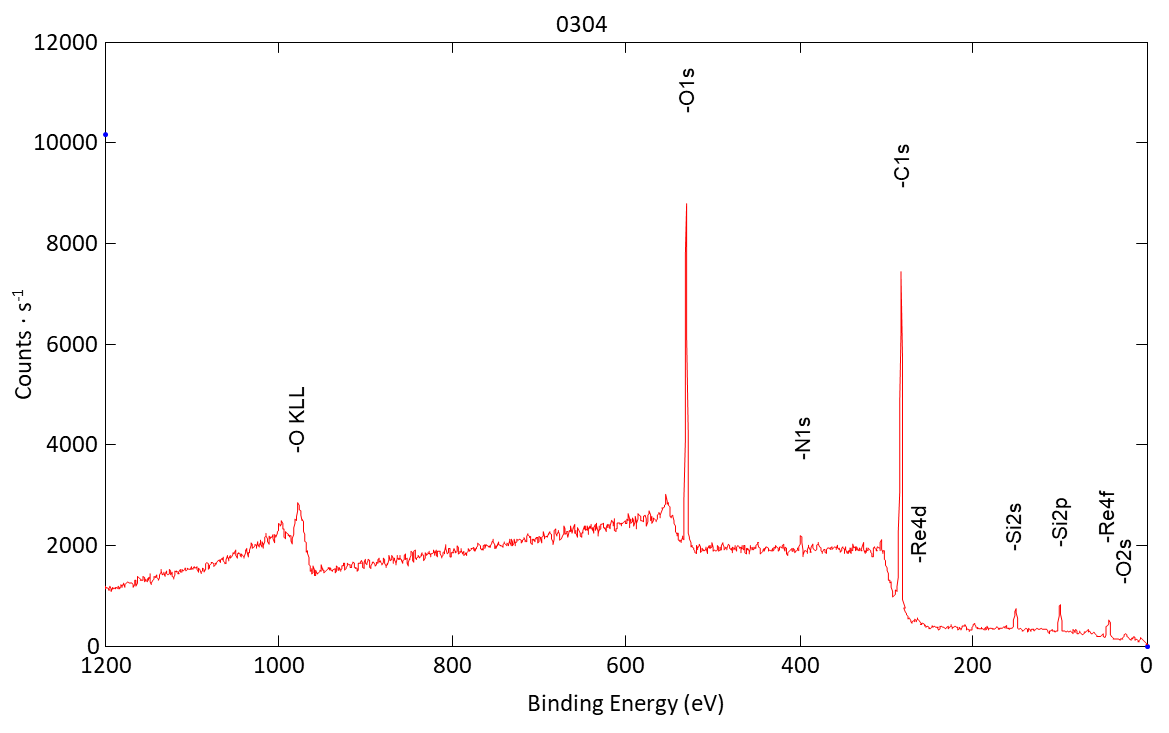
**

**Figure S16.** XPS survey scan of the ReAHP sample

**
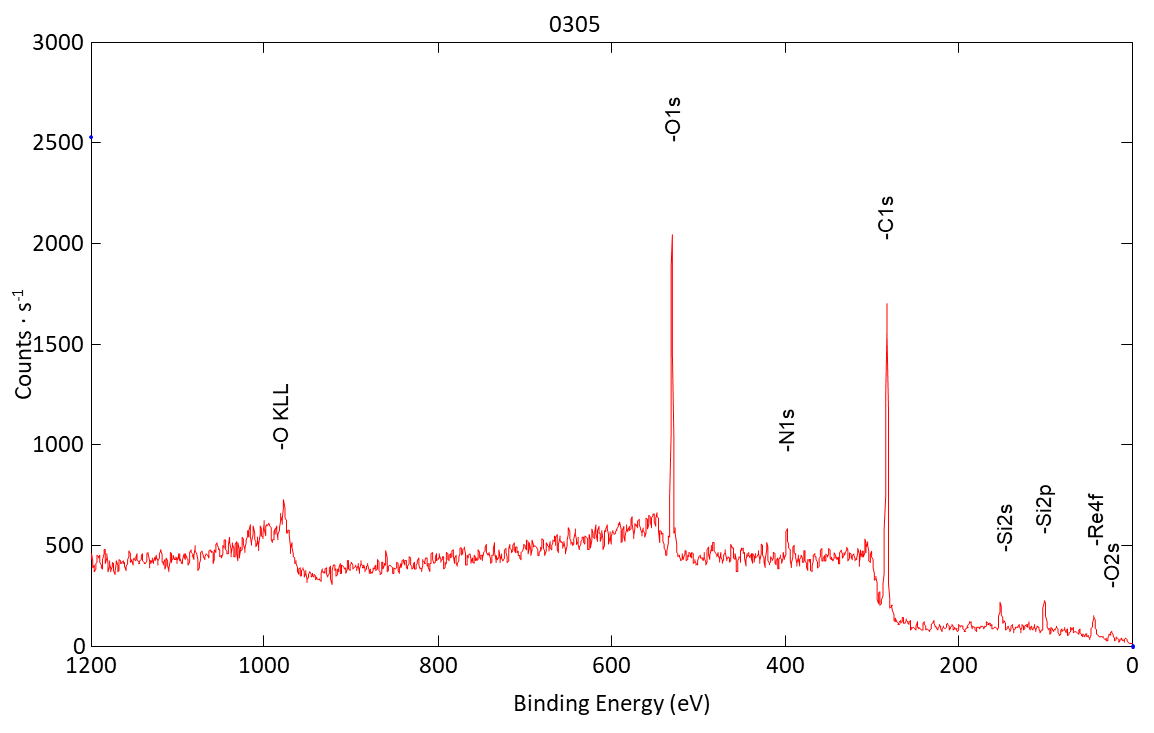
**

**Figure S17.** XPS survey scan of the ReHEP sample

**
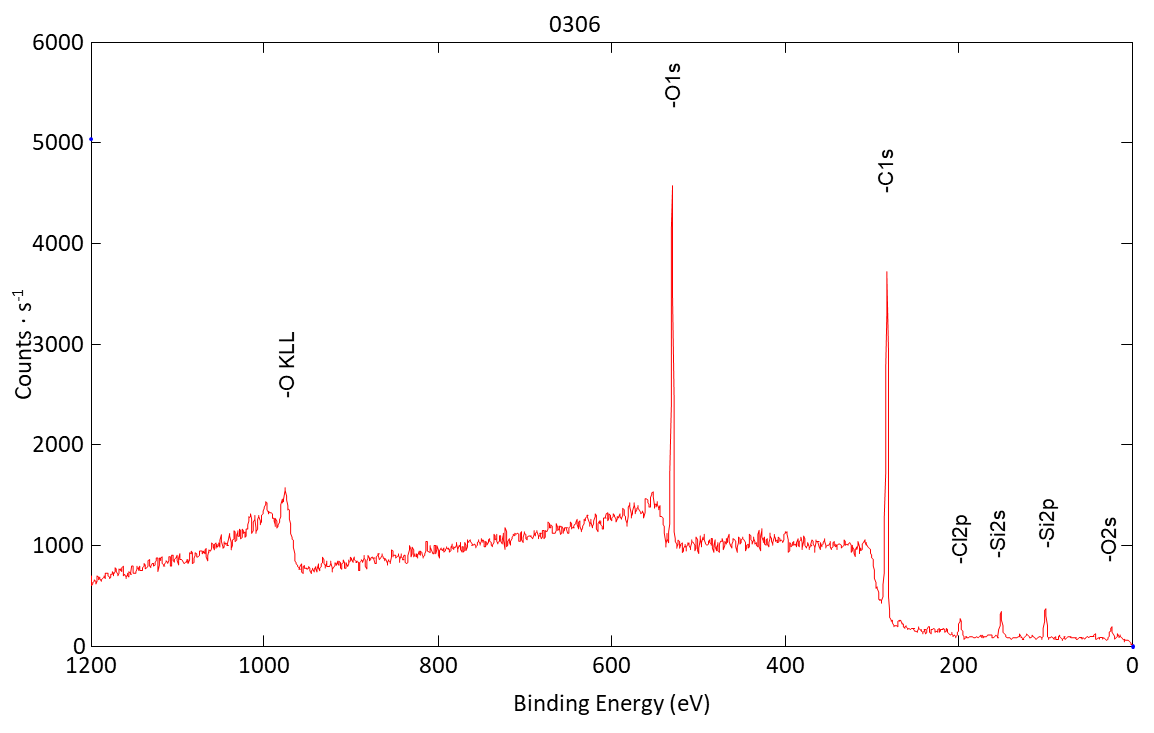
**

**Figure S18.** XPS survey scan of the ReAUr sample

**
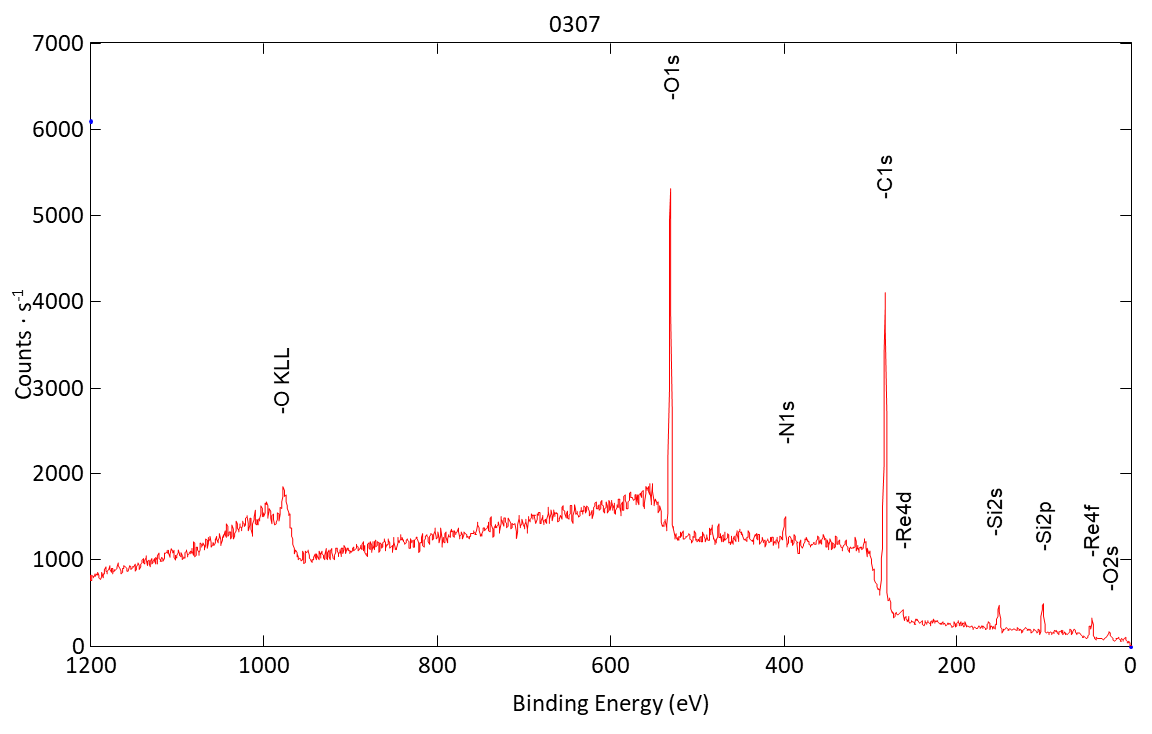
**

**Figure S19.** XPS survey scan of the ReAT sample

**S3.2. XPS high resolution spectra**


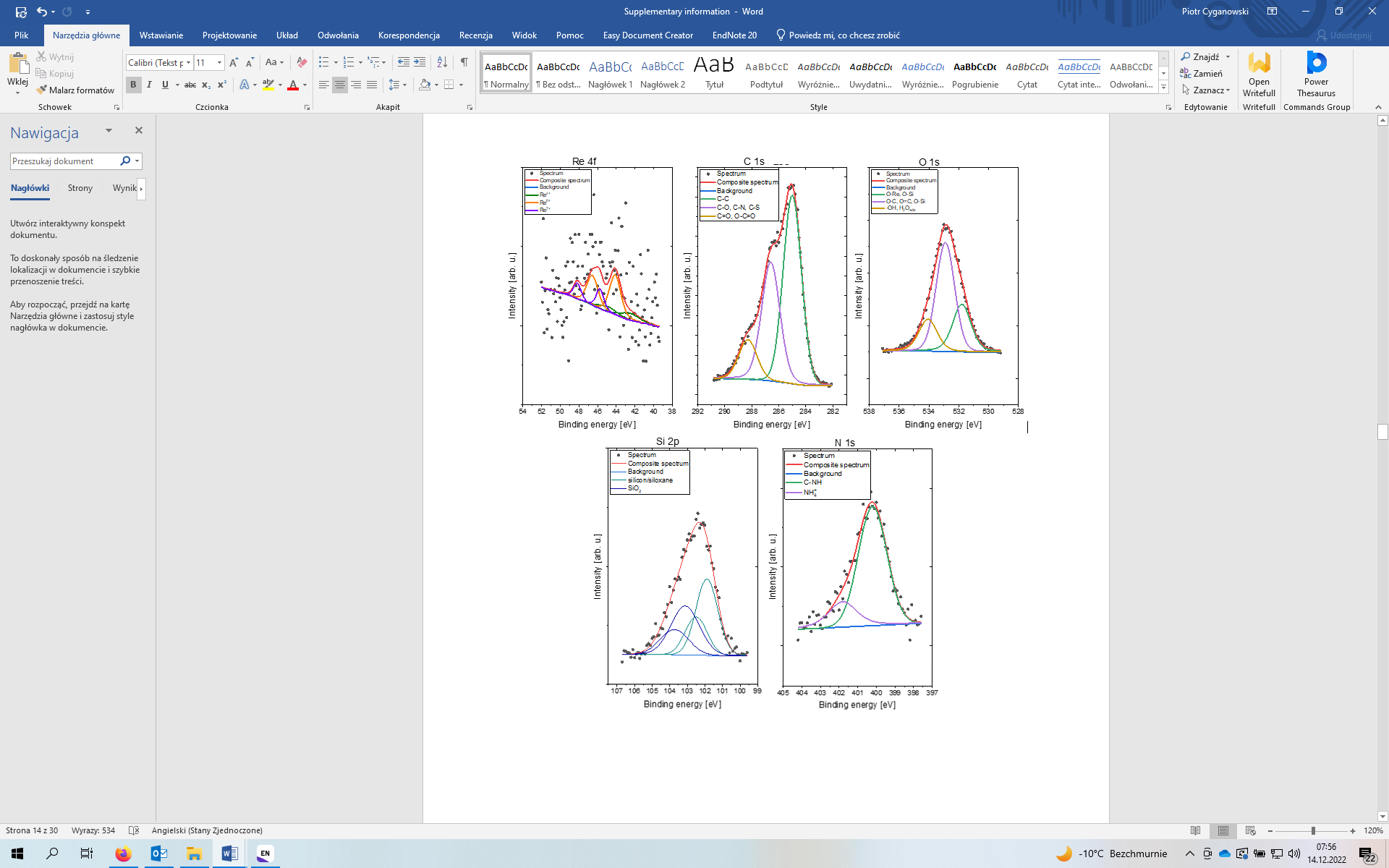


**Figure S20.** XPS high resolution spectra of the ^ext^ReBAPA sample


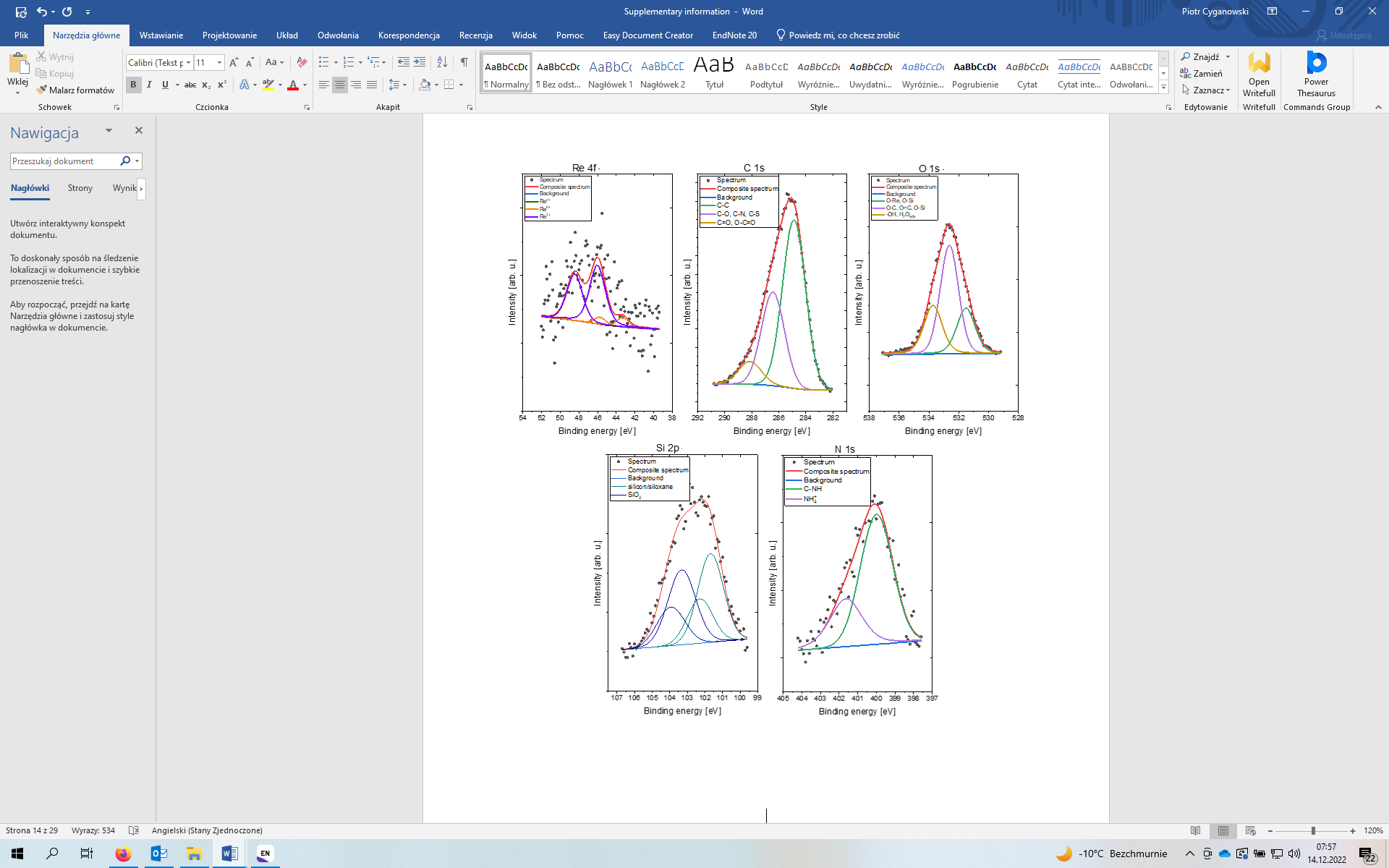


**Figure S21.** XPS high resolution spectra of the ^ext^ReCDI sample


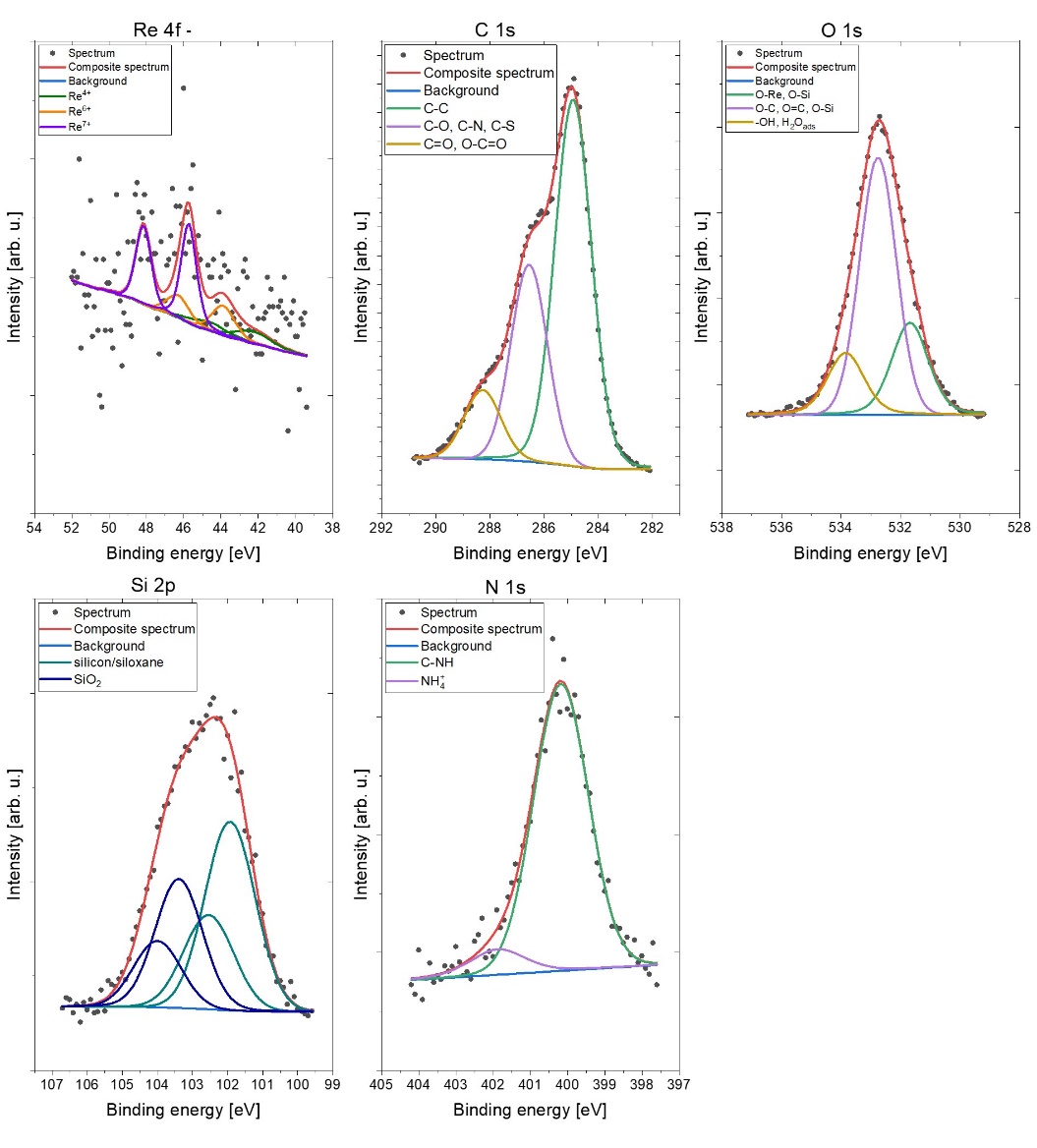


**Figure S22.** XPS high resolution spectra of the ^ext^ReHMI sample


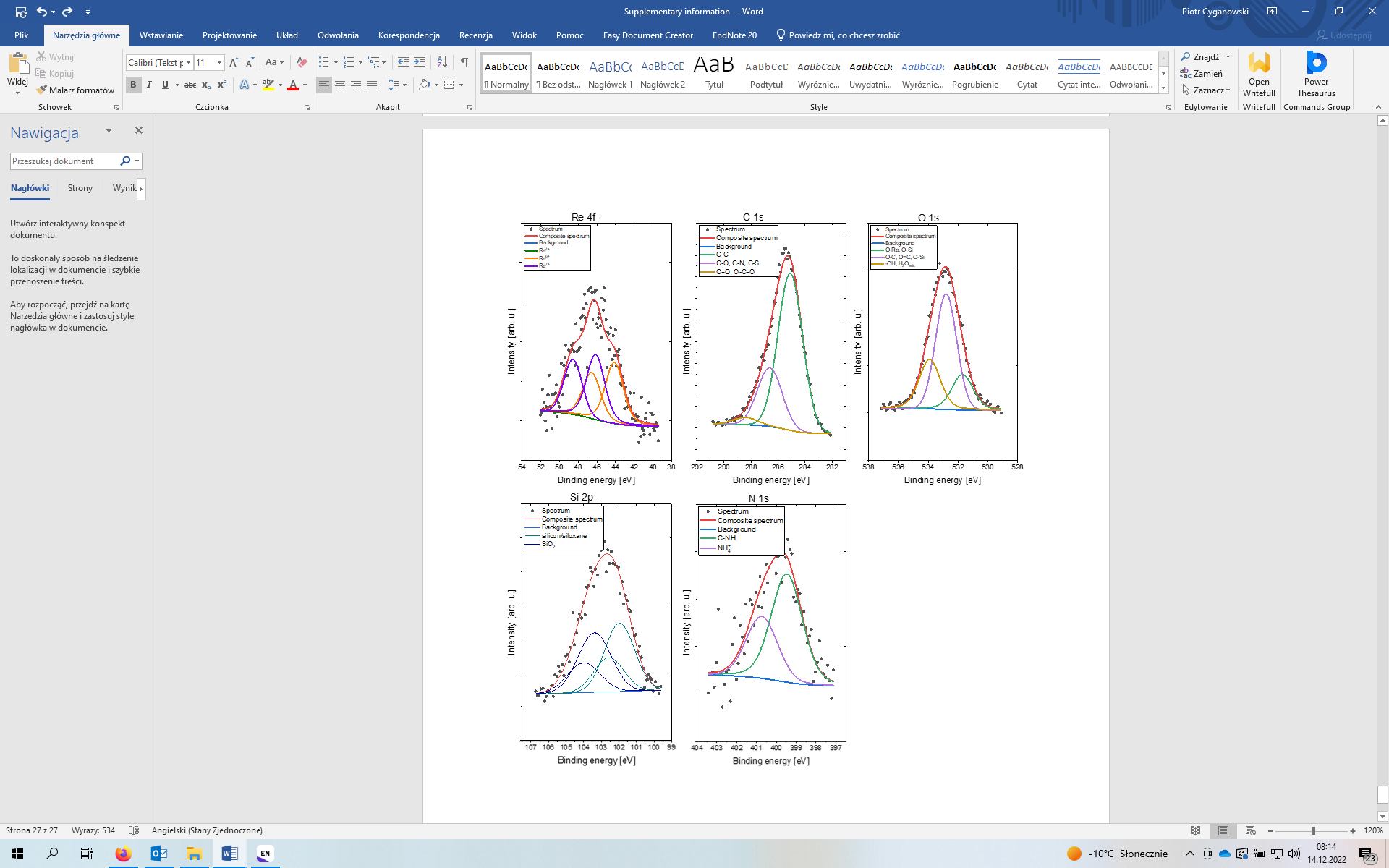


**Figure S23.** XPS high resolution spectra of the ^ext^RePP sample


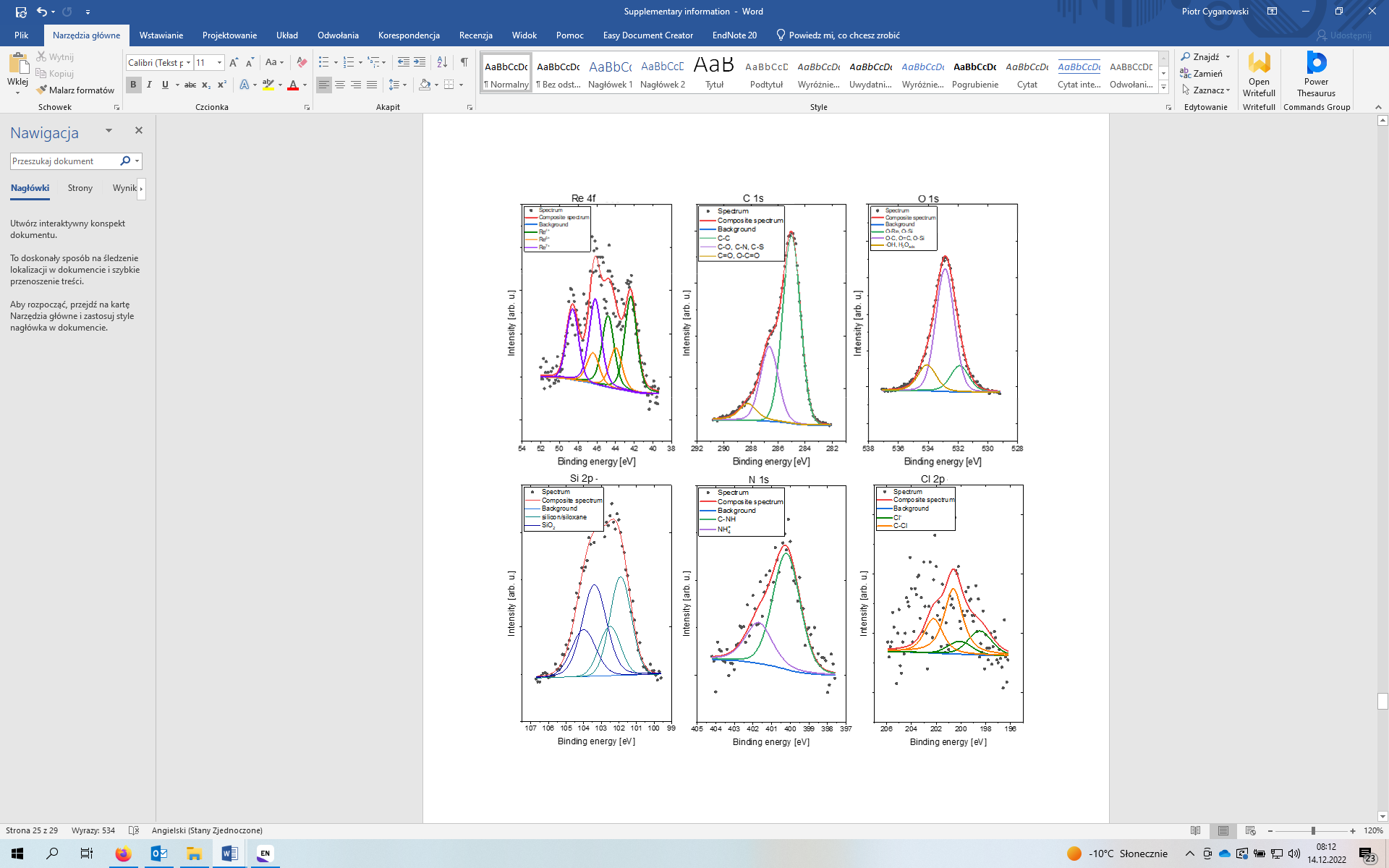


**Figure S24.** XPS high resolution spectra of the ^ext^ReTSC sample


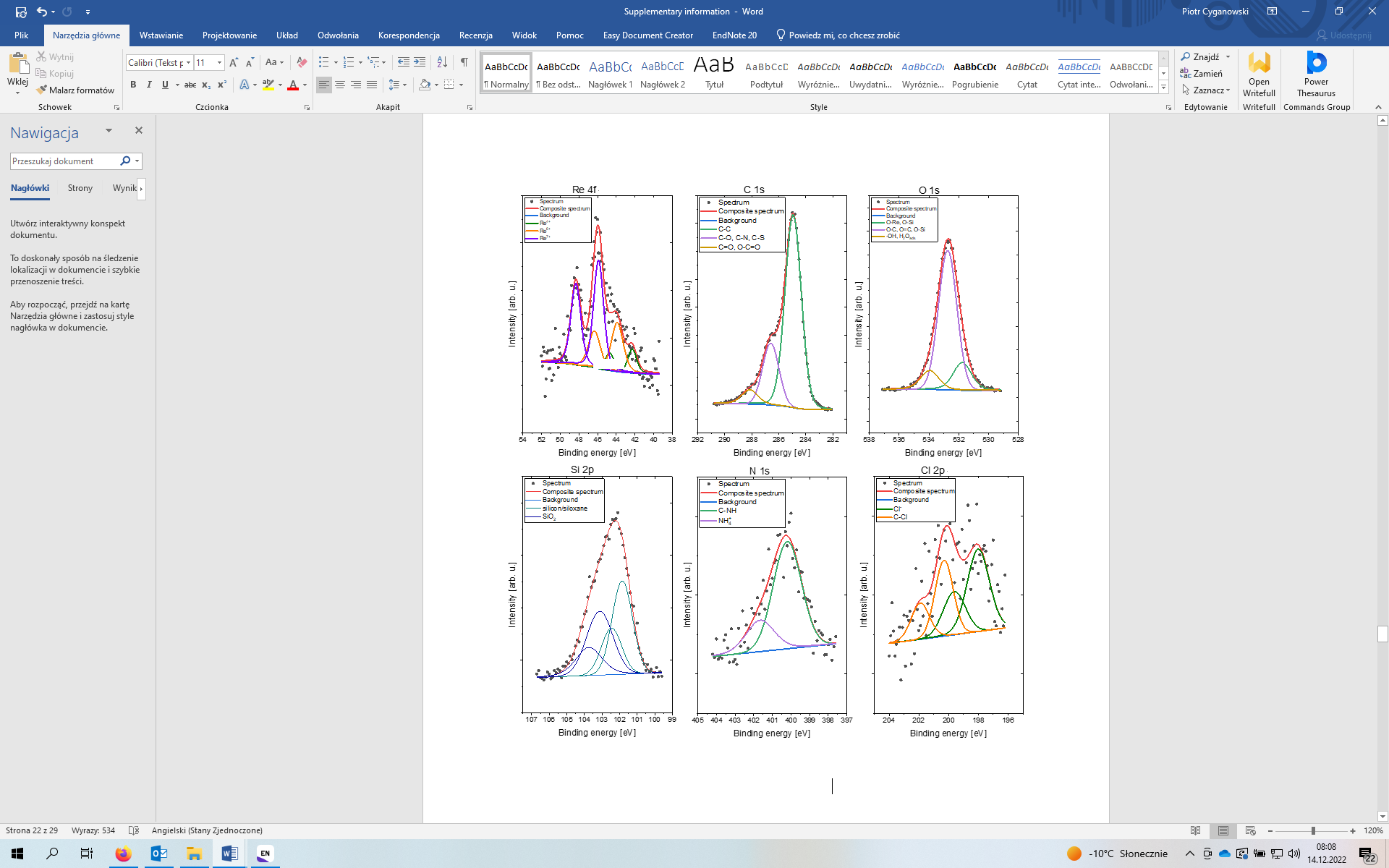


**Figure S25.** XPS high resolution spectra of the ^ext^ReAHP sample


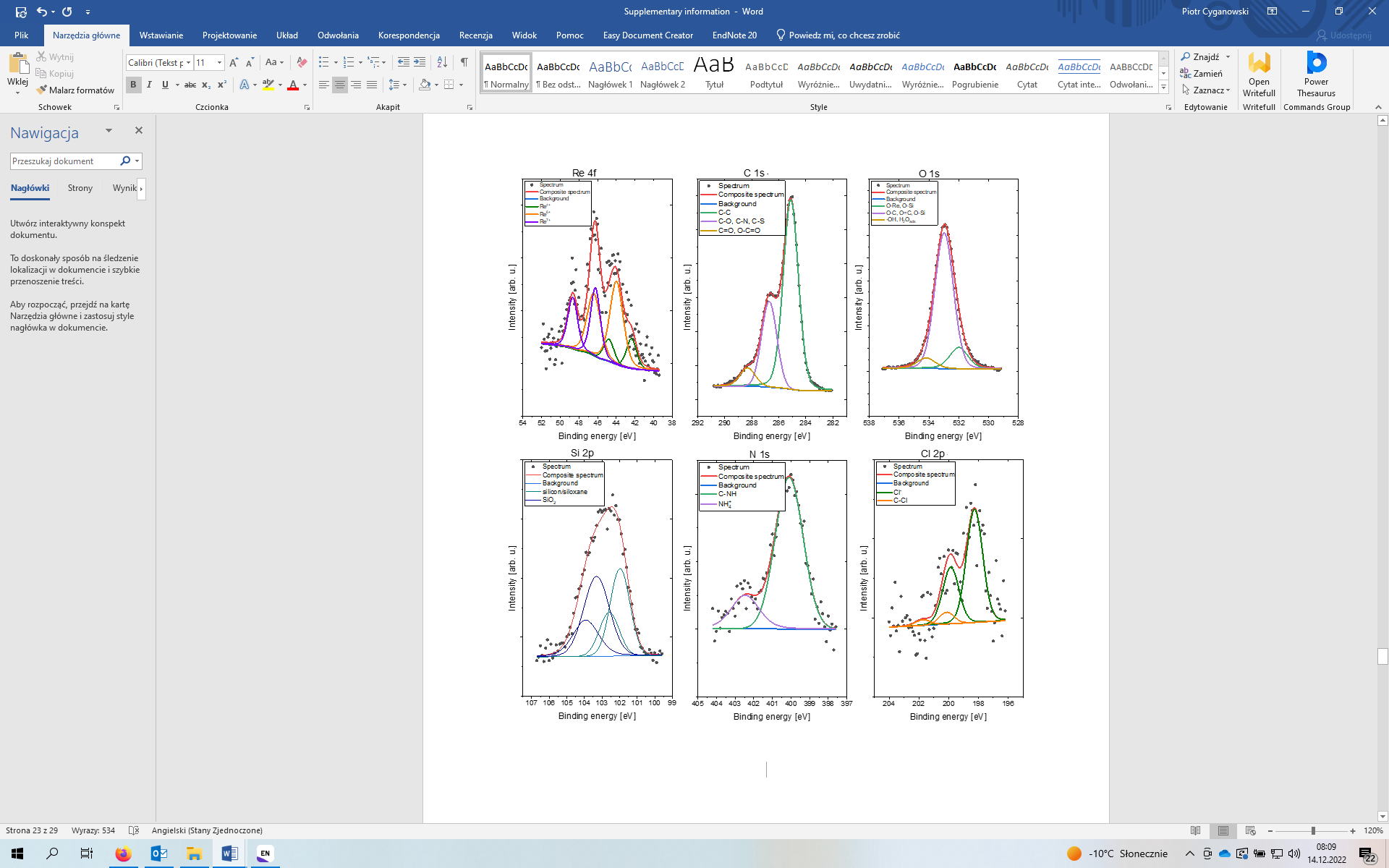


**Figure S26.** XPS high resolution spectra of the ^ext^ReHEP sample


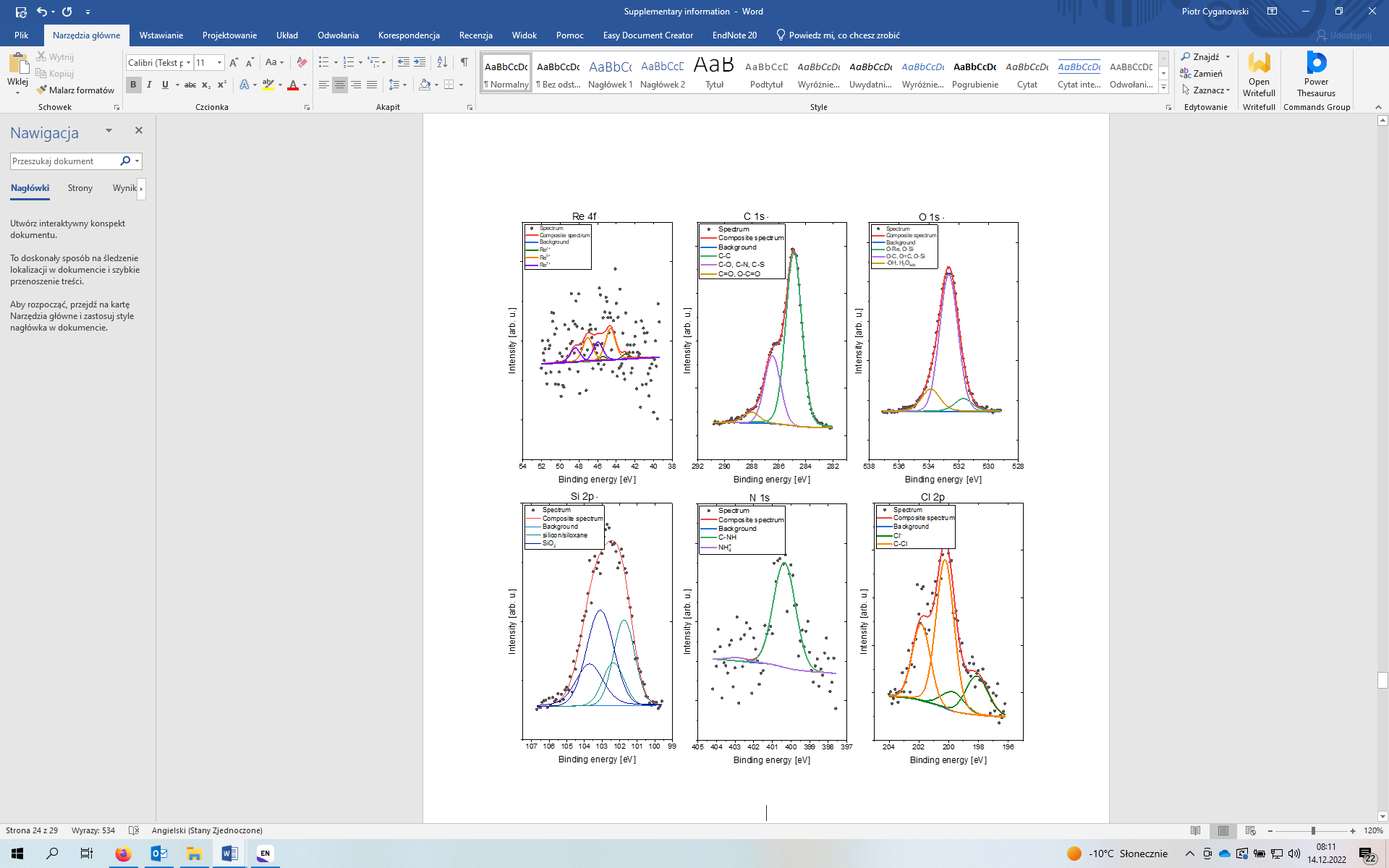


**Figure S27.** XPS high resolution spectra of the ^ext^ReAUr sample


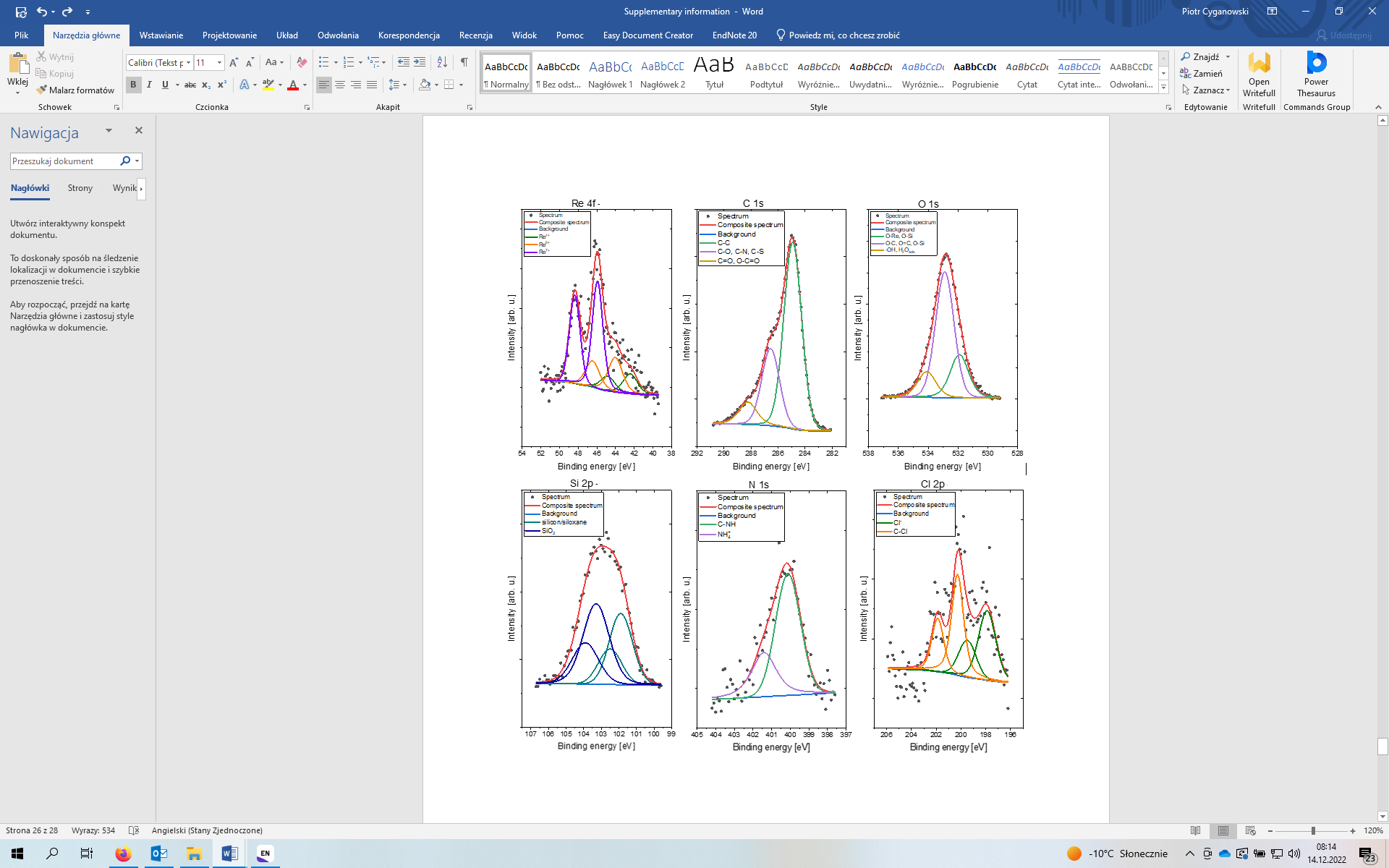


**Figure S28.** XPS high resolution spectra of the ^ext^ReAT sample


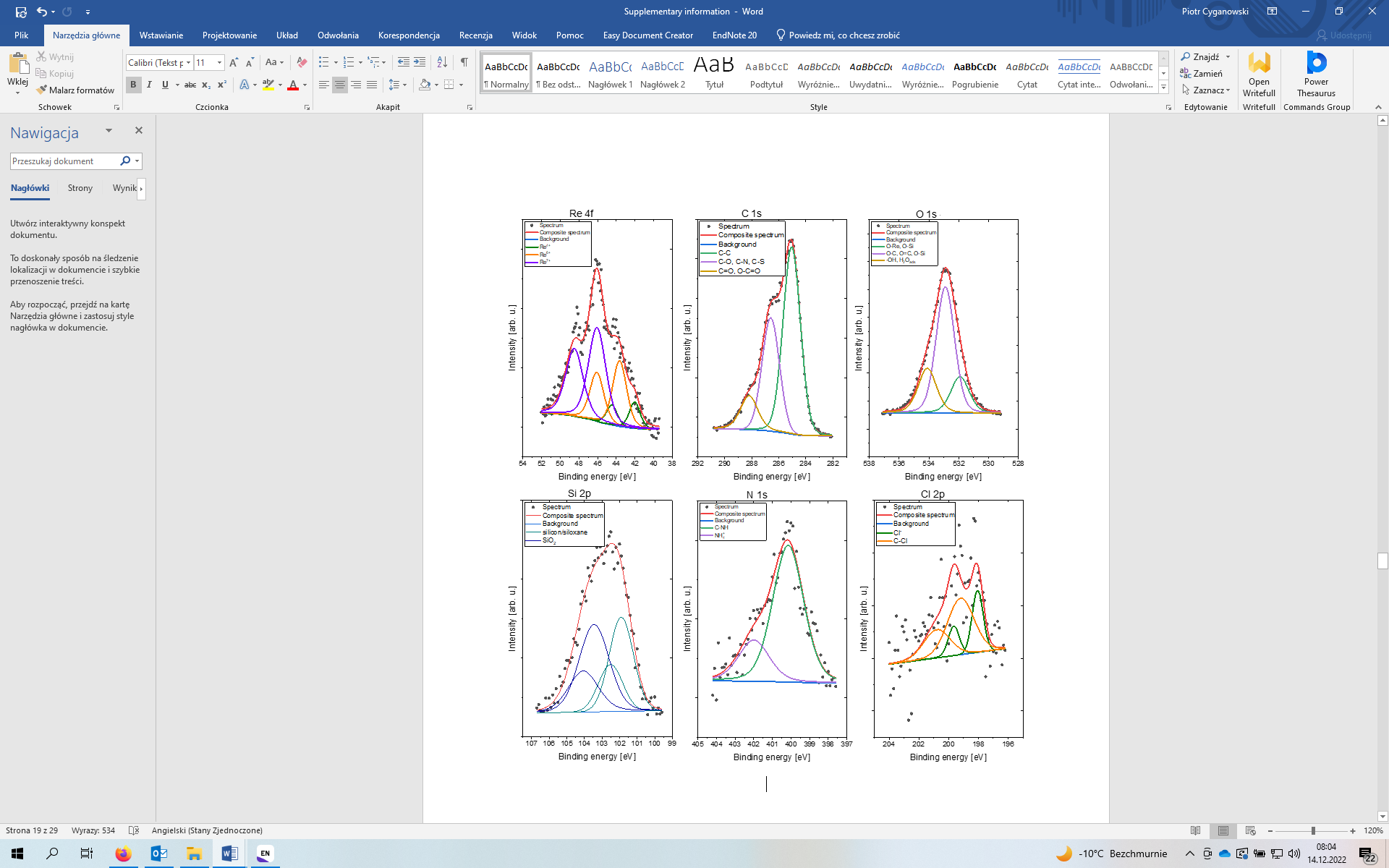


**Figure S29.** XPS high resolution spectra of the ReBAPA sample

**
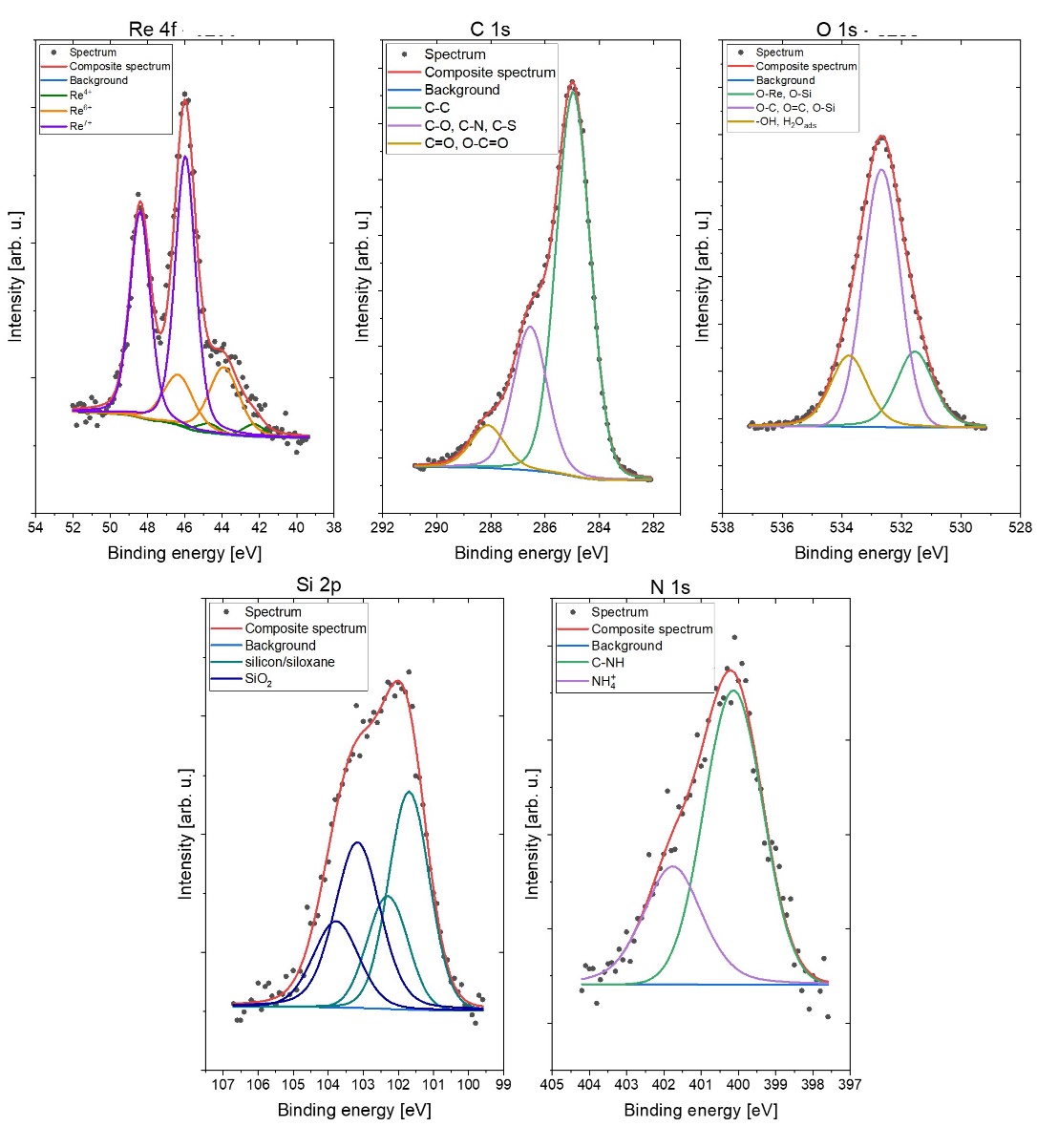
**

**Figure S30.** XPS high resolution spectra of the ReCDI sample


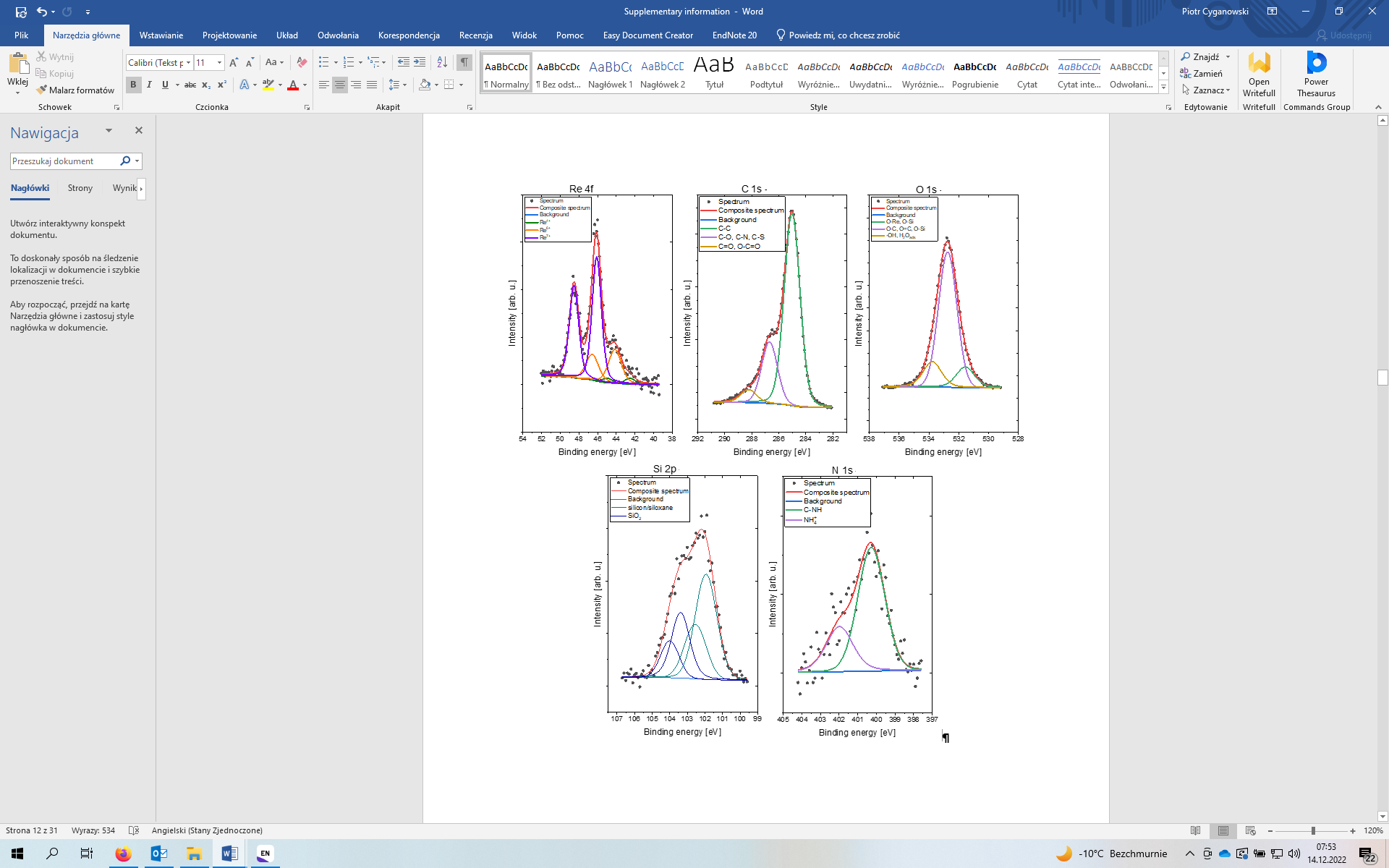


**Figure S31.** XPS high resolution spectra of the ReHMI sample


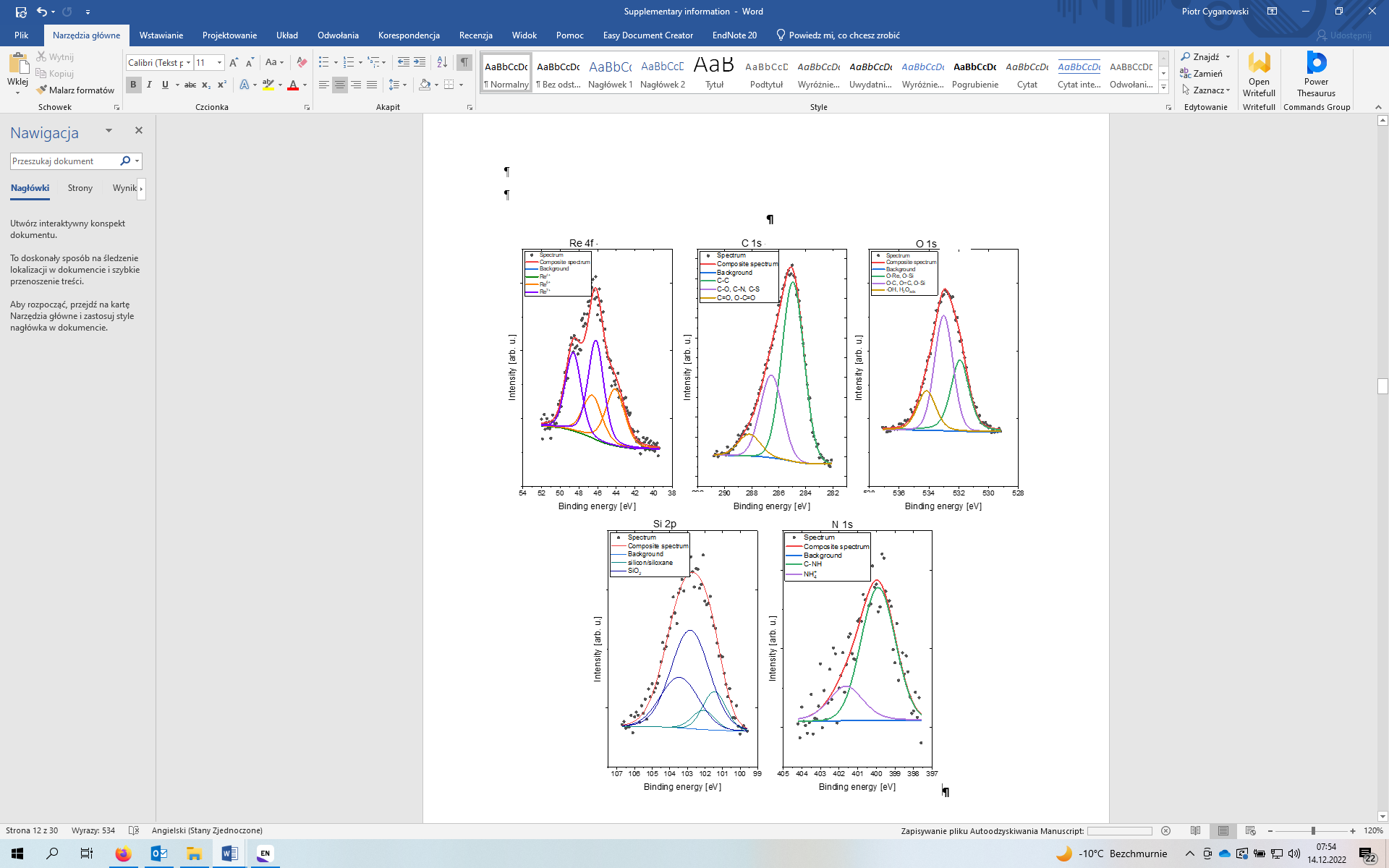


**Figure S32.** XPS high resolution spectra of the RePP sample


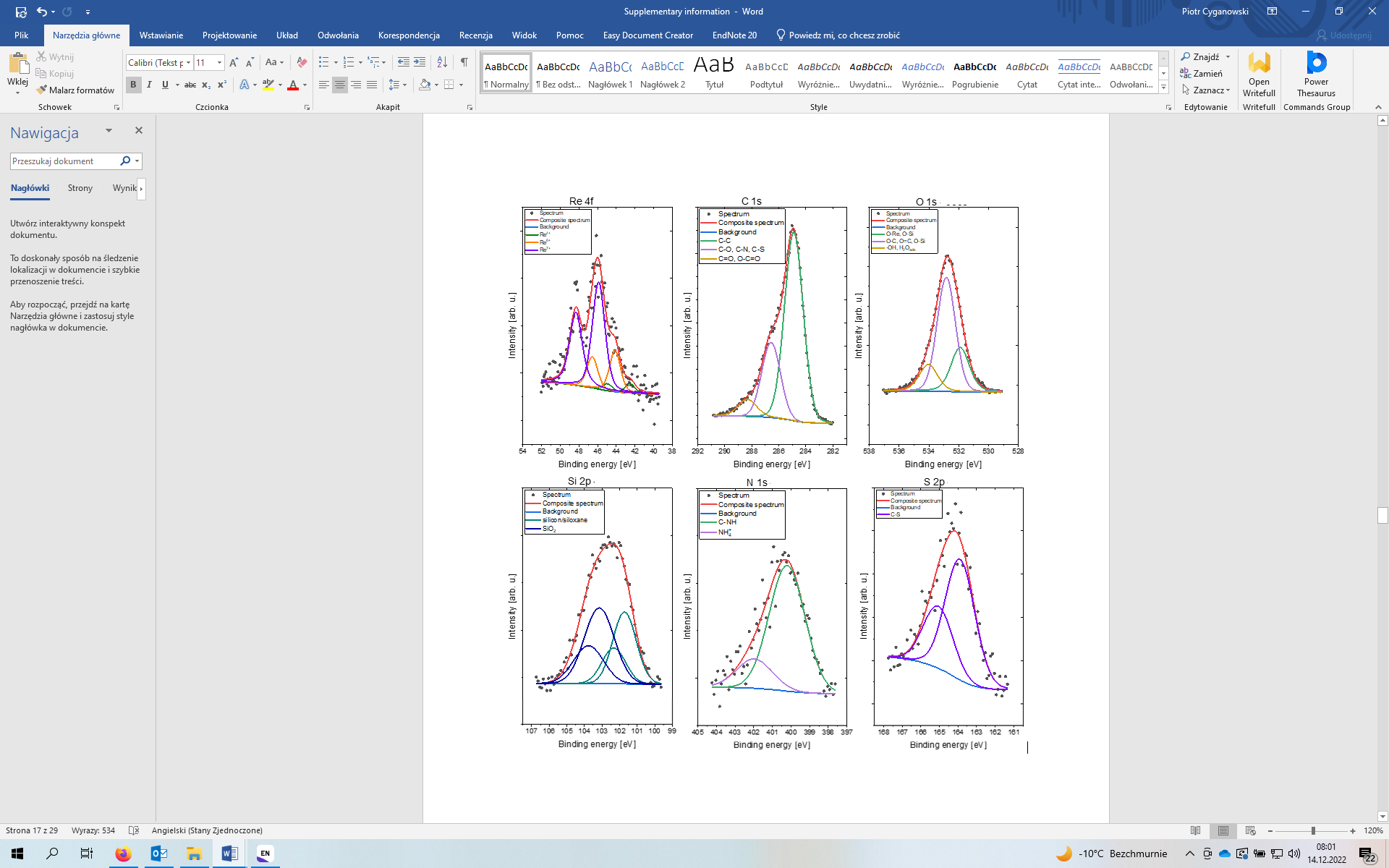


**Figure S33.** XPS high resolution spectra of the ReTSC sample

**
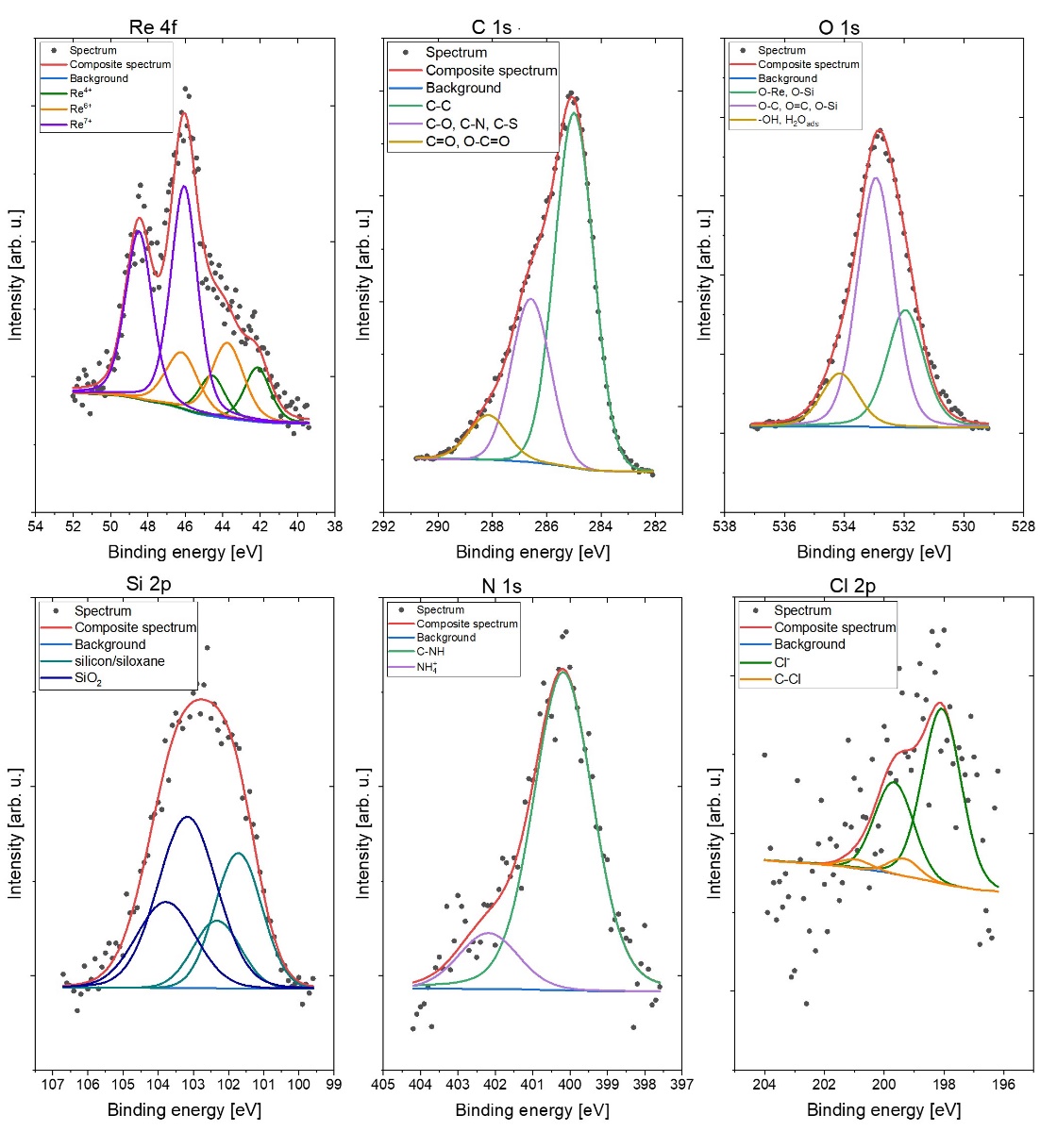
**

**Figure S34.** XPS high resolution spectra of the ReAHP sample


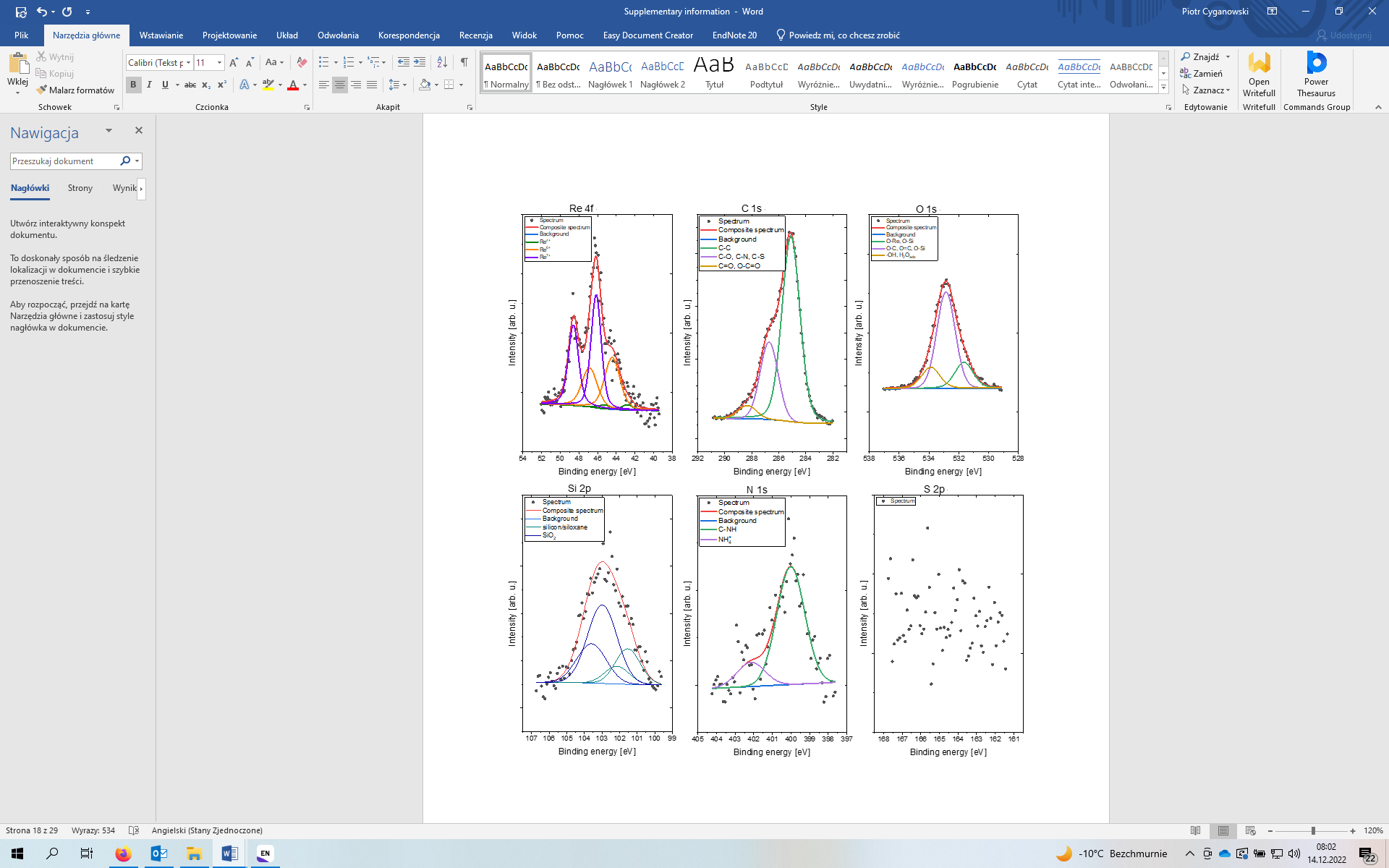


**Figure S35.** XPS high resolution spectra of the ReHEP sample


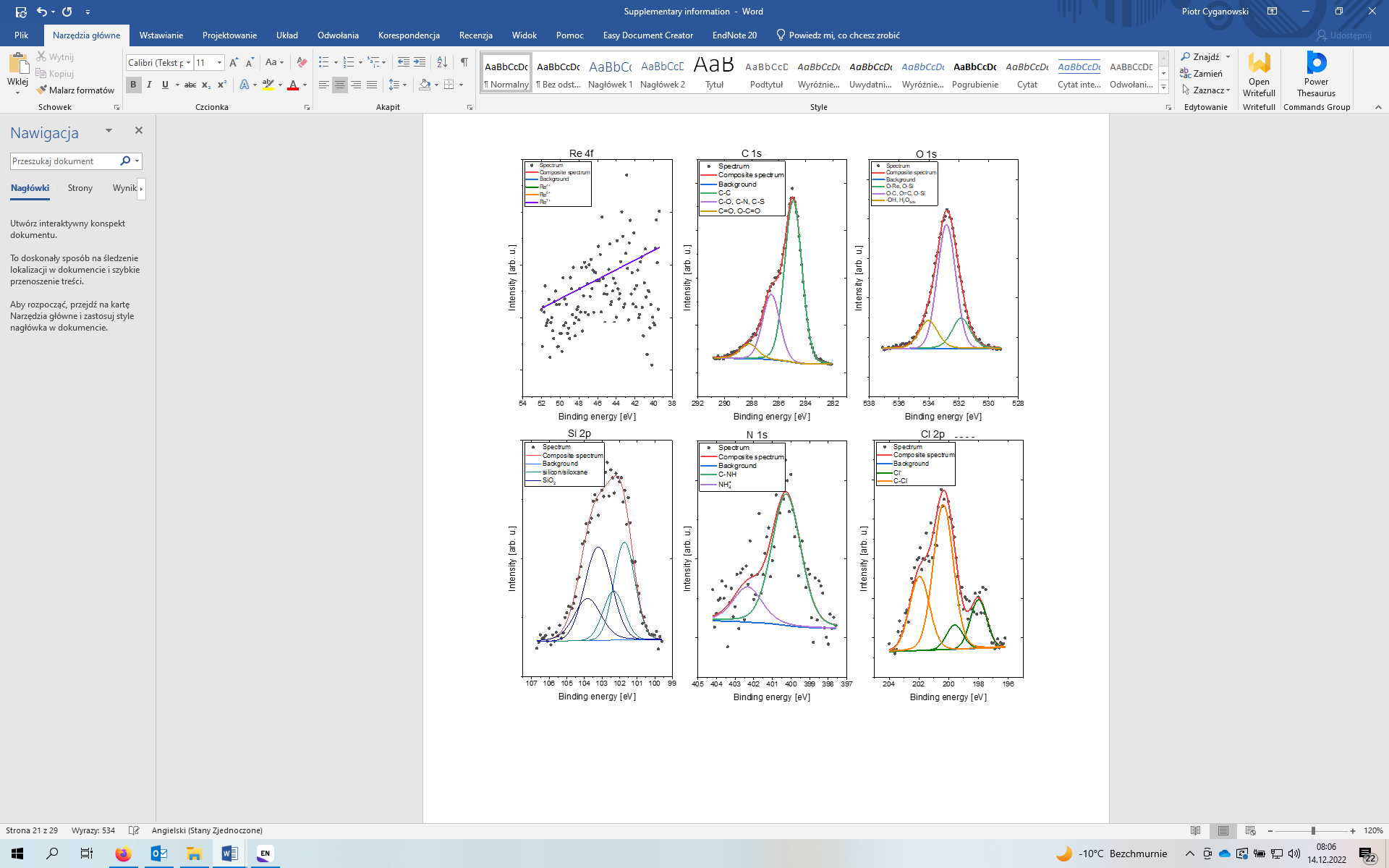


**Figure S36.** XPS high resolution spectra of the ReAUr sample

**
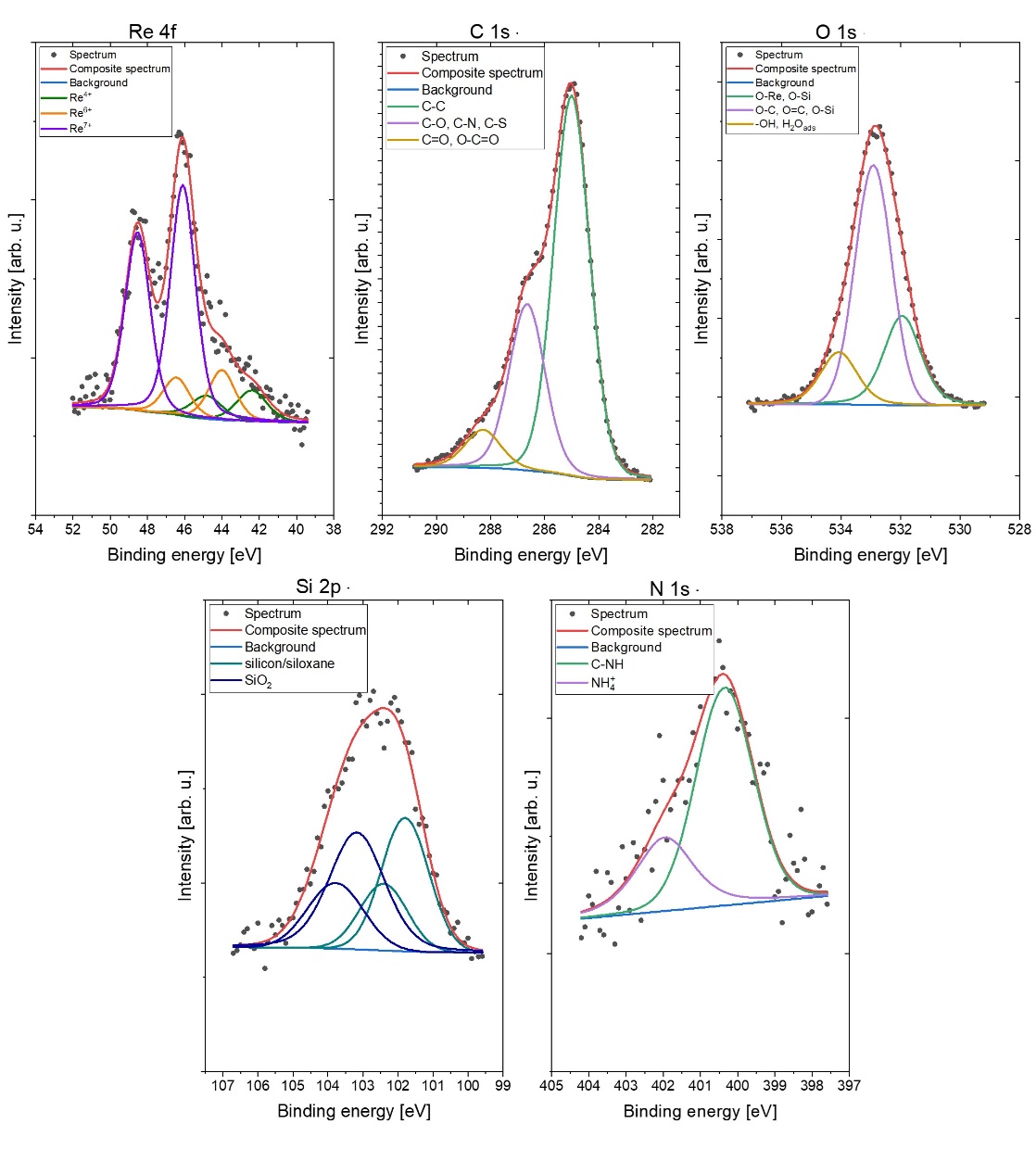
**

**Figure S37.** XPS high resolution spectra of the ReAT sample
